# Metal-centered X-ray absorption and emission spectroscopy of iron corroles: implications for ligand non-innocence

**DOI:** 10.1039/d6sc00669h

**Published:** 2026-03-10

**Authors:** Meiyuan Guo, Abraham B. Alemayehu, Augustin Braun, Sang-Jun Lee, Dimosthenis Sokaras, Edward I. Solomon, Abhik Ghosh, Thomas Kroll

**Affiliations:** a SSRL, SLAC National Accelerator Laboratory Menlo Park California 94025 USA tkroll@slac.stanford.edu meiyuan.guo@kemi.uu.se; b Institute of Chemistry, University of Tromsø 9037 Tromsø Norway abhik.ghosh@uit.no; c Department of Chemistry, Stanford University Stanford California 94305 USA solomone@stanford.edu

## Abstract

Determining the electronic structure of transition metal complexes with non-innocent ligands is challenging, both experimentally and theoretically. In this study, we investigate the electronic structure of iron corrole nitrosyl (Fe[TPC](NO), TPC = *meso*-triphenylcorrole) using a combination of Fe L-edge and K pre-edge X-ray absorption spectroscopy (XAS), Kβ X-ray emission spectroscopy (XES), and multiconfigurational calculations. A key debate revolves around the distribution of radical character on the ligand and the spin/oxidation state of the iron center. The experimental spectra reveal that the Fe center adopts a low-spin configuration, characterized as either Fe^II^ or Fe^III^ with strong π back-bonding and little spin polarization. Calculations identified Fe[TPC](NO) primarily as {FeNO}^6^ with a corrole^3−^, where the {FeNO}^6^ unit exhibits Fe^III^ character. However, no localized hole was found in the iron t_2g_ orbital due to strong covalent mixing with NO π* orbitals. The occupation of the Fe 3d_*z*^2^_ orbital, and thus the radical character on the corrole ligand, is highly sensitive to the axial ligand environment. This was supported by wavefunction analysis over varying Fe–NO distances and comparison with iron corrole chloride (Fe[TPC]Cl), which displayed significant radical character on the corrole ligand due to the weak axial ligand. These findings provide critical insights into ligand non-innocence and covalency in metal corrole systems, offering a foundation for understanding other highly covalent transition metal systems.

## Abstract 2

Metal-centered X-ray spectroscopy and multiconfigurational theory reveal bidirectional charge transfer in iron corroles, with ligand-to-metal and metal-to-ligand interactions governing covalency and ligand non-innocence.

## Graphical abstract

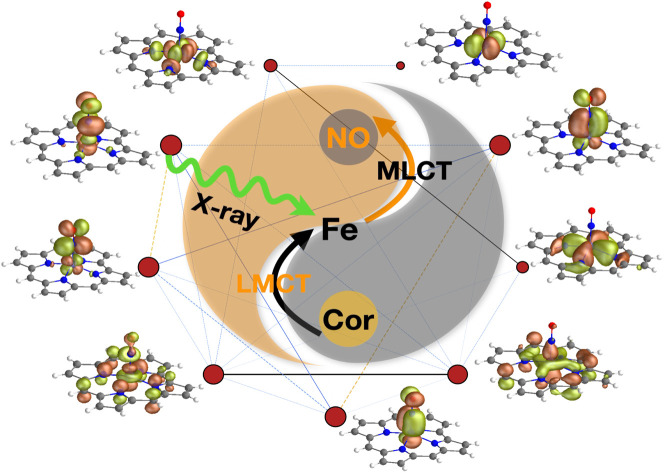

## 1 Introduction

**Fig. 1:**
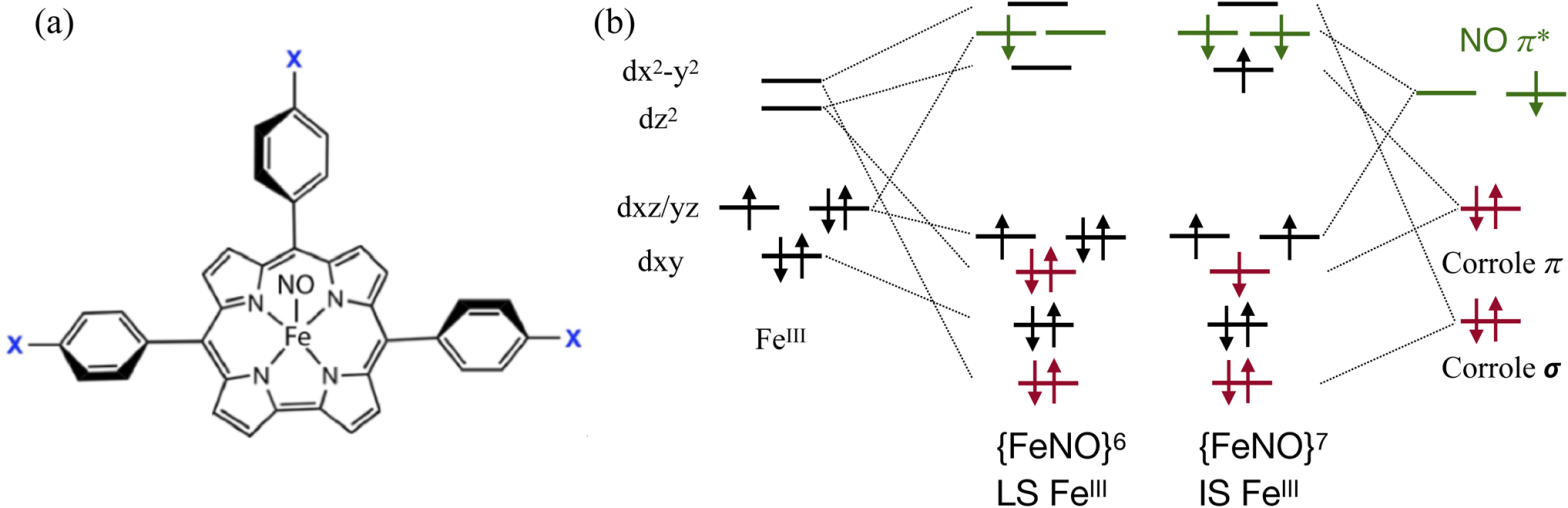
(a) Drawing of the FeNO–corrole molecule, *para*-substituents are denoted as X. In this work, we studied Fe[TPC](NO), *i.e.*, X = H. (b) Schematic representations of molecular orbital diagrams with a low-spin (LS) Fe^III^ center and an intermediate-spin (IS) Fe^III^ center.

Determining the exact electronic structure of 3d transition metal ions can be challenging when the metal 3d and ligand orbitals are heavily mixed. A notorious example of this complexity is iron corrole nitrosyl. Here, the central iron covalently interacts with two potentially non-innocent ligands: the equatorial corrole ligand and the axial NO ligand (Fig. 1(a)). Earlier studies, based on structural data such as bond distances and the nearly linear Fe–N–O bond angle, described these complexes as {FeNO}^6^ with a low-spin Fe^III^ center with a closed-shell corrole^3−^ ligand, see Fig. 1(b).^1^ This assignment was supported by infrared (IR) and electron paramagnetic resonance (EPR) characterization of electrochemical reduction and oxidation products.^1^ Subsequent reports challenged this picture. Optical absorption spectra, which showed substituent-dependent changes (X in Fig. 1(a)), together with alternating corrole bond lengths and broken-symmetry (BS) density functional theory (DFT) results, suggested an {FeNO}^7^ description featuring an intermediate-spin Fe^III^ center (S(Fe) = 3/2), antiferromagnetically coupled to both NO^−^ (*S* = 1) and corrolė^2−^ (*S* = 1/2) radicals, see Fig. 1(b).^2–4^ The implication of significant corrole non-innocence represented a major shift in the interpretation of these systems.

Theoretical descriptions of iron nitrosyls have remained controversial. DFT often yields inconsistent spin-state energetics and spin densities depending on the functional used.^5–7^ For corrole complexes, the contracted N_4_ coordination core enhances metal–ligand covalency due to Fe displacement from the macrocycle plane, further complicating the ground-state electronic structure of iron corrole nitrosyl compounds. The possibility of corrole ligand non-innocence adds another layer of complexity. These challenges have motivated the use of multiconfigurational methods, such as complete active space self-consistent field/second-order perturbation theory (CASSCF/CASPT2), which better capture the multi-reference character inherent in these systems.^8^ A recent study using CASSCF/CASPT2 together with the density matrix renormalization group (DMRG) on the FeNO–corrole molecule with *meso*-trisphenylcorrole ligands (Fe[TPC](NO)) determined the ground-state electronic structure as {FeNO}^7^ with an *S* = 3/2 Fe^III^ center antiferromagnetically coupled to an *S* = 1 NO^−^ and an *S* = 1/2 corrolė^2−^ radical, see Fig. 1(b). Here, the determination from multi-configurational calculation relied on a full expansion of the complete active space configuration interaction (CASCI) with a smaller active space using localized orbitals.^9^ This contrasts sharply with the early determination of a low-spin Fe^III^ derived from EPR characterization of electro-chemically generated products of an iron corrole nitrosyl.^1^ Thus, the precise balance between Fe oxidation/spin state and corrole non-innocence remains unresolved.

At the heart of this controversy is the quantitative description of the radical character on both the corrole ligand and axial NO ligand. Of particular importance are the interactions between the Fe 3d_*z*^2^_ and the corrole a_2u_-π orbitals, and between the Fe 3d_*xz*/*yz*_ and NO π* orbitals. The strong covalent mixing between Fe and the corrole ligand, as well as between Fe and NO, creates a ‘two-way’ charge transfer framework, involving ligand to metal charge transfer (LMCT) as well as metal to ligand charge transfer (MLCT). This dual charge transfer character makes the determination of effective spin density at the iron center challenging and ambiguous.

In this context, X-ray spectroscopy is an optimal tool to directly probe the iron valence orbitals.^10–14^ In X-ray absorption spectroscopy (XAS), an electron of a core level (*e.g.*, Fe 1s or 2p) is excited into an unoccupied (valence) orbital (*e.g.*, Fe 3d or 4p), hence directly probing the unoccupied orbitals on the metal.^15^ X-ray emission spectroscopy (XES) is a complementary method in which a 1s core electron gets excited into the vacuum and the successive decay is observed, *i.e.*, the occupied orbitals are probed. Here, we selected the Fe Kβ decay (3p → 1s) that is highly sensitive to the spin of the Fe ion.^16–20^ We present a comprehensive study using Fe K-edge (1s → 3d, 4p) and Fe L-edge (2p → 3d) XAS and Fe Kβ XES to probe the electronic structure of the iron corrole compound Fe[TPC](NO). The corresponding experimental spectra are calculated and interpreted using multi-configurational calculations showing the validity of the theoretical method and unambiguously determining the dominant Fe orbital configuration. We then discuss the Fe[TPC](NO) ground state in terms the corrole ligand non-innocence character and compare it to Fe[TPC]Cl with a known non-innocent corrole ligand, where Fe^III^*S* = 3/2 antiferromagnetically couples to corrolė^2−^.^21–23^

## 2 Experimental and computational details

### 2.1 Sample preparation

The iron corrole complexes Fe[TPC](NO) and Fe[TPC]Cl are known compounds. The preparation is adopted from previous literature studies.^24,25^ Fe^III^[Pc]Cl (Pc = phthalocyanine) and Fe^II^[Pc] compounds were purchased from Sigma-Aldrich and used without further purification. Fe^II^[TPP](ImH)_2_ (TPP = *meso*-tetraphenylporphyrin; ImH = imidazole) and Fe^III^[TPP](ImH)_2_Cl (Cl^−^ serves as the counterion; to avoid confusion with Fe[TPC]Cl and Fe^III^[Pc]Cl, where chloride is coordinated to the iron center, the complex will be referred to as [Fe^III^[TPP](ImH)_2_]^+^ throughout the text) were prepared as described previously.^12,26,27^

### 2.2 X-ray absorption and emission spectroscopy

The Fe L-edge XAS spectra were recorded at SSRL beamline 10-1. The measurements were performed on solid-state samples. The powdered metal complexes were finely ground, spread onto double-sided conductive carbon tape, and mounted on an aluminum sample holder. The beam damage was assessed by comparing repeated scans collected at the same sample position and no changes in edge position or spectral line shape were observed. Spectra collected at different sample positions were also fully consistent. The Fe L-edge spectra were collected using both fluorescence yield and sample current detection simultaneously. The spectra presented here correspond to the sample current data, which provided the best signal-to-noise ratio. The photon energy was calibrated from the Fe L-edge XAS spectrum of Fe_2_O_3_ run at intervals between scans, aligning the maximum intensity of the Fe_2_O_3_ L_3_-edge to 708.5 eV and L_2_-edge to 720.1 eV. Data were collected over the energy range from 690 eV to 820 eV for normalization and background subtraction of each data set.

Fe K-edge XAS and XES data were collected at SSRL beamline 15-2, which is equipped with a liquid nitrogen cooled double-crystal monochromator (Si(3,1,1)), allowing for high resolution of incoming radiation. X-ray absorption spectra were recorded using the High Energy Resolution Fluorescence Detection (HERFD) mode with the emission energy placed on top of the Fe K_1_ spectrum. Fe Kα emission was detected using two Ge (440) analyzer crystals arranged in a 1 m Rowland geometry, while for Fe Kβ emission five Ge (620) crystals were used. A single element silicon drift detector was used to measure the X-ray emission signal. The emitted beam path was enclosed by a He-filled bag to reduce signal attenuation. To reduce photo-damage, a liquid He cooled cryostat for measurements at 10 K was used, as well as a sample stage that is equipped with motors to allow for horizontal and vertical movement for multiple sampling positions. The effects of photo-damage were carefully monitored and excluded by reducing the photon flux through beam attenuation using Al foil. Incident energies were calibrated against the first inflection point at 7111.2 eV of an internal Fe foil standard, while the emission energies were calibrated using the elastic peaks in the two energy ranges.

### 2.3 Multi-configurational calculations of the ground state

**Fig. 2:**
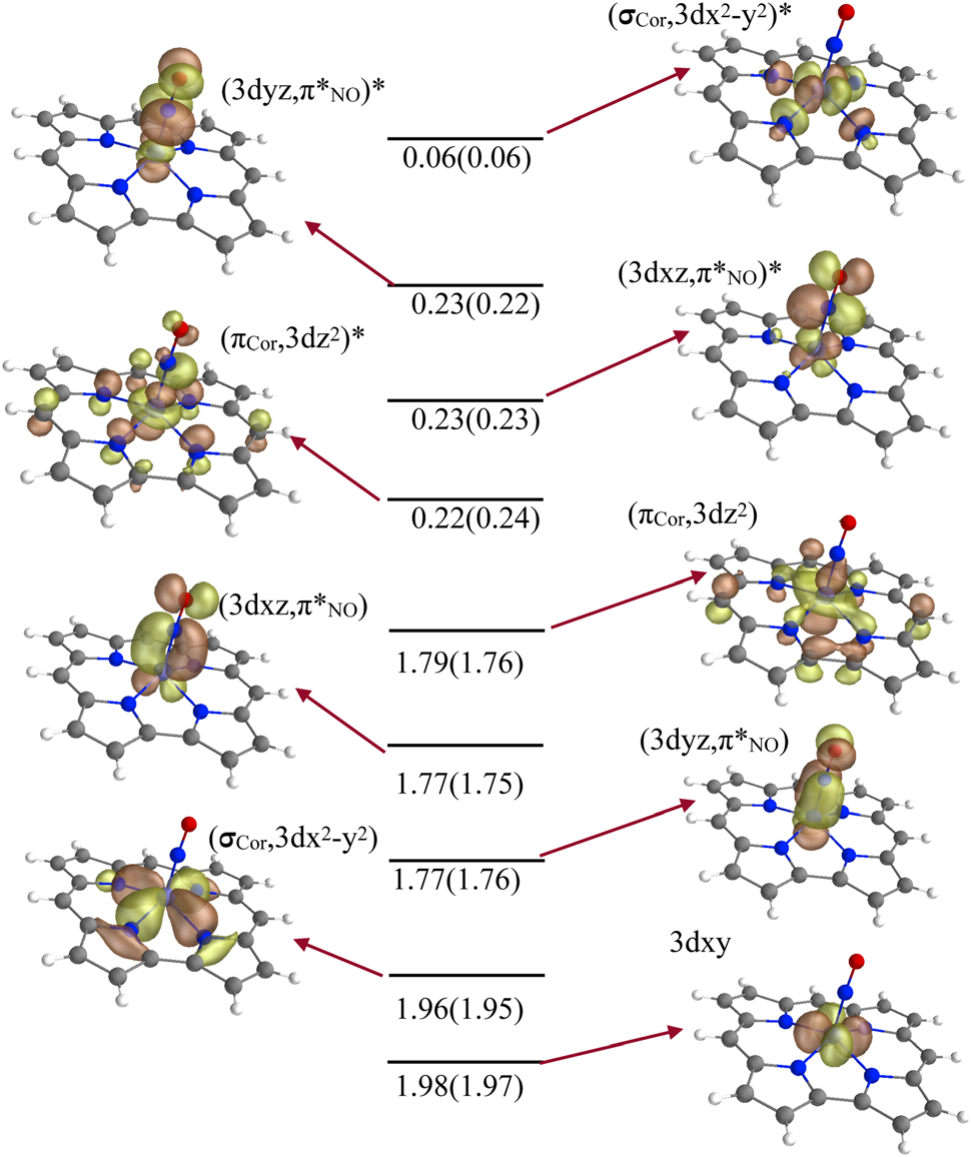
The valence molecular orbitals included in the X-ray spectra calculations. The occupation numbers from active spaces CAS(10;9) and CAS(10;14) are included.

The ground-state wavefunction analysis was performed using the complete active space self-consistent field (CASSCF) method. Fe[TPC](NO) has a singlet ground state; the initial active space consisted of five metal 3d character orbitals and four ligand character orbitals (two NO π* orbitals involved in covalent interaction with metal 3d_*xz*_ and 3d_*yz*_ orbitals, the corrole (a_2u_-π) orbital involved in covalent interaction with the metal 3d_*z*^2^_ orbital, and one σ ligand character orbital included to correlate with the 3d_*x*^2^−*y*^2^_ metal character orbital). This active space is designated as CAS(10;9), as shown in Fig. 2. The orbital pair (σ_cor_, 3d_*x*^2^−*y*^2^_) and (σ_cor_, 3d_*x*^2^−*y*^2^_)* describes the interaction between the 3d_*x*^2^−*y*^2^_ orbital and the corrole ring σ orbital in the ground state. The orbital pairs 
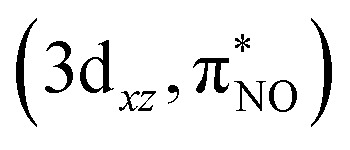
 and 
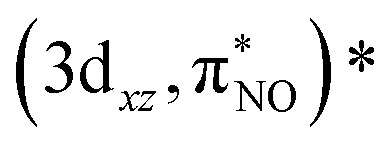
, and 
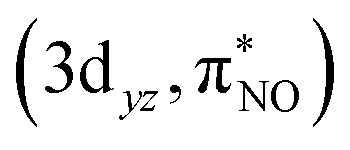
 and 
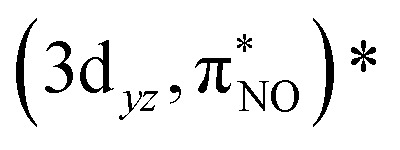
 describe the interaction between the 3d_*xz*_/3d_*yz*_ and the π* orbital of the NO ligand. The orbital pair (π_cor_, 3d_*z*^2^_) and (π_cor_, 3d_*z*^2^_)* describes the interaction between 3d_*z*^2^_ and the corrole ring (a_2u_-π) orbital. The interaction of (π_cor_, 3d_*z*^2^_) and (π_cor_, 3d_*z*^2^_)* can be assigned as ligand-to-metal charge transfer (LMCT), while the interaction between the 3d_*xz*_/3d_*yz*_ and the π* orbital of the NO ligand can be assigned as metal-to-ligand charge transfer (MLCT). The CAS(10;9) active space was later expanded by adding five empty iron 4d character orbitals to describe the double shell effect, resulting in a larger active space CAS(10;14). Both active space calculations provide very similar ground-state wavefunctions and orbital occupations in the valence orbitals. Active orbitals with occupation numbers from active spaces CAS(10;9) and CAS(10;14) (without showing the 4d character orbitals) are shown in Fig. 2. The CASSCF calculation on the [Fe[TPC](NO)]^−^ anion (doublet, {FeNO}^7^) was also performed. Their active orbitals with occupation numbers are provided in Fig. S1.

The active space for Fe[TPC]Cl is slightly different, as it does not include NO ligand π* orbitals. Instead, it includes one more σ ligand character orbital to describe the orbital interaction between the Fe 3d_*z*^2^_ and axial Cl ligand. This active space is designated as CAS(10;8). Again, the active space was expanded by adding the five empty iron 4d orbitals to describe the double shell effect, resulting in a larger active space, CAS(10;13). The active orbitals for Fe[TPC]Cl together with the orbital occupation numbers are available in the Fig. S2.

### 2.4 Multi-configurational calculations of X-ray spectroscopy

Calculations of the Fe L-edge XAS and Fe K-pre-edge XAS spectra were performed using the restricted active space (RAS) method within OpenMolcas.^28^ The active space is designated as RAS(*n*, *l*; *i*, *j*), where *i* and *j* are the number of orbitals in the RAS1 and RAS2 subspaces, respectively, *n* is the number of electrons in the total active space, and *l* is the maximum number of holes allowed in RAS1. Here, the core orbitals (1s or 2p) are placed in RAS1, while the valence orbitals are placed in RAS2. This gives the active space RAS(16,1;3,9) for Fe L-edge XAS and RAS(12,1;1,9) for K pre-edge XAS calculations of Fe[TPC](NO). For Fe[TPC]Cl, RAS(16,1;3,8) and RAS(12,1;1,8) are used for calculating the K pre-edge and L-edge XAS spectra, respectively. The valence active orbitals of Fe[TPC](NO) are shown in Fig. 2.

The core-excited states were calculated with a projection technique called highly excited state (HEXS),^29–31^ which sets the configuration interaction coefficients of configuration state functions (CSFs) with doubly occupied core orbitals to zero, effectively projecting them out of the wavefunction, thus saving computational cost. RASSCF wavefunction optimizations were performed using the state average formalism, which means that the same orbitals were used for all states of a specific spin and symmetry.^32^

Dynamic correction was treated at the level of second-order perturbation theory (RASPT2).^33^ Scalar relativistic effects were included using a second-order Douglas–Kroll–Hess Hamiltonian,^34,35^ in combination with the ANO-RCC basis set and the use of a Cholesky decomposition approach to approximate the two-electron integrals.^36–38^ A new method to account for dynamic correction named multiconfiguration pair-density functional theory (MCPDFT) was also used.^39,40^ This method has advantages of computational efficiency and less dependence on the size of the active space to describe dynamic correlation.^41^ The electric dipole oscillator strengths, including spin–orbit coupling (SOC), were calculated using the RAS state-interaction approach.^42,43^

To compare with experimental spectra, energy shifts of 6.0 eV and 13.6 eV were applied to the Fe L-edge XAS and K pre-edge XAS spectra, respectively. The simulated L-edge XAS spectra were convoluted using a combination of Gaussian and Lorentzian broadening. Gaussian broadening with a width of 0.2 eV was applied uniformly, while Lorentzian broadening with half-width at half-maximum (HWHM) values of 0.2 eV and 0.4 eV was used for the Fe L_3_ and L_2_ edges, respectively. For the simulated K pre-edge XAS, a Gaussian broadening of 0.5 eV was applied.

## 3 Results and analysis

In this section, we first describe the observations from multiple experimental X-ray measurements for Fe[TPC](NO). For comparison, we used a range of reference complexes including the iron corrole compound Fe[TPC]Cl, a reference complex known to exhibit significant radical character on the corrole ligand. Experimental data including temperature-dependent magnetic susceptibility measurements, magnetic Mössbauer spectroscopy, ^1^H NMR (proton nuclear magnetic resonance) spectroscopy, and theoretical calculations have firmly established Fe[TPC]Cl as an Fe^III^*S* = 3/2 intermediate-spin center antiferromagnetically coupled to the corrole radical.^21–23,44,45^ Additional reference compounds with well-defined oxidation/spin states were also included, specifically *S* = 0 low-spin d^6^ Fe^II^[TPP](ImH)_2_, *S* = 1/2 low-spin d^5^ [Fe^III^[TPP](ImH)_2_]^+^, *S* = 1 intermediate-spin d^6^ Fe^II^[Pc], and *S* = 3/2 intermediate-spin d^5^ Fe^III^[Pc]Cl. These cover the range of expected spin states for Fe[TPC](NO). Table 1 summarizes the formal Fe oxidation state, spin state as well as the Fe coordination number of these references. Following the experimental observations, we discuss the multi-configurational calculations of the core-level spectra of compounds Fe[TPC](NO) and Fe[TPC]Cl.

**Table 1:** Formal Fe coordination, oxidation state, and spin state of the reference complexes

Complex	Formal Fe properties		
	Coordination	Oxidation	Spin state
Fe^III^[Pc]Cl	5	III	3/2
Fe^II^[Pc]	4	II	1
Fe^III^[TPC]Cl	5	III	3/2
[Fe^III^[TPP](ImH)_2_]^+^	6	III	1/2
Fe^II^[TPP](ImH)_2_	6	II	0

### 3.1 Nature of the ground state from X-ray spectroscopy

Three sets of experimental spectra were recorded: Fe Kβ XES, Fe L-edge XAS, and Fe K-edge XAS. These provide a direct sensitivity to the Fe valence orbital occupancy and spin state.

#### 3.1.1 Fe Kβ XES experiment

The spectral shape of Kβ XES strongly depends on the Fe spin state^16,17,20,46^ and has been widely used to experimentally determine both the spin and oxidation states of the metal center.^47–50^ With increasing spin, characteristic features are observed: the Kβ′ region around 12 eV below the main peak (Kβ_1,3_) gains intensity, while the Kβ_1,3_ peak shifts toward higher energies. The shift of the main line Kβ_1,3_ as a function of effective spin often allows the first moment in this energy range to be a reasonable observable for the net spin of a complex.^19,20^

**Fig. 3:**
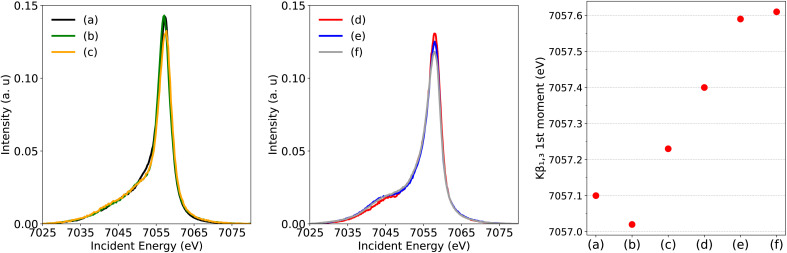
Experimental Fe Kβ XES of (a) Fe[TPC](NO) together with low-spin references (b) Fe^II^[TPP](ImH)_2_ and (c) [Fe^III^[TPP](ImH)_2_]^+^ (left figure), intermediate-spin references (d) Fe^II^[Pc], (e) Fe^III^[Pc]Cl, and (f) Fe[TPC]Cl (middle figure) and their Kβ_1,3_ first moment positions (right figure).

Fig. 3 (left) compares Fe[TPC](NO) with the two low-spin complexes Fe^II^[TPP](ImH)_2_ (*S*(Fe) = 0) and [Fe^III^[TPP](ImH)_2_]^+^ (*S*(Fe) = 1/2). All show a similar spectral shape, particularly the absence of a clear Kβ′ feature around 7045 eV. From a qualitative comparison, the absence of a visible Kβ′ feature in Fe[TPC](NO) suggests a low-spin configuration with either Fe^II^ or Fe^III^ oxidation state. The spectra of the three intermediate-spin systems Fe^II^[Pc] (*S*(Fe) = 1), Fe[Pc]Cl (*S*(Fe) = 3/2), and Fe[TPC]Cl (*S*(Fe) = 3/2) are shown in Fig. 3 (middle). All exhibit a clear Kβ′ feature. For a more quantitative analysis, the positions of the first moments of the Kβ_1,3_ region are given in Fig. 3 (right). The Fe^II^*S* = 0 low-spin reference shows the lowest Kβ_1,3_ first moment. The Fe^III^*S* = 1/2 reference complex shows a slightly higher energy position, and the two non-corrole intermediate-spin complexes, Fe^II^[Pc] (*S*(Fe) = 1) and Fe^III^[Pc]Cl (*S*(Fe) = 3/2), have their Kβ_1,3_ first moments at higher energies. The Kβ_1,3_ first moment of the Fe[TPC]Cl reference appears even slightly above that of Fe^III^[Pc]Cl, further emphasizing its Fe^III^*S* = 3/2 nature, as confirmed experimentally and theoretically previously.^22,23,44,51^ The position of the Kβ_1,3_ first moment of Fe[TPC](NO) lies between that of the two low-spin complexes Fe^II^[TPP](ImH)_2_ (*S*(Fe) = 0) and [Fe^III^[TPP](ImH)_2_]^+^ (*S*(Fe) = 1/2).

The energy position of the Kβ_1,3_ first moment of Fe low-spin compounds depends on the crystal field strength and the orbital covalency (the metal 3d character in the five metal 3d character molecular orbitals).^18,20^ The position of the Kβ_1,3_ first moment moves to lower energies with an increase in ligand field and orbital covalency. Fe[TPC](NO) has a strong π accepting NO ligand, which lowers the 3d_*xz*/*yz*_ type orbitals. These orbitals are also expected to have less metal 3d character due to the strong covalent mixing between the metal 3d_*xz*/*yz*_ type orbitals and NO π* orbitals. The contracted corrole ring can lead to strong covalent mixing between the corrole a_2u_-π and metal 3d_*z*^2^_ type orbital due to the displacement of the iron center from the corrole plane. Compared to Fe^II^[TPP](ImH)_2_, the combination of stronger π accepting and σ donating ligands leads to a stronger ligand field and more covalent orbital mixing in Fe[TPC](NO). Considering these, a lower Kβ_1,3_ first moment position than Fe^II^[TPP](ImH)_2_ (*S*(Fe) = 0) could be expected if the metal center is an Fe^II^ oxidation state, which is opposite to what is found experimentally, see Fig. 3 (right).

#### 3.1.2 Fe L-edge XAS experiment

**Fig. 4:**
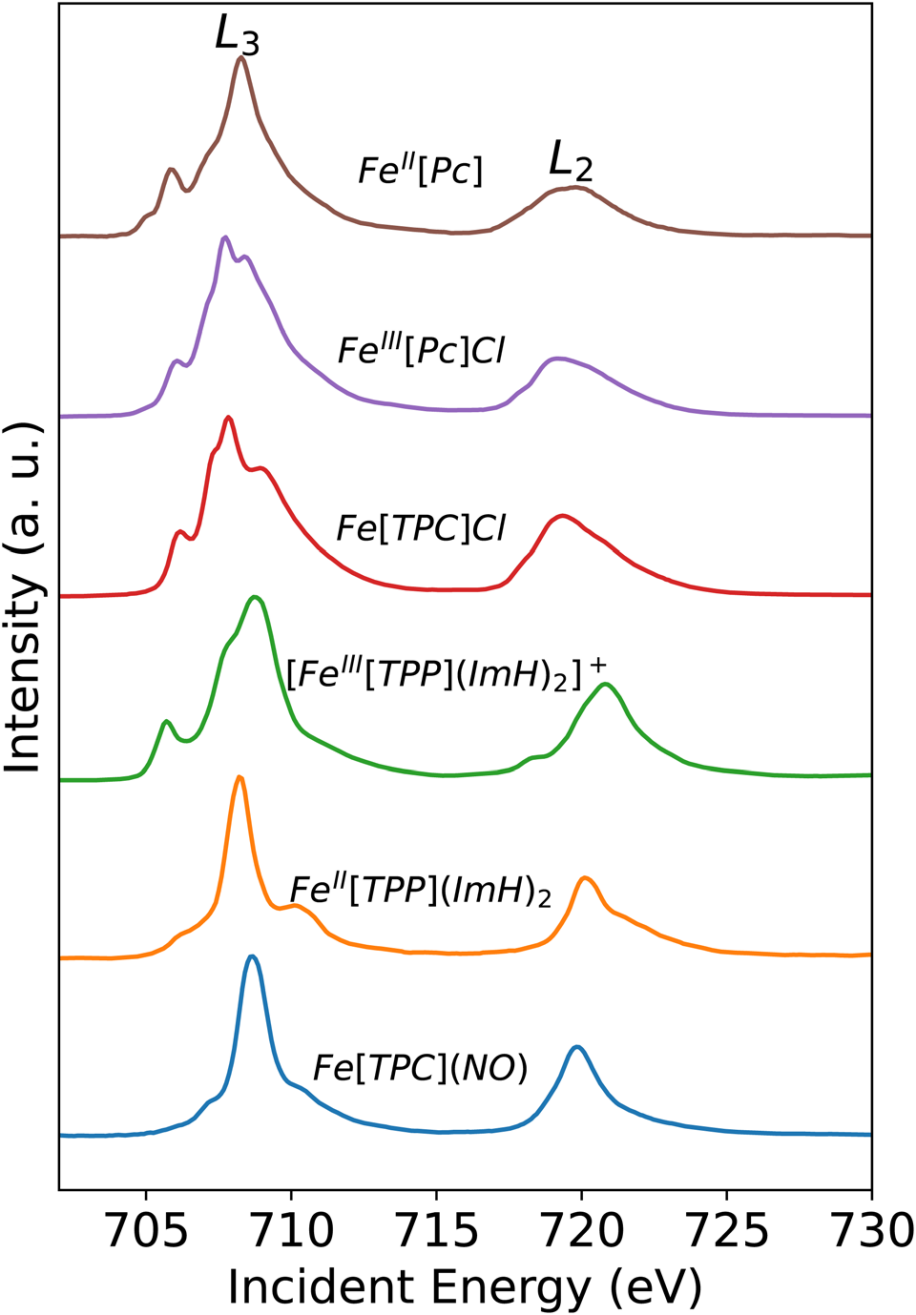
Experimental Fe L-edge XAS spectra.

Following this site-specific confirmation of a low-spin state at the Fe center from Fe Kβ XES measurement, we used metal centered XAS to further investigate the valence electronic structure and determine the oxidation state of the Fe center. In Fe L-edge XAS, an Fe 2p electron is excited into an unoccupied 3d orbital by an incoming photon with matching energy. Thus, Fe L-edge XAS probes the unoccupied (or partially occupied) valence orbitals. The Fe L-edge XAS spectrum of *S* = 0 low-spin Fe^II^[TPP](ImH)_2_ exhibits one main peak in each of the L_3_ and L_2_ regions, which is characterized as excitations into e_g_ orbitals (Fig. 4). Note that the weak feature at an energy below the main peak (at ∼706.2 eV), is due to the multiplet structure and independent of a partially filled t_2g_ shell.^27,52,53^ The Fe L-edge XAS spectrum of *S* = 1/2 low-spin [Fe^III^[TPP](ImH)_2_]^+^ consists of one main peak and a well-separated additional feature at lower energy due to the extra hole in the t_2g_ type orbitals. The oxidation state determination from L-edge XAS data often depends on the lower-energy t_2g_ character feature that has been widely used to distinguish low-spin d^5^ and d^6^ systems.^27,53–55^

From a qualitative comparison based on the spectral shape (Fig. 4), the Fe L-edge XAS spectrum of the corrole radical reference compound *S*(Fe) = 3/2 Fe[TPC]Cl exhibits very similar features to the XAS spectrum of *S*(Fe) = 3/2 intermediate-spin Fe^III^[Pc]Cl, further strengthening its Fe^III^ intermediate-spin assignment. This observation is consistent with the Kβ XES spectra. The Fe[TPC]Cl L-edge XAS is significantly different compared to the Fe[TPC](NO) spectrum. The Fe L-edge XAS spectrum of Fe[TPC](NO) agrees best with that of complex Fe^II^[TPP](ImH)_2_, which is an *S*(Fe) = 0 low-spin Fe^II^ complex.^27^ While their spectral shapes are similar, the Fe[TPC](NO) spectrum appears at ∼0.45 eV higher in energy than Fe^II^[TPP](ImH)_2_. A shift in energy between two Fe^II^ low-spin complexes has previously been explained by the difference in the crystal field strength, where a stronger crystal field leads to a higher absorption energy for excitation into the unoccupied e_g_ orbitals.^27^ An increase in oxidation state also leads to a shift towards higher energies due to differences in spin density distributions, which exhibit a shift towards the Fe nucleus in the final core-excited states compared to the initial ground states. This can be interpreted as a compression of the 3d orbitals. The shift is attributed to changes in the direct (classical) Coulomb interactions in the final states when changing the nominal oxidation state.^56^ An increase in spin at the same oxidation state would likely lead to a slightly lower absorption energy,^52^ due to differences in the crystal field strength.

However, even though the Fe L-edge XAS spectrum of Fe(*S* = 1/2) [Fe^III^[TPP](ImH)_2_]^+^ looks significantly different from that of Fe[TPC](NO), the presence of π back-bonding can still sufficiently alter the spectral shape in the direction of what is observed here. This has previously been observed in the nitrosyl complex [Fe(PaPy_3_)NO]^2+^, which was described as {FeNO}^6^ without a localized t_2g_ hole.^11^

#### 3.1.3 Fe K pre-edge XAS experiment

**Fig. 5:**
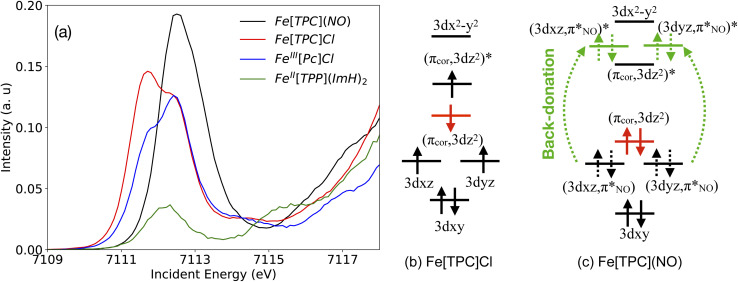
(a) Experimental Fe K pre-edge XAS spectra. Schematic representations of the orbital diagram for (b) Fe[TPC]Cl and (c) Fe[TPC](NO). For Fe[TPC](NO), the four electrons delocalized (unpolarized) into two orbital pairs are indicated with dashed arrows.

As a third set of experimental data, Fe K-edge XAS spectra were recorded. The intensity of the Fe K pre-edge XAS spectrum strongly depends on the amount of 4p character mixed into the 3d orbitals.^15,57^ In Fig. 5 the spectra of Fe[TPC](NO), Fe[TPC]Cl, Fe^III^[Pc]Cl and Fe^II^[TPP](ImH)_2_ are compared. The lack of inversion symmetry in the 5-coordinated Fe–corrole complexes and Fe^III^[Pc]Cl leads to a significantly stronger intensity in the K pre-edge XAS spectra than for the 6-coordinated Fe–porphyrin complex, which contains inversion symmetry and thus only allows for electric quadrupole excitations. The Fe[TPC]Cl and Fe^III^[Pc]Cl spectra (red abd blue) have similar K pre-edge features, except for the relative intensity of the two peaks at 7111.7 eV and 7112.5 eV, respectively (see Fig. 5(a)). In Fe[TPC]Cl and Fe^III^[Pc]Cl, the 3d_*z*^2^_ orbital can mix with the 4p_*z*_ orbital, and the 3d_*xz*/*yz*_ orbitals with the 4p_*x*,*y*_ orbitals, thus allowing 4p character in these orbitals and electric dipole transitions leading to increased intensity. The lower energy peak at ∼7111.7 eV thus arises from electron excitations to the 3d_*xz*/*yz*_ orbitals, whereas the higher energy feature at ∼7112.5 eV originates from excitations into the 3d_*z*^2^_ orbital. Both gain intensity through 4p mixing into the 3d orbitals. The main difference between the two *S* = 3/2 complexes is an increased intensity at 7111.7 eV in Fe[TPC]Cl due to stronger 4p mixing in the 3d_*xz*/*yz*_ orbitals. This is due to the contracted and more ruffled corrole ligand compared to the phthalocyanine ligand, which allows for better mixing and hence increased 4p character.^57^

The Fe K pre-edge XAS spectrum of Fe[TPC](NO) does not show intensity at the lower energy feature, pointing to a different orbital occupation of Fe 3d_*xz*/*yz*_ orbitals from those in Fe[TPC]Cl. The spectrum contains a shoulder on the higher energy side at ∼7113.0 eV, which arises from excitations into the 3d_*x*^2^−*y*^2^_ orbital and π-back-bonding orbitals as will be shown below.

On the basis of all three experimental X-ray measurements, the ground-state electronic structure of the radical reference compound Fe[TPC]Cl can be determined unambiguously in terms of spin and oxidation state (*S* = 3/2 Fe^III^), consistent with previous findings. For Fe[TPC](NO), our experimental data support the presence of a low-spin iron center, although the oxidation state remains uncertain. The low-spin assignment stands in clear contrast to recent theoretical studies employing high-level multiconfigurational theory such as CASPT2 and DMRG.^9,58^ A more definitive determination of the oxidation state will require further theoretical efforts capable of directly bridging experimental X-ray spectral features with electronic structure information, in particular through explicit calculations of the X-ray spectra.

### 3.2 Results from muticonfigurational calculations

The uncertainty in the electronic structure (spin and oxidation state) of the Fe ion arises from the character of the corrole and NO ligands, which are (partially) known as non-innocent ligands in bioinorganic systems.^59–61^ The non-innocence is mainly due to the strong covalent orbital mixing between the metal and the ligand, allowing electron delocalization between the metal 3d orbitals and the ligand character orbitals. The Fe 3d orbitals mix strongly with the corrole ligand, which is a “ring-contracted” porphyrin with strong σ-donation in the equatorial coordination core and electron richness in the macrocyclic π-system. The NO ligand, with its π-accepting capability, can also interact strongly with the iron center. These two-way charge-transfer interactions in Fe[TPC](NO) make the determination of the electronic structure challenging. Multiconfigurational calculations are particularly well-suited for unraveling the electronic structure of these systems due to the multi-determinant nature of the method, which allows for the inclusion of multiple configurations in the wavefunction.

Here, we first compare the experimental X-ray absorption spectra (XAS) of both the K-pre-edge and L-edge, with those calculated using a multiconfigurational approach. The good agreement between experimental and theoretical spectra then enables a more detailed analysis of the ground-state electronic structure through spectral decomposition, and ground-state calculations using the same valence orbital active space as the XAS calculations. These efforts effectively bridge experimental measurements with the underlying molecular electronic structure.

#### 3.2.1 Calculation of X-ray absorption spectra

##### 3.2.1.1 Fe K pre-edge XAS calculations

**Fig. 6:**
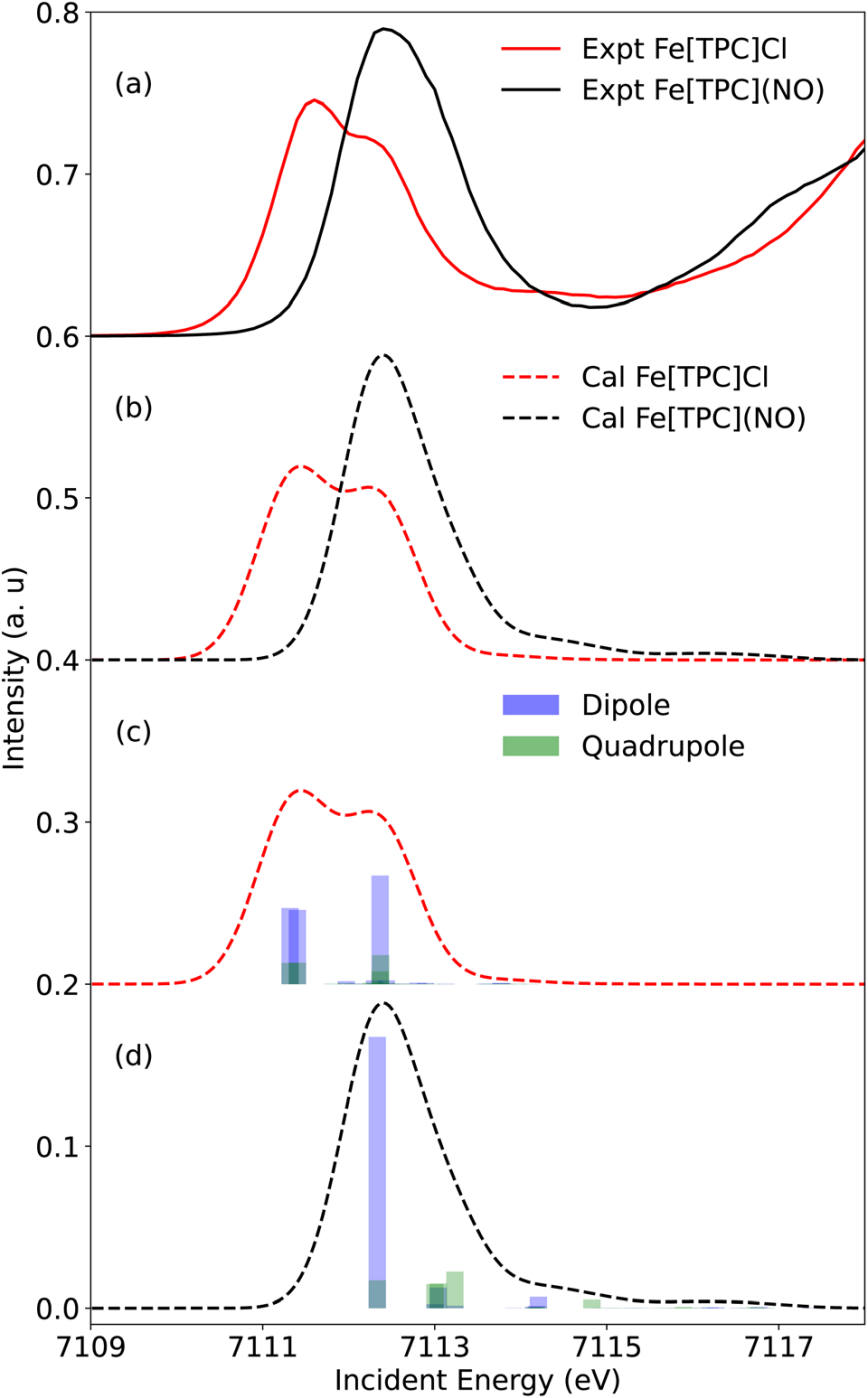
The (a) experimental Fe K pre-edge XAS and (b) calculated XAS of Fe[TPC]Cl (red) and Fe[TPC](NO) (black). The same scaling factor and shift energy are applied for both calculated spectra. The electric dipole (blue sticks) and quadrupole (green sticks) contributions to the calculated Fe K pre-edge are presented for (c) Fe[TPC]Cl and (d) Fe[TPC](NO).

As mentioned in the experimental results, the Fe K pre-edge XAS features are dominated by electric dipole transitions in 5-coordinated systems due to the absence of inversion symmetry. The information about the metal 3d character molecular orbitals is hence obscured and access to the electronic structure from the Fe K-pre-edge is limited and challenging. The experimental and RAS calculated Fe K-pre-edge XAS spectra of Fe[TPC]Cl (red) and Fe[TPC](NO) (black curve) are presented in Fig. 6(a) and (b), respectively. The calculated spectra nicely reproduce the experimental features in terms of peak intensities and positions.

**Fig. 7:**
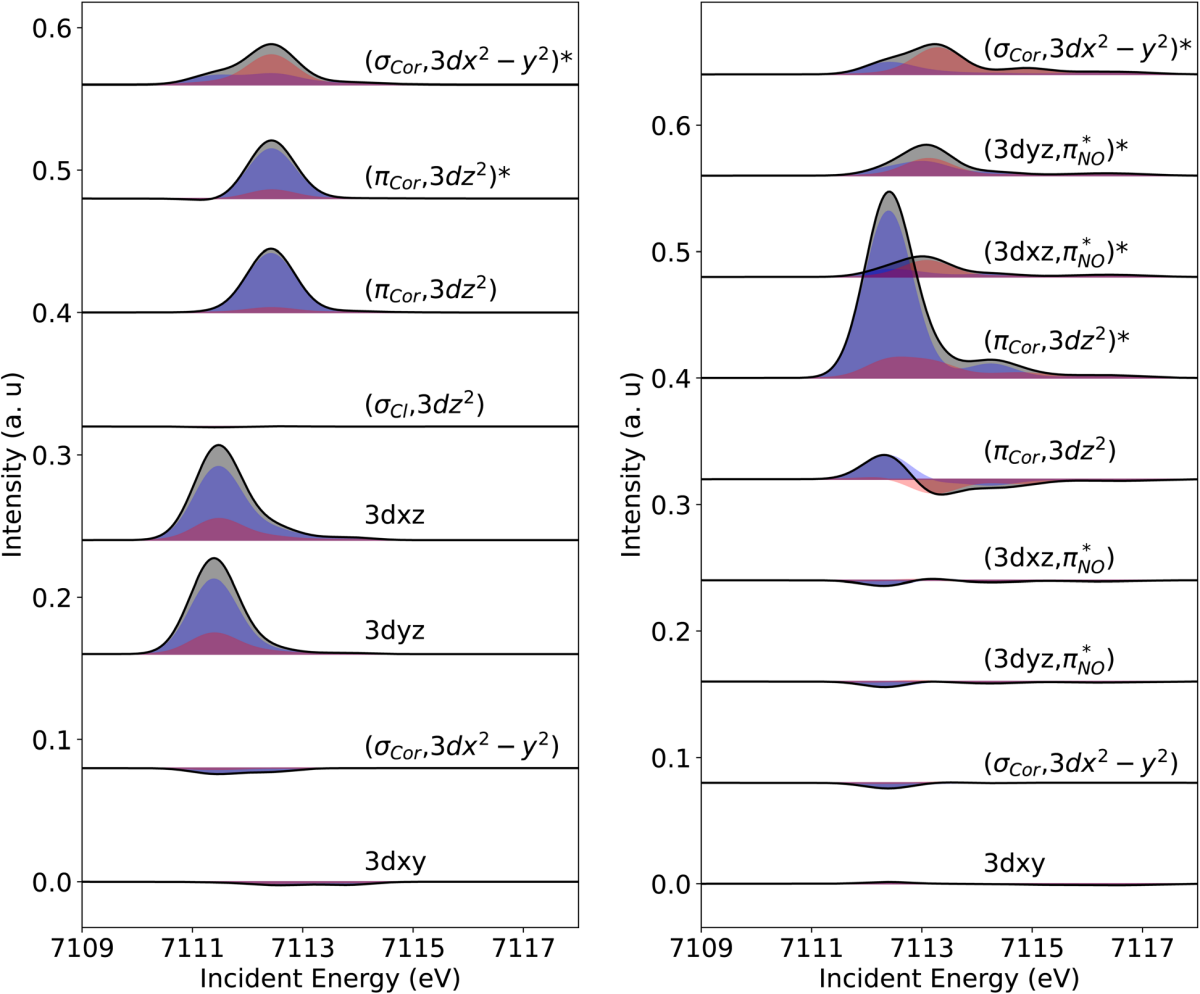
Electric dipole (blue) and quadrupole (red) transition contributions to the calculated Fe K pre-edge XAS of Fe[TPC]Cl (left) and Fe[TPC](NO) (right).

For a deeper analysis of the spectral origins, an orbital contribution analysis was performed. Orbital contributions are derived from the difference in orbital occupation between the ground and core-excited states, correlated with the transition oscillator strengths. Consequently, both positive (electron gain) and negative (electron loss) “intensities” are observed.^62^ This is shown in Fig. 7 in the orbital contributions analysis for Fe[TPC]Cl (left) and Fe[TPC](NO) (right).

Starting with Fe[TPC]Cl and following the orbital contribution analysis, the intensity of the first peak at around 7111.7 eV originates from excitations to the singly occupied 3d_*xz*_ and 3d_*yz*_ metal character orbitals, with metal 4p_*x*,*y*_ character mixed into these orbitals, which leads to a strong electric dipole contribution (Fig. 6(c)). The strong intensity at 7112.5 eV arises from excitations into both the (π_cor_, 3d_*z*^2^_) and (π_cor_, 3d_*z*^2^_)* orbitals that allow for electric dipole transitions through mixing of 4p_*z*_ into these orbitals. This feature overlaps with transition to the 3d_*x*^2^−*y*^2^_ orbital. Since an electric dipole transition is around 100 times stronger than an electric quadrupole transition, a small amount of 4p character mixed into the 3d orbital can dramatically change the intensity distribution. To gain a deeper insight into the Fe 3d character molecular orbitals, a separate orbital contribution analysis was performed considering only electric quadrupole transitions, please see Fig. S3 in the SI. The electric quadruple intensities of the 3d_*xz*_ and 3d_*yz*_ orbitals show comparable integrated areas, with both gaining a similar amount of electron density during the transitions (Fig. 7 and S3), highlighting their similar occupations. Likewise, the intensities associated with excitations to the (π_cor_, 3d_*z*^2^_) and (π_cor_, 3d_*z*^2^_)* orbitals are also comparable. Moreover, the sum of electric quadrupole intensity of the (π_cor_, 3d_*z*^2^_) and (π_cor_, 3d_*z*^2^_)* orbitals is close to that of the 3d_*xz*_/3d_*yz*_, indicating that the 3d_*z*^2^_ character is evenly distributed between the (π_cor_, 3d_*z*^2^_) and (π_cor_, 3d_*z*^2^_)* orbitals due to strong covalent mixing. Taken together, all these observations point to similar occupations in these orbitals and support an electronic structure best described as Fe^III^*S* = 3/2 antiferromagnetically coupled to corrolė^2−^, see the orbital diagram in Fig. 5(b).

For Fe[TPC](NO), a different intensity distribution is found. Here, the spectrum is dominated by an electric dipole transition at 7112.5 eV (Fig. 6(d) and 7). This transition arises from the excitation of a 1s electron into a metal 3d_*z*^2^_ character orbital with Fe 4p_*z*_ character mixed into it, allowing for the strong electric dipole intensity. The transition at 7113.2 eV corresponds to excitations into metal 3d_*x*^2^−*y*^2^_, through electric quadrupole contributions. Transitions to the π back-donation ligand character orbitals, specifically 
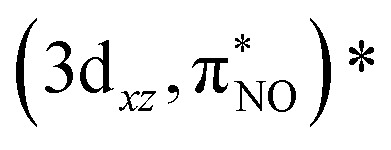
 and 
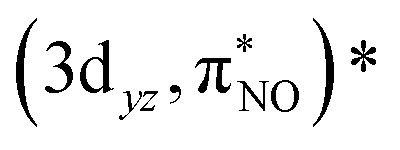
, occur at around 7113.1 eV. The back-donation transitions overlap with the transitions to the metal 3d_*x*^2^−*y*^2^_ character orbital. The π* peak shifts to lower energy and merges with the metal character peak. This observation is consistent with the potential π* peak assignment in the experimental L-edge XAS spectrum (*vide infra*). From the analysis of the quadrupole transition intensity in the orbital contribution plot (Fig. S3), we find that transitions to the 3d_*x*^2^−*y*^2^_ and (π_cor_, 3d_*z*^2^_)* orbitals carry major intensity. Significant intensity is also observed for the back-donation charge transfer transitions, which are closely located in energy to the transitions to the (σ_cor_, 3d_*x*^2^−*y*^2^_)* and (π_cor_, 3d_*z*^2^_)* orbitals. Also, the 
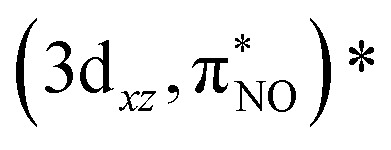
 and 
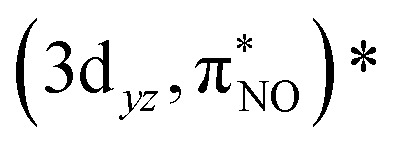
 contribute considerable and nearly identical electric quadrupole intensities, indicating an unpolarized distribution of Fe 3d character. In addition, there is substantial electron loss from the (π_cor_, 3d_*z*^2^_) orbital, while a minor electron gain is observed at around 7112 eV, underscoring the partial but nearly double occupation of this orbital. The energy levels and electron gain behavior of the 
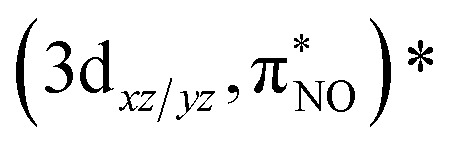
 orbitals, together with the comparable quadrupole intensities of (π_cor_, 3d_*z*^2^_)* and (σ_cor_, 3d_*x*^2^−*y*^2^_)* (the latter known to be completely unoccupied), as well as the substantial electron loss from the (π_cor_, 3d_*z*^2^_) orbital, suggest that Fe[TPC](NO) is best described as {FeNO}^6^ with a corrole^3−^ ligand. The {FeNO}^6^ unit corresponds to a Fe^III^ center without a localized hole in the iron π t_2g_ orbital. (*i.e.* no dπ hole in the L edge spectrum of Fe[TPC](NO) in Fig. 4), due to strong covalent mixing between the iron dπ and NO π* orbitals, see the orbital diagram in Fig. 5(c).

##### 3.2.1.2 Fe L-edge XAS calculations

**Fig. 8:**
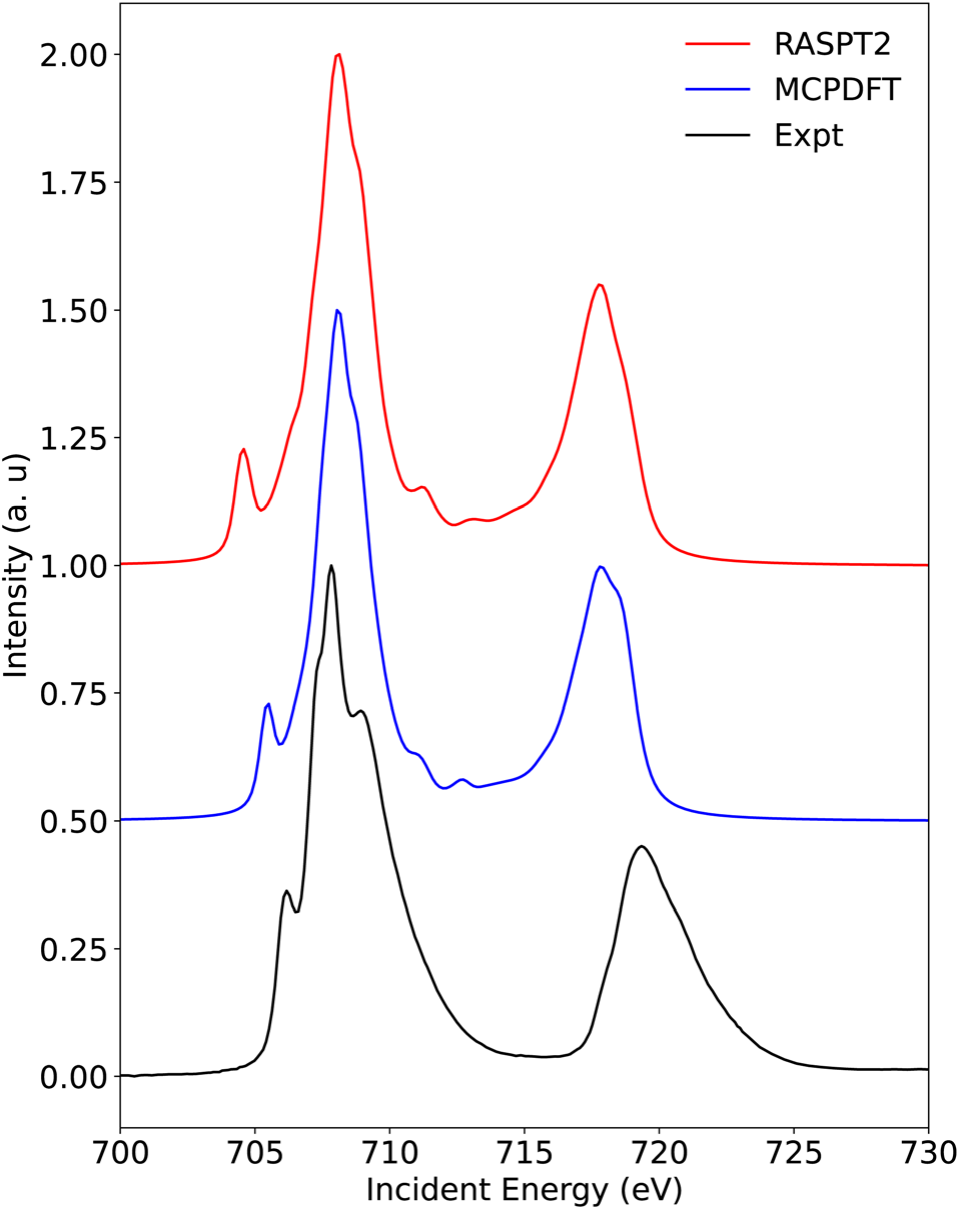
The experimental Fe L-edge XAS of Fe[TPC]Cl and the calculated Fe L-edge XAS at the RASPT2 level and the multiconfiguration pair-density functional theory (MCPDFT) level.

**Fig. 9:**
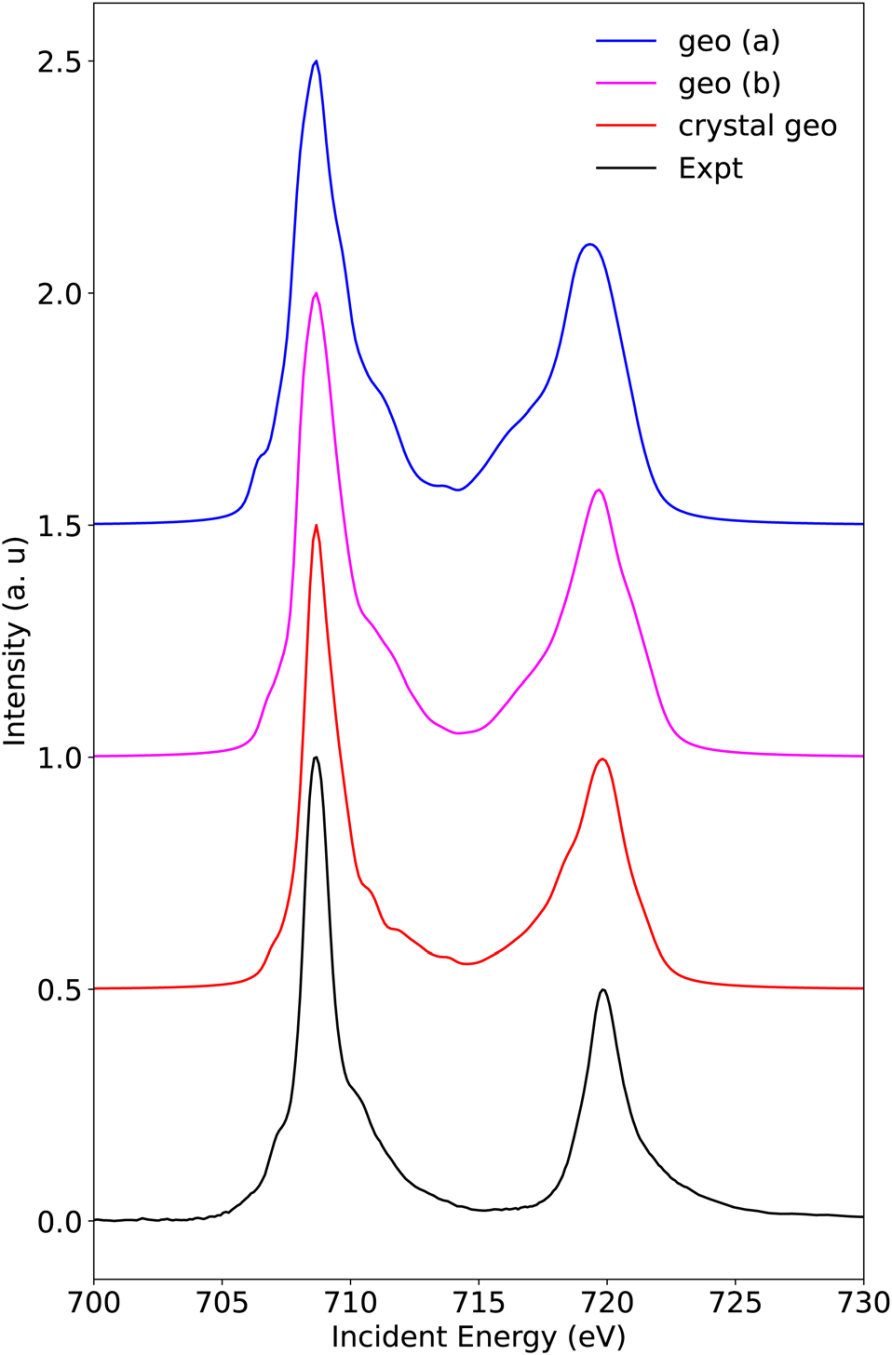
The experimental Fe L-edge XAS of Fe[TPC](NO) and calculated XAS with different geometries, (a) structure from DFT closed-shell singlet optimization, (b) structure from broken-symmetry DFT open-shell singlet optimization, and crystal structure from ref. 68.

The calculated and experimental spectra for Fe[TPC]Cl and Fe[TPC](NO) are shown in Fig. 8 and 9. Here, we will focus on the L_3_ edge, because it is better resolved compared to the L_2_ edge due to the difference in the lifetime width.^63–65^ Additionally, the L_2_ edge features locate approximately 12 eV higher in energy relative to the L_3_ edge, and thus a large number of core-excited states are necessary to fully capture its experimental features, which poses significant challenges for medium-sized systems lacking evident symmetry. Moreover, spin–orbit coupling is treated within the atomic mean-field approximation and applied after the spin-free RAS calculations in the present work.^43^ This spin–orbit coupling treatment as well as the description of core-hole correlation and relaxation within the finite configuration space leads to a slight underestimation of L_3_/L_2_ edge splitting. This underestimation is a known limitation of RAS method based L-edge calculations and has been noted in previous applications of this method to other transition-metal compounds.^54,62,66,67^

The RASPT2-calculated Fe L-edge XAS spectrum of Fe[TPC]Cl reproduces the experimental features well, except for the position of the lower energy peak at the L_3_ edge (Fig. 8). This discrepancy can be improved using the multiconfigurational pair density functional theory (MCPDFT) method, which highlights the impact of dynamic correlation effects on the L-edge calculations. Dynamic correlation corrections in RASPT2 depend on the size of the active space, whereas MCPDFT shows relatively less dependence on this factor.^41^ The validity of the MCPDFT method for modeling XAS of transition metal complexes has also been demonstrated recently.^69^ Two crystal geometries for Fe[TPC]Cl were published^45,70^ that lead to very similar calculated Fe L-edge XAS spectra (see Fig. S4), emphasizing the robustness of the method used and the consequent results.

The analysis of the metal L_3_ edge XAS in terms of orbital contributions is shown in Fig. S5 and S6. Orbital contribution analysis reveals an intermediate-spin iron center for Fe[TPC]Cl, as indicated by the individual orbital contributions. Specifically, the 3d_*xz*_ and 3d_*yz*_ orbitals have similar integrated areas, confirming that both are singly occupied. Using this as a reference for a singly occupied orbital, the analysis also confirms that the (π_cor_, 3d_*z*^2^_)* orbital is nearly singly occupied. Combined with the known doubly occupied 3d_*xy*_ orbital and the completely empty (σ_cor_, 3d_*x*^2^−*y*^2^_)* orbital, these observations confirm the presence of an S(Fe) = 3/2 intermediate-spin Fe^III^, see Fig. 5(b).

The RAS calculated L-edge XAS spectra of Fe[TPC](NO) (Fig. 9) nicely reproduce the experimental features for three sets of geometric structures: (a) structure from closed-shell DFT singlet optimization (B3LYP functional), (b) structure from broken-symmetry DFT open-shell singlet optimization (B3LYP), and (c) the crystal structure.^68^ Despite the generally good agreement, subtle differences are observable, particularly in the width of the L_3_-edge. The best agreement is found for the calculation using the crystal structure, while the two DFT optimized structures show a broader main peak with additional features. Note that the DFT optimized structures predict the Fe–NO bond distance to be 0.1 Å longer than the experimentally determined value.^68^

Orbital contribution analysis of the L_3_ edge XAS of Fe[TPC](NO) is shown in Fig. S7 and S8. For Fe[TPC](NO), the orbital contribution analysis reveals that the metal-character orbital (π_cor_, 3d_*z*^2^_)* gains electron density similar to that of the completely empty orbital (σ_cor_, 3d_*x*^2^−*y*^2^_)*. Meanwhile, the (π_cor_, 3d_*z*^2^_) orbital exhibits significant negative intensity, similar to the doubly occupied 3d_*xy*_ metal-character orbital. The 
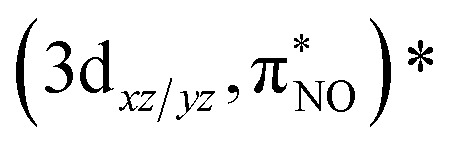
 orbitals gain considerable intensity, consistent with strong back-donation charge transfer as we observed in the K pre-edge XAS. Correspondingly, electron loss is observed in their pairwise bonding orbitals 
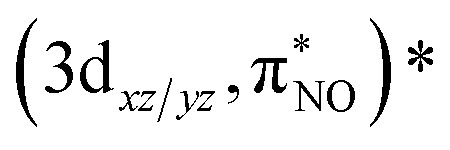
, with similar losses for both orbitals. The metal-to-ligand charge transfer (MLCT) features closely align with the transitions to the (π_cor_, 3d_*z*^2^_)* and (σ_cor_, 3d_*x*^2^−*y*^2^_)* orbitals, rendering them invisible in experimental L-edge XAS due to merging of the double peak feature (e_g_ and back-donation MLCT peaks). These observations are also consistent with the Fe K pre-edge XAS and collectively support the electronic configuration illustrated in Fig. 5(c).

It is worth noting that the orbital contribution analysis for metal L-edge XAS can obscure the origin of certain spectral features, particularly those arising from multiple excitations (including charge-transfer contributions) and from the extensive mixing of core-excited states induced by strong 2p spin–orbit coupling. Isolating these effects is intrinsically challenging and, in principle, could be addressed through careful active-space design or by employing the generalized active space (GAS) methodology,^71^ which would allow individual excitation classes to be evaluated explicitly. However, such an approach is beyond the scope of the present study. Similarly, although performing XAS calculations without spin–orbit coupling could aid in disentangling these effects, this is not practical for L-edge XAS, where spin–orbit coupling is an essential component of the spectroscopy.

To obtain a more detailed and chemically intuitive picture of the orbital contributions, two alternative strategies were considered. The first is to examine the Fe K pre-edge XAS, as discussed above, where spin–orbit effects are negligible and state mixing is significantly reduced. The second approach is to decompose the L-edge XAS into contributions from core-excited states of different spin multiplicities. In this framework, orbital contribution analyses can be performed for transitions involving specific spin changes, such as Δ*S* = 0, Δ*S* = 1, and Δ*S* = 2, as illustrated in Fig. S5 to S8. The orbital contributions associated with the different Δ*S* channels exhibit broadly similar characteristics, leading to conclusions consistent with those discussed above.

#### 3.2.2 Multiconfigurational character of the ground state

To reference the radical character in the corrole ligand, we first analyze the ground-state wave function of compound Fe[TPC]Cl. The ground-state wavefunction shows a dominant configuration 
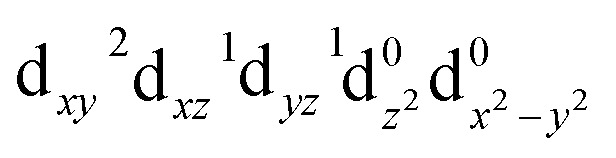
 (∼60%), which contains two unpaired electrons on the iron center, and seems to support a ferryl Fe^IV^ assignment (see configuration 1 in Table 2). Our Fe Kβ XES and L-edge XAS data support an *S* = 3/2 intermediate-spin Fe^III^ center, which is in agreement with experimental data including temperature-dependent magnetic susceptibility measurements, magnetic Mössbauer spectroscopy, ^1^H NMR spectroscopy and theoretical calculations, confirming an Fe^III^*S* = 3/2 intermediate-spin center antiferromagnetically coupled to the radical on the corrole.^21–23,44,45^ The polarization can be accounted for by including configurations that contain excitations from the (π_cor_, 3d_*z*^2^_) bonding orbital to the (π_cor_, 3d_*z*^2^_)* anti-bonding orbital (see configurations 2–4 in Table 2). These redistribute the electron density between the metal center and corrole ligand, resulting in an *S* = 3/2 Fe^III^ center antiferromagnetically coupled to an *S* = −1/2 corrolė^2−^ radical. The ground state calculations on crystal structures from two references gave very similar results, as seen in Table 2.

**Table 2:** The most important configuration contributions to the ground state wavefunction of Fe[TPC]Cl from CASSCF calculations with different crystal geometries. Four orbitals of interest are selected from the active space shown in Fig. S2; the other active orbitals are nearly doubly occupied or empty in the dominant configurations. The weights (square of the CI-coefficient) are given. Crystal geo (a) is taken from ref. 45; crystal geo (b) is taken from ref. 70. 2: doubly occupied orbital, u and d: spin up and down electron, and 0: empty orbital

Index	Configurations	Crystal geo (a) weights	Crystal geo (b) weights
1	[d_*yz*_]^u^[d_*xz*_]^u^[π_cor_, d_*z*^2^_]^2^[(π_cor_, d_*z*^2^_)*]^0^	0.62	0.63
2	[d_*yz*_]^u^[d_*xz*_]^u^[π_cor_, d_*z*^2^_]^0^[(π_cor_, d_*z*^2^_)*]^2^	0.16	0.16
3	[d_*yz*_]^u^[d_*xz*_]^u^[π_cor_, d_*z*^2^_]^u^[(π_cor_, d_*z*^2^_)*]^d^	0.12	0.06
4	[d_*yz*_]^u^[d_*xz*_]^u^[π_cor_, d_*z*^2^_]^d^[(π_cor_, d_*z*^2^_)*]^u^	0.06	0.12

For Fe[TPC](NO), the ground-state calculations with two different active spaces CAS(10;9) and CAS(10;14) (with the 4d character orbitals) give very similar orbital occupation numbers. These results are also very close to previous multiconfigurational and density matrix renormalization group (DMRG) calculations using a larger active space.^9,58^ The ground-state calculations indicate that the 3d_*xy*_ orbital is doubly occupied and the 3d_*x*^2^−*y*^2^_ orbital is completely empty.

In Table 3, the most important configurations contributing to the ground-state wavefunction from the CASSCF calculations with different geometries are given. The first geometry (crystal geo) uses the crystal structure,^68^ and the second and third are DFT optimized structures using the B3LYP functional (B3LYP geo) obtained from either a closed-shell (a) or broken-symmetry singlet calculation (b), respectively. Note that the B3LYP optimized structures overestimate the Fe–NO bond distance by around 0.1 Å. The ground-state wavefunction characters are similar across all geometries with one dominant configuration contributing to the ground state wavefunction. The dominant configuration (index 1) is a closed-shell configuration with doubly occupied 3d_*xy*_, 
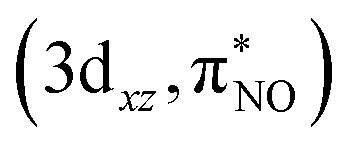
, and 
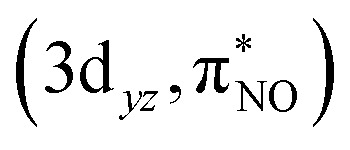
 metal character orbitals. Additionally, there are three configurations (indices 2–4) that correspond to excitations from the bonding orbitals (
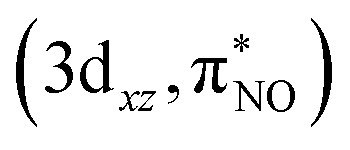
 and 
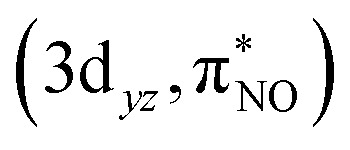
) to the antibonding orbitals (
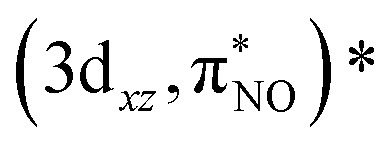
 and 
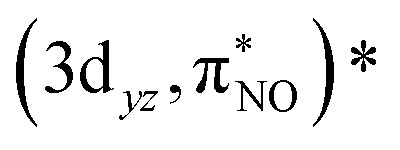
), and also from (π_cor_, 3d_*z*^2^_) to (π_cor_, 3d_*z*^2^_)*. It is noted that the configuration involving electron excitation from (π_cor_, 3d_*z*^2^_) to (π_cor_, 3d_*z*^2^_)* contributes much less compared to that in Fe[TPC]Cl, which means that a smaller amount of radical character is present on the corrole ligand in Fe[TPC](NO) than in Fe[TPC]Cl. These three configurations are also closed-shell with an electron density redistribution among the bonding and anti-bonding orbitals. There are also some contributions from electron excitations of either α or β, which result in electron density redistribution. In configuration 5, one β electron is excited from (
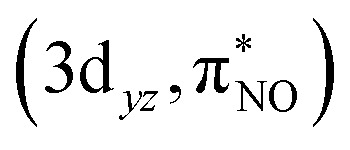
 to 
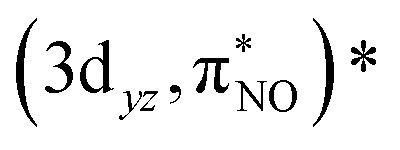
, and one α electron is excited from 
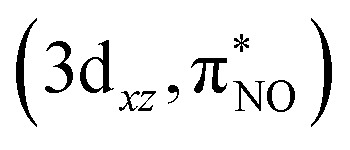
 to 
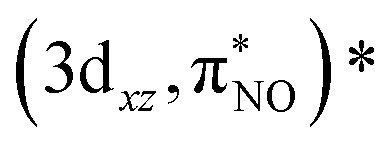
. In configurations 6 and 7, there is one electron excitation from the ligand character orbital (π_cor_, 3d_*z*^2^_) to the metal character orbital (π_cor_, 3d_*z*^2^_)*, together with one electron excitation from a bonding orbital (
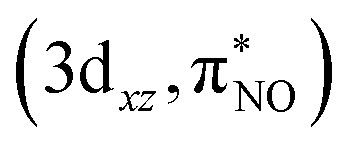
 or 
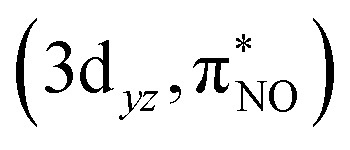
) to an antibonding orbital (
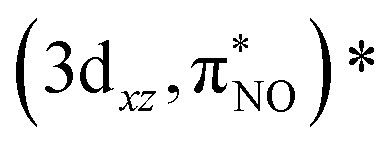
 or 
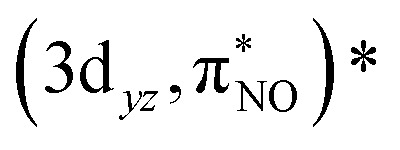
). These excited configurations lead to electron density redistribution among the metal center, corrole ligand, and NO ligand, and contribute to the radical character. Differences in the excited-state determinants among the three structures were also reflected in the calculated L-edge XAS (see Fig. 9), highlighting both the sensitivity of the L-edge XAS technique and the accuracy of the RAS method.

**Table 3:** The most important configuration contributions to the ground state wavefunction of Fe[TPC](NO) from CASSCF calculations with different geometries. B3LYP-geo (a) is obtained from closed-shell singlet optimization with the B3LYP functional. B3LYP-geo (b) is obtained from broken-symmetry singlet optimization with the B3LYP functional. Six orbitals of interest are selected from the active space in Fig. 2; the other active orbitals are nearly doubly occupied or empty in the dominant configurations

Index	Configurations	Crystal geo weights	B3LYP geo (a) weights	B3LYP geo (b) weights
1	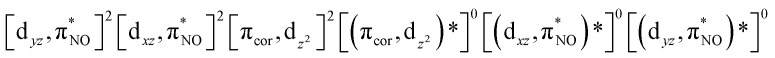	0.70	0.62	0.65
2	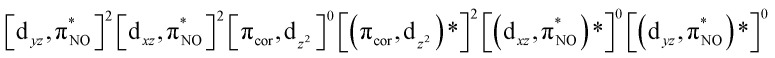	0.05	0.08	0.06
3	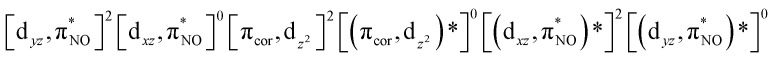	0.04	0.05	0.04
4	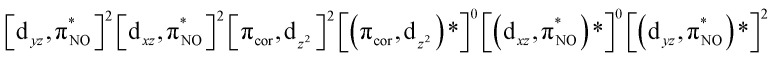	0.04	0.04	0.04
5	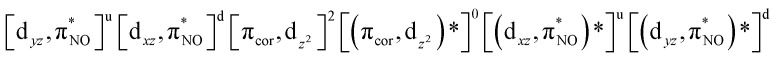	0.03	0.04	0.03
6	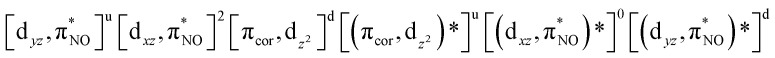	0.02	0.03	0.03
7	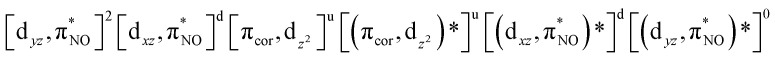	0.01	0.02	0.01

Notably, CASCI analysis with localized orbitals obtained from CASSCF or DMRG calculations indicates significant radical character on the corrole ligand in both Fe[TPC]Cl and Fe[TPC](NO).^9,22,58,72^ We implemented the same analysis and reproduced the published results, as shown in Table 4. The representative localized active orbitals used for CASCI are available in the SI, see Fig. S9 and S10. Owing to the strong covalent mixing between the corrole π-character orbital [π_cor_, d_*z*^2^_]^*m*^ and the Fe 3d-character orbital [(π_cor_, d_*z*^2^_)*]^*n*^, the ground-state wavefunction can be decomposed into configurations with *m* = 0, 1, 2. The dominant contributions of these configurations are summarized in Tables 2 and 3. Importantly, the radical character localized on the corrole ligand is directly correlated with these electronic configurations. It can be quantified either by the cumulative weight of the *m* = 1 configurations or, equivalently, by the summed weight of the configurations with *n* ≠ 0 and *m* + *n* = 2, which correspond to [Cor]˙^2−^ based electronic structures.

**Table 4:** Weights of dominant configurations based on [Cor]˙^2−^ for Fe[TPC]Cl and Fe[TPC](NO) from CASCI and CASSCF ground-state calculations

Fe[TPC]Cl		Fe[TPC](NO)	
Active space	Weight	Active space	Weight
CASCI(4;4)	0.95	CASCI(6;6)	0.78
CASCI(10;8)	0.89	CASCI(10;9)	0.74
CASCI(10;13)	0.88	CASCI(10;14)	0.73
CASSCF(10;8)	0.34	CASSCF(10;9)	0.05–0.10
CASSCF(10;13)	0.33	CASSCF(10;14)	0.05–0.10

A striking difference emerges between the weights of the dominant configurations obtained from the CASSCF and CASCI results. For Fe[TPC]Cl and Fe[TPC](NO), the dominant configurations based on [Cor]˙^2−^ from CASSCF differ significantly. The weight is small for Fe[TPC](NO), while a significant contribution is found for Fe[TPC]Cl, as shown in Table 4. However, the ground-state electronic structure determined from the CASCI analysis of Fe[TPC](NO) is clearly inconsistent with the experimental data from both XAS and Kβ XES, which offers a more direct insight into the Fe ground-state electronic structure. In contrast, the CASCI-based ground-state electronic structure for Fe[TPC]Cl aligns well with the experimental X-ray data.

Comparison of the CASCI results with X-ray data shows that, although CASCI is widely used, it cannot independently determine the ground-state electronic structure. The determinants generated from CASCI with localized orbitals may not be well-suited to capture the correlation or delocalization that the system physically favors since the orbitals are frozen. Crucially, because the CASCI wavefunction with localized orbitals does not directly correlate with observable properties, great care must be taken when using this approach to determine the electronic structure of highly covalent systems. Instead, establishing a direct connection to the ground-state wavefunction obtained from multiconfigurational calculations requires the calculation of molecular X-ray spectra at the same level of theory. As demonstrated above, visualizing the ground-state wavefunction through X-ray spectroscopy provides a rigorous means to assess the validity of the wavefunction analysis and thus enables an unbiased assignment of the ground-state electronic structure.

## 4 Discussion

Iron nitrosyl porphyrins and heme proteins have long served as a classical framework for understanding the interaction of nitric oxide with transition metal centers. In these systems, the {FeNO}^7^ electronic structure is generally described as resonance hybrids between Fe^II^–NO(neutral) and Fe^III^–NO^−^. The ground state is usually a doublet with low-lying quartet states that play key roles in ligand binding and dissociation.^6,73–77^ The sensitivity of this balance to axial coordination is well established: strong π-acceptors stabilize low-spin electronic structures, while weaker donors or neutral ligands shift spin-state energetics.

Against this benchmark, Fe corrole nitrosyls offer both similarities and distinct deviations. The contracted, trianionic corrole ligand strengthens the equatorial ligand field relative to porphyrins, enhancing Fe–ligand orbital interactions and amplifying the effect of the axial ligand. As shown here, Fe[TPC](NO) retains a {FeNO}^6^ description with strong π back-bonding, corresponding to a low-spin Fe^III^ center, but the corrole does not exhibit the pronounced radical character found in Fe[TPC]Cl. This reflects a subtle balance between the axial ligand and the equatorial electron-rich corrole framework. In Fe[TPC](NO), two potentially non-innocent ligands coexist: the corrole ligand, a “ring-contracted” porphyrin with strong σ-donation and an electron rich macrocyclic π-system, and the NO ligand accepting electron density from the metal through π back-donation. The interplay of electron donation and acceptance between these two ligands presents challenges in assigning the electronic structure of the iron center. To disentangle these effects, we employed two types of model simulations.

(i) The first model isolates the [FeNO] unit from the Fe[TPC](NO) molecule, embedding it in an environment represented by four point charges positioned at the nitrogen coordinates of Fe[TPC](NO). This arrangement approximates a square-planar crystal field, and reproduces the orbital occupations that are present in the full Fe[TPC](NO) complex. The [FeNO] unit was studied in two charge/spin states: (a) [FeNO]^3+^ ({FeNO}^6^, singlet state), representing the limiting case of an innocent corrole^3−^, and (b) [FeNO]^2+^ ({FeNO}^7^, doublet state), representing a non-innocent corrolė^2−^ scenario.

Calculations for [FeNO]^3+^ reveal that the low lying orbitals 3d_*xy*_, 
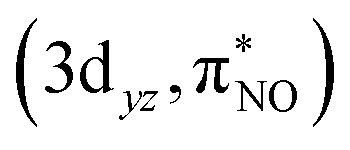
, and 
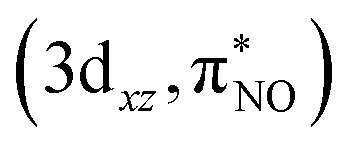
 are fully occupied in the dominant configuration, in agreement with the Fe[TPC](NO) complex (see Table 1 and Fig. S11 to S18 in the SI). In contrast, the calculations for [FeNO]^2+^ reveal a singly occupied 3d_*z*^2^_ metal-character orbital in the dominant configuration, consistent with the [Fe[TPC](NO)]^−^ anion (doublet, {FeNO}^7^). In the molecular system, this additional electron in the 3d_*z*^2^_ metal orbital arises from the orbital interaction between the Fe 3d_*z*^2^_ and corrole π orbitals, making 3d_*z*^2^_ occupation a direct probe of the corrole radical character.

As further validation, we also calculated the Fe K pre-edge XAS spectrum of the [FeNO]^3+^ unit. The resulting spectrum closely resembles that of the molecular complex, but with reduced intensity (Fig. S19). This reduced intensity reflects the absence of electric dipole transitions into the 3d_*z*^2^_ orbital, which in the full molecular system interacts with the ligand corrole π orbital. This limitation reduces mixing of metal 4p character into the 3d_*z*^2^_ orbital. These observations further support that Fe[TPC](NO) possesses a {FeNO}^6^ electronic structure together with a corrole^3−^ ligand.

(ii) The second model uses the Fe[TPC](NO) molecule but with varied Fe–NO bond lengths. Ground-state calculations were performed by varying the Fe–NO bond length Δ(Fe–NO) from −0.20 Å to 0.50 Å relative to the ground-state, while keeping the corrole geometry unchanged. The orbital occupations obtained from the active space CAS (10;9) and the relative energy difference between the lowest singlet and triplet state within a small active space of two active orbitals and two electrons ([2o,2e], specifically (π_cor_, 3d_*z*^2^_) and (π_cor_, 3d_*z*^2^_)* orbitals) were examined as a function of Fe–NO bond length. Here, the triplet state is used to evaluate the orbital splitting between (π_cor_, 3d_*z*^2^_) and (π_cor_, 3d_*z*^2^_)* orbitals. This approach is also appropriate for estimating the contribution to the radical character by treating the triplet state as the excited-state determinant contribution to the wavefunction of the molecular system. With these calculations, two properties were analyzed: (a) the change in occupation number of the active orbitals from CAS(10;9) upon varying the Fe–NO bond distance and (b) the energy gap between the (π_cor_, 3d_*z*^2^_) and (π_cor_, 3d_*z*^2^_)* orbitals.

**Fig. 10:**
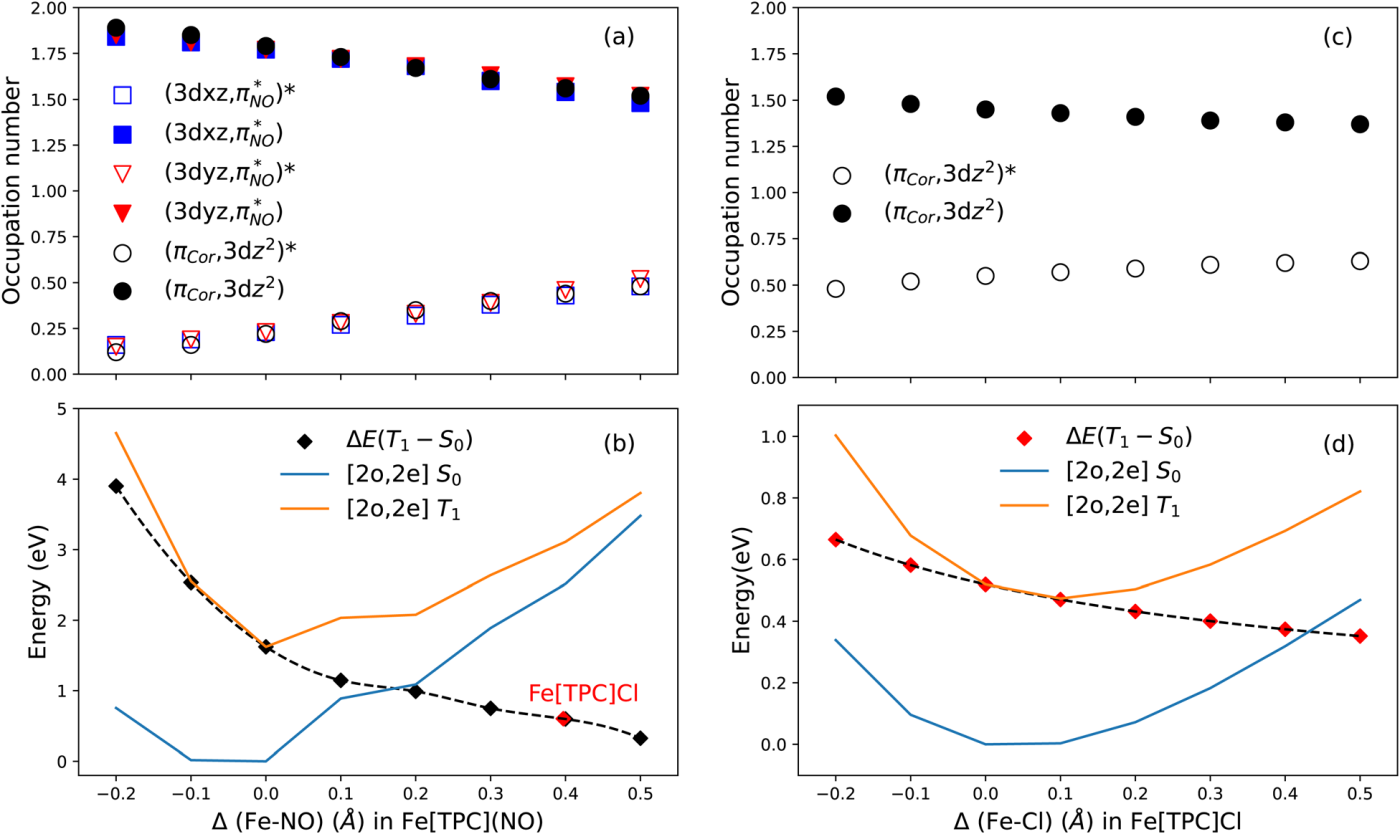
The orbital occupation numbers of selected key orbitals along the varied Fe–NO (a) and Fe–Cl (c) bond lengths. Energies of singlet (blue) and triplet (orange) states with a [2o,2e] model for varied Fe–NO (b) and Fe–Cl (d) bond lengths relative to the undisturbed ground state. The black dashed line in (b) and (d) indicates the energy difference between the singlet and triplet states.

For (a), the occupation numbers of the orbitals 3d_*xy*_, (σ_cor_, 3d_*x*^2^−*y*^2^_) and (σ_cor_, 3d_*x*^2^−*y*^2^_)* change negligibly, while the occupation numbers of the six other active orbitals vary significantly (Fig. 10(a)). As the Fe–NO bond length increases, the anti-bonding orbitals 
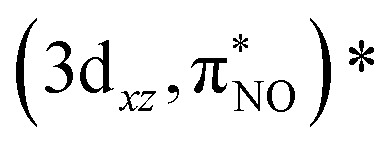
 and 
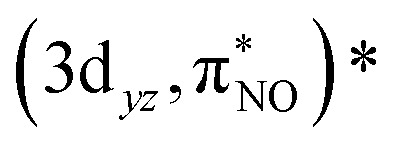
 gain electrons, whereas their pairwise bonding orbitals 
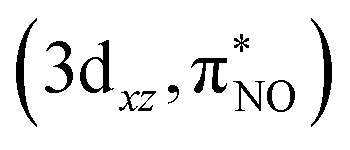
 and 
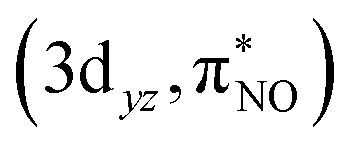
 lose electrons. A similar feature is observed for the orbital pair (π_cor_, 3d_*z*^2^_) and (π_cor_, 3d_*z*^2^_)*. This behavior indicates that Fe–NO elongation drives the iron center toward a Fe^III^ intermediate-spin state, consistent with the electron configuration proposed for the photolysis product of Fe[TPC](NO).^25,78^ Simultaneously, the corrole ligand becomes more non-innocent as the Fe–NO elongates, due to electron donation from π_cor_ to the 3d_*z*^2^_ orbital.

This spin-state evolution is particularly relevant in photochemistry. Our results indicate that Fe–NO bond elongation drives a progression from a low-spin Fe^III^ state in the equilibrium structure toward an intermediate-spin Fe^III^ state at extended Fe–NO distances. Such a low-spin → intermediate-spin transition would be favorable under photolysis, as photoexcitation into Fe–NO antibonding states both weakens Fe–NO π back-bonding and lowers the barrier for NO dissociation. Thus, the photolysis species corresponds to an intermediate-spin Fe^III^ corrole with enhanced radical character on the corrole ligand. The computed elongation pathway therefore rationalizes the observed photolysis product configuration and highlights spin crossover as a key mechanistic step facilitating NO release in corrole systems.

For (b), the energy gap between the two active orbitals was analyzed using CASCI (2o,2e) calculations using (π_cor_, 3d_*z*^2^_) and (π_cor_, 3d_*z*^2^_)* orbitals. The results are shown in Fig. 10(b). The relative energy of the S_0_ state with respect to its reference value at Δ(Fe–NO) = 0 is shown as the blue curve, while the corresponding energy of the T_1_ state is shown in orange. Their energy difference is indicated by the dashed black curve. Fe–NO elongation weakens the axial ligand field, thereby reducing the energy gap between (π_cor_, 3d_*z*^2^_) and (π_cor_, 3d_*z*^2^_)*. As Δ(Fe–NO) increases, this gap narrows, facilitating charge transfer from the corrole π orbital into the Fe 3d_*z*^2^_ orbital. This effect is reflected in the changes of orbital occupations and signals enhanced non-innocence of the corrole ligand.

A central feature emerging from these results is the decisive role of the axial ligand in tuning the Fe 3d orbital energy levels. In Fe[TPC](NO), the axial ligand influences three key pairwise orbital interactions, as indicated above (see also Fig. 10(a)). To examine in more detail how the axial ligand affects the radical character of the corrole, we focus on the specific orbital interaction between the 3d_*z*^2^_ and the corrole π (a_2u_) orbital. Accordingly, we examined the elongation of the Fe–Cl bond in Fe[TPC]Cl. Increasing the Fe–Cl bond length weakens the σ-donor interaction of the chloride with the Fe 3d_*z*^2^_ orbital, thereby stabilizing the 3d_*z*^2^_ level. This reduces the energy gap between the Fe 3d_*z*^2^_ orbital and the corrole π (a_2u_) orbital, enhancing their mixing and promoting charge transfer from the corrole into the metal center, see Fig. 10 (c) and (d). This behavior mirrors the trend observed in the Fe[TPC](NO) system. Notably, the energy gap of Fe[TPC]Cl is significantly smaller than that in Fe[TPC](NO). Indeed, the energy gap of Fe[TPC]Cl without bond elongation closely matches the energy gap of Fe[TPC](NO) at Δ(Fe–NO) = 0.4 Å, as highlighted in Fig. 10(b). This ligand-dependent tuning also mirrors trends observed in Fe–NO porphyrins,^74,75,79^ but in corroles the effect is magnified by the stronger equatorial field, such that modest changes in axial donor/acceptor strength dramatically alter the spin density distribution and ligand non-innocence.

Using all of the results presented here, a striking observation concerning the non-innocence of the corrole ligand in Fe[TPC](NO) can be made: the corrole itself is identified as non-innocent experimentally from its alternating bond distances^2,68^ and from a dependence of the UV-vis spectrum upon *para*-substituent variation.^2^ However, the Fe center does not reflect this and presents itself dominantly as an {FeNO}^6^ unit with a low-spin Fe^III^ center, but without a localized hole in the iron π-t_2g_ orbital due to strong covalent mixing between the iron t_2g_ orbital and NO π* orbitals. However, a small amount of corrole radical character is found in the ground-state wavefunction (see the excited determinant contributions in Table 3). This limited radical character may be sufficient to account for the observed skeletal bond length alternation in Fe[TPC](NO). This interpretation is consistent with earlier observations on tungsten- and molybdenum-dichlorido corroles, where bond length alternation was also observed despite only low corrole radical character on the corrole ring.^80^ In contrast, Ru[TPC](NO) corrole is thought to have a closed-shell corrole, consistent with no bond distance alternation.^81^

## 5 Summary

The ground-state electronic structure of the iron corrole nitrosyl complex (Fe[TPC](NO)) was investigated using a combination of X-ray absorption spectroscopy (XAS), X-ray emission spectroscopy (XES), and multiconfigurational calculations. This work addresses the longstanding debate regarding the electronic structure of Fe[TPC](NO), specifically the radical nature of the corrole ligand and the spin/oxidation state of the iron center.

Experimental X-ray data confirm that the Fe center in Fe[TPC](NO) adopts a low-spin configuration, best described as {FeNO}^6^ with substantial π back-bonding. Multiconfigurational calculations of the full Fe[TPC](NO) complex, a computational model [FeNO] and Fe[TPC](NO) structures with systematically varied Fe–NO bond lengths consistently support this assignment. In this framework, the ground state corresponds to a low-spin Fe^III^ center in which no localized hole resides in the iron t_2g_ orbitals, owing to strong covalent mixing with the NO π* orbitals. This description is fully consistent with the first-moment position of the Kβ XES, the XAS edge shift, and the significant π* back-donation contributions observed in both the L-edge and K pre-edge XAS spectra.

A comparative investigation with Fe[TPC]Cl highlights the decisive role of the axial ligand in tuning the electronic structure. While Fe[TPC](NO) shows only minor corrole radical character, Fe[TPC]Cl exhibits a pronounced radical character, emphasizing how the axial ligand influences the Fe–corrole electronic structure. Variations in the Fe–axial ligand bond length further illustrate this flexibility: elongation narrows the 3d_*z*^2^_–a_2u_ gap, driving the system toward an intermediate-spin Fe^III^ configuration with enhanced corrole radical character. This evolution provides a natural explanation for the photolysis of Fe[TPC](NO), in which photoexcitation into Fe–NO antibonding states promotes a low-spin → intermediate-spin crossover that lowers the barrier for NO release.

Together, these results demonstrate that axial ligands actively reshape the orbital energy landscape that controls spin and charge distribution in corrole systems. By integrating multiconfigurational calculations with metal-centered core-level spectroscopy, this study establishes a robust framework for disentangling metal–ligand covalency and ligand non-innocence. Beyond resolving the ground-state electronic structure of Fe[TPC](NO), these findings underscore the broader principle that macrocyclic contraction and axial-ligand tuning strongly influence spin dynamics and photochemical reactivity in non-innocent metallomacrocycles.

## Author contributions

M. Guo, A. Braun, S. Lee, D. Sokaras, and T. Kroll performed the experimental data collection. M. Guo and T. Kroll analysed the data. M. Guo designed and performed all theoretical calculations and analyses. A. B. Alemayehu performed sample synthesis. E. I. Solomon, A. Ghosh and T. Kroll supervised the project. M. Guo and T. Kroll wrote the manuscript with input and critical revisions from all authors.

## Conflicts of interest

There are no conflicts to declare.

## Supplementary Material

SC-OLF-D6SC00669H-s001

SC-OLF-D6SC00669H-s002

## Data Availability

The data supporting this article have been included as part of the supplementary information (SI). Supplementary information: additional computational and spectroscopic analyses including active spaces and orbitals used in multiconfigurational calculations, orbital contributions to calculated Fe K- and L-edge X-ray absorption spectra, spin-resolved transition analyses, representative localized orbitals, benchmarking of CASSCF active spaces for model [FeNO]^2+^/[FeNO]^3+^ units, comparison of calculated K pre-edge spectra for model [FeNO]^3+^ units and Fe[TPC](NO), and DFT-optimized structures of Fe[TPC](NO) in close-shell singlet and broken-symmetry singlet states. See DOI: https://doi.org/10.1039/d6sc00669h.
