# Supplementary figures and images for: Metal-centered X-ray absorption and emission spectroscopy of iron corroles: implications for ligand non-innocence
Source: Chem Sci. 2026 Mar 10;17(18):9230–45. doi: 10.1039/d6sc00669h (PMC13001668; doi:10.1039/d6sc00669h)

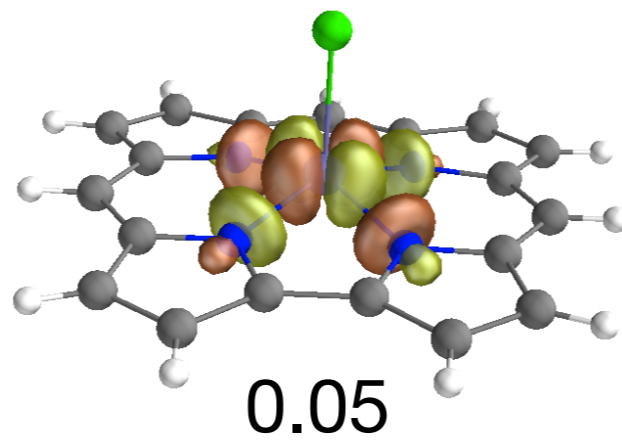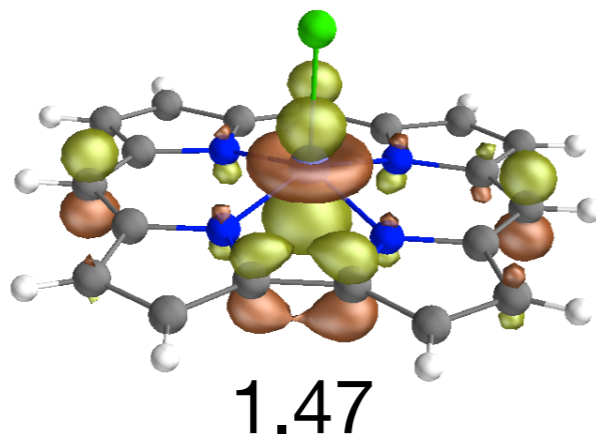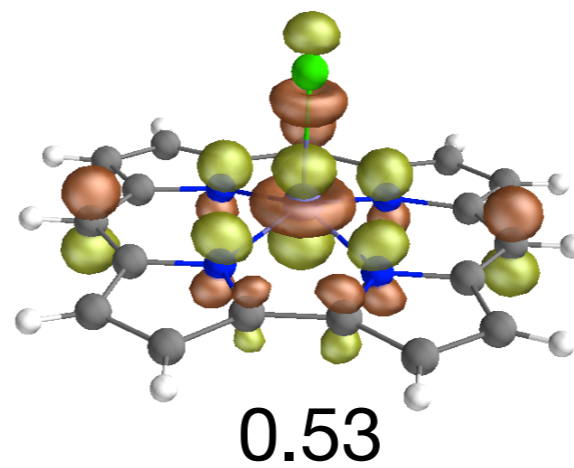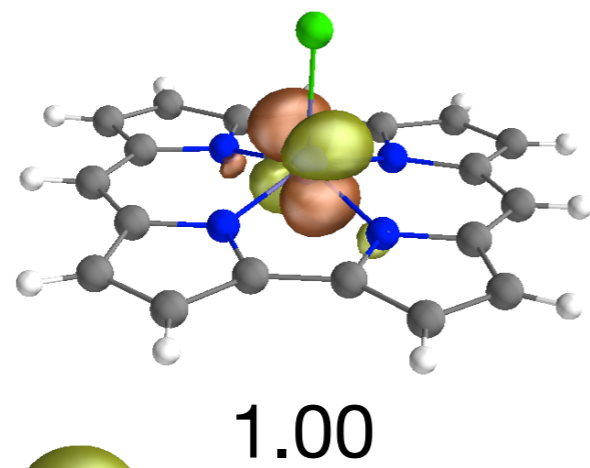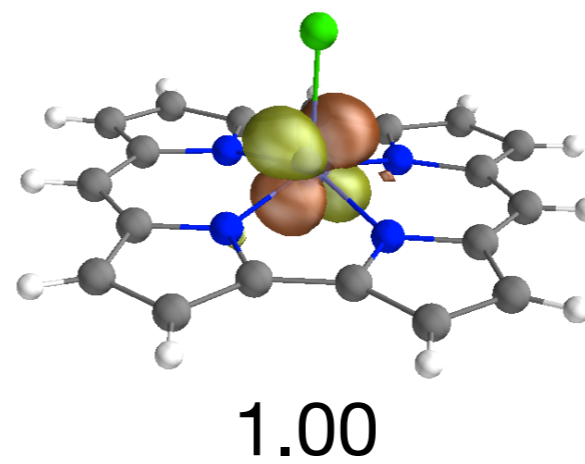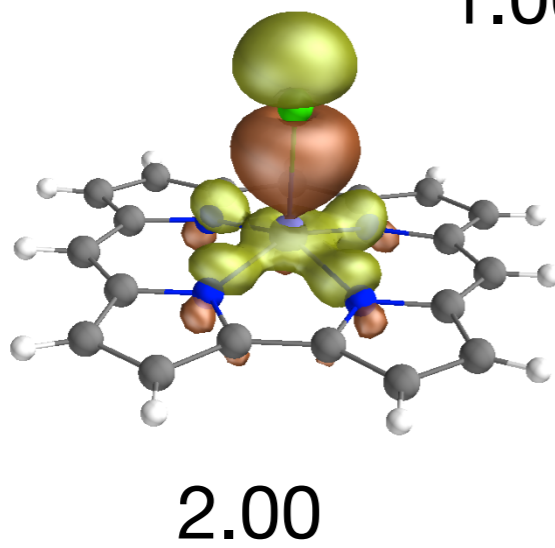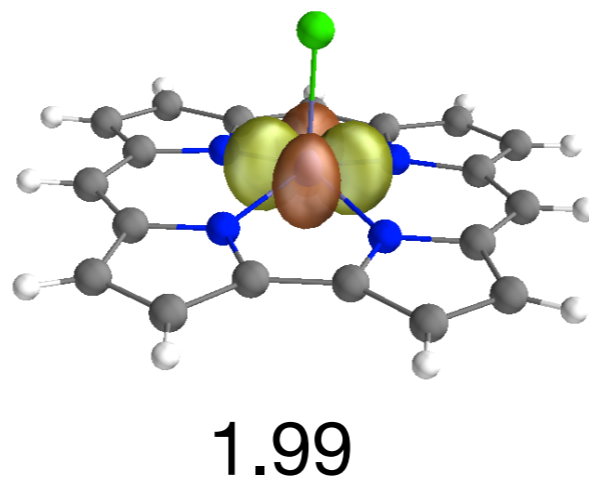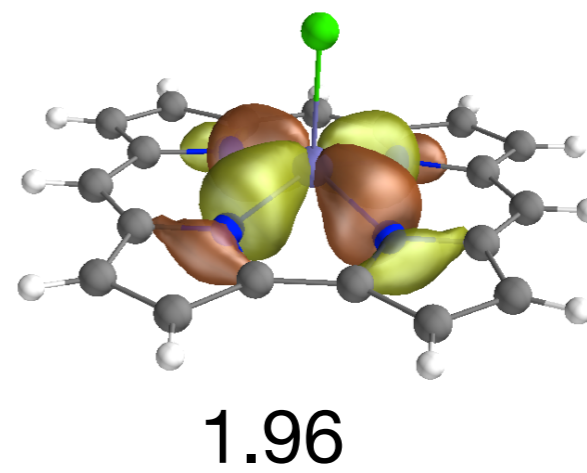

Supplement: SC-017-D6SC00669H-s001 [file SC-017-D6SC00669H-s001.zip › SI-figures/active-orbitals-FeTPC-Cl.pdf]

# {FeNO}<sup>7</sup> doublet

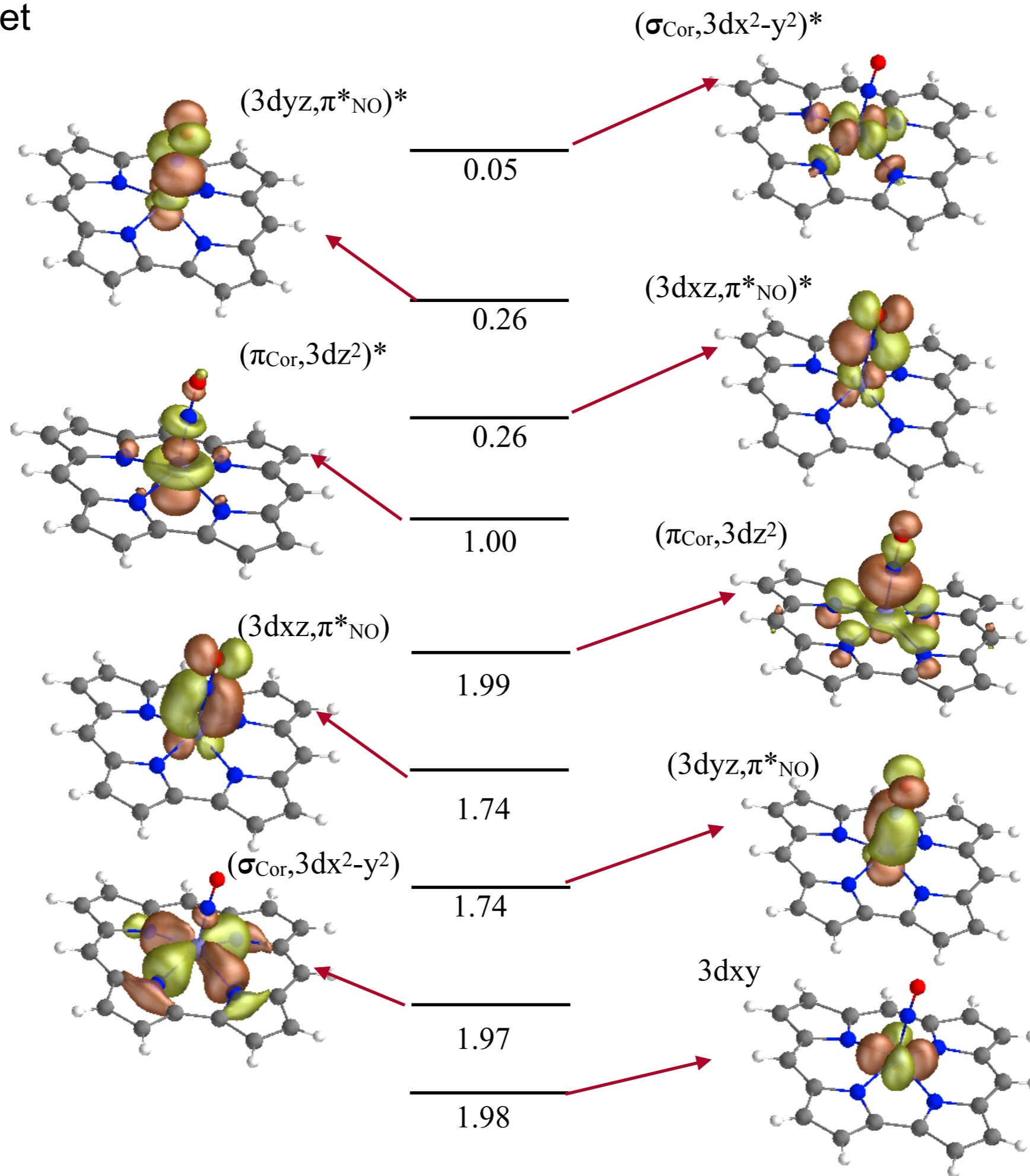

Supplement: SC-017-D6SC00669H-s001 [file SC-017-D6SC00669H-s001.zip › SI-figures/active-space-doublet.pdf]

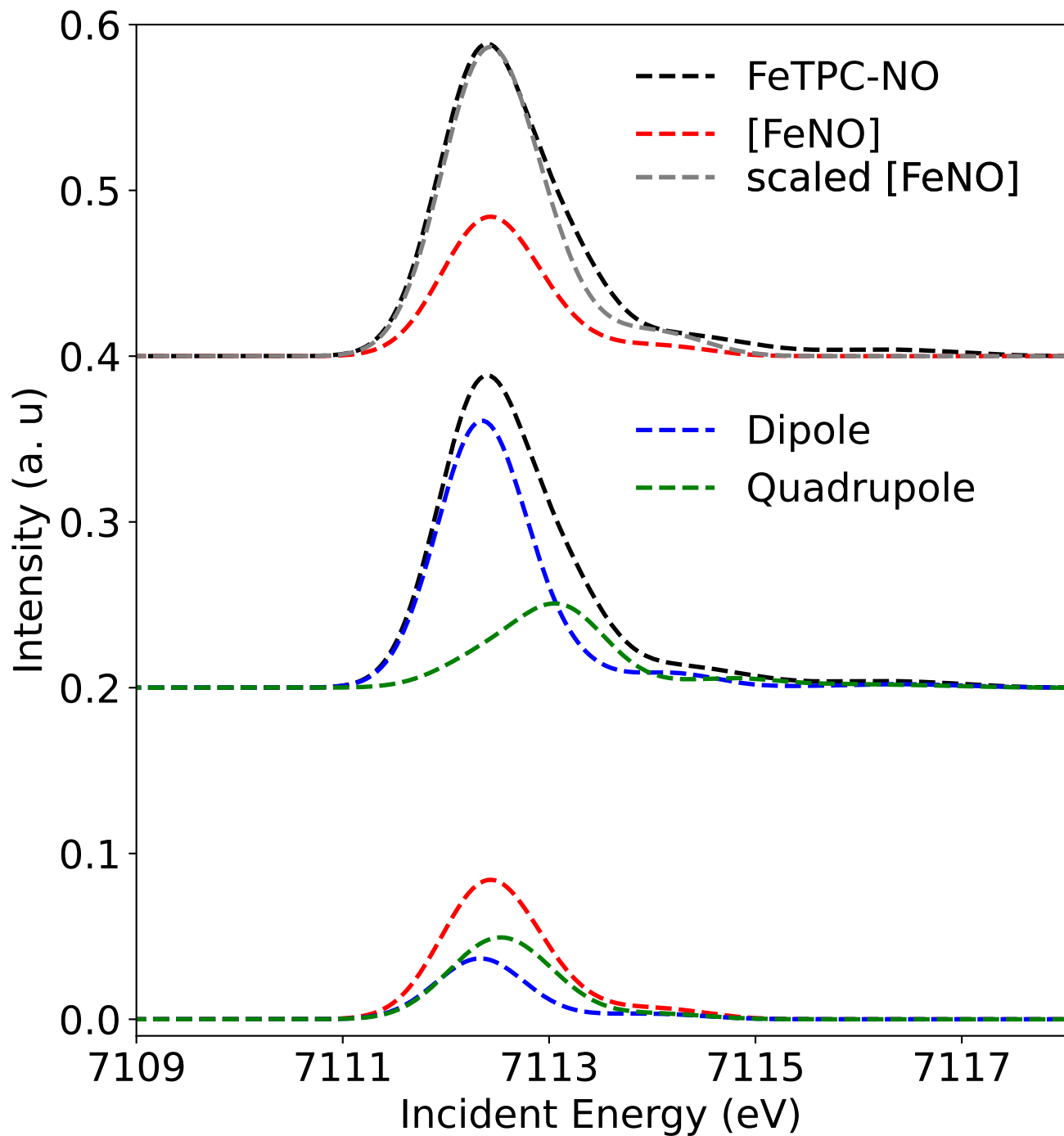

Supplement: SC-017-D6SC00669H-s001 [file SC-017-D6SC00669H-s001.zip › SI-figures/cal-kedge-feno-fetpc-no.pdf]

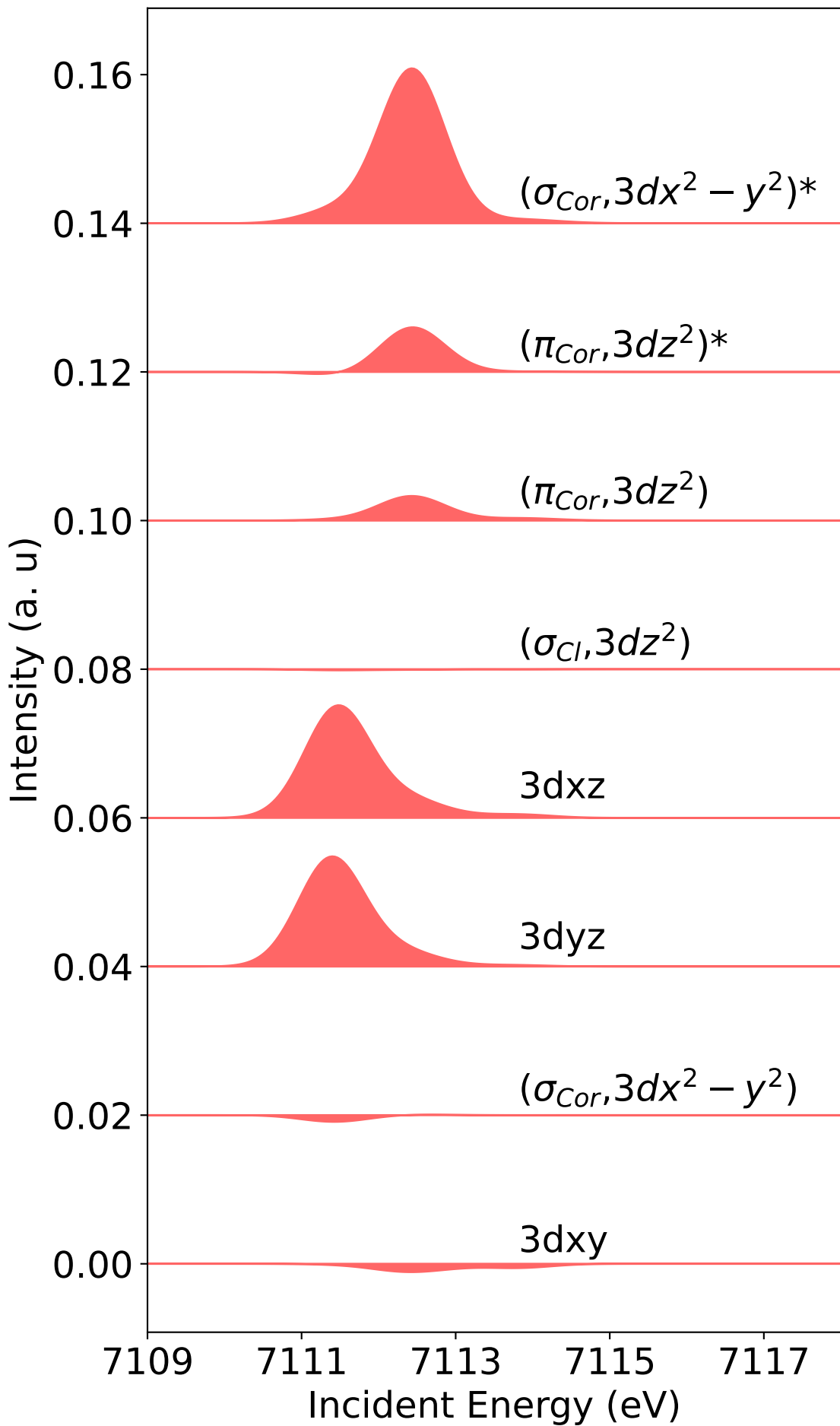

Supplement: SC-017-D6SC00669H-s001 [file SC-017-D6SC00669H-s001.zip › SI-figures/fetpc-cl-k-quadrupole-only.pdf]

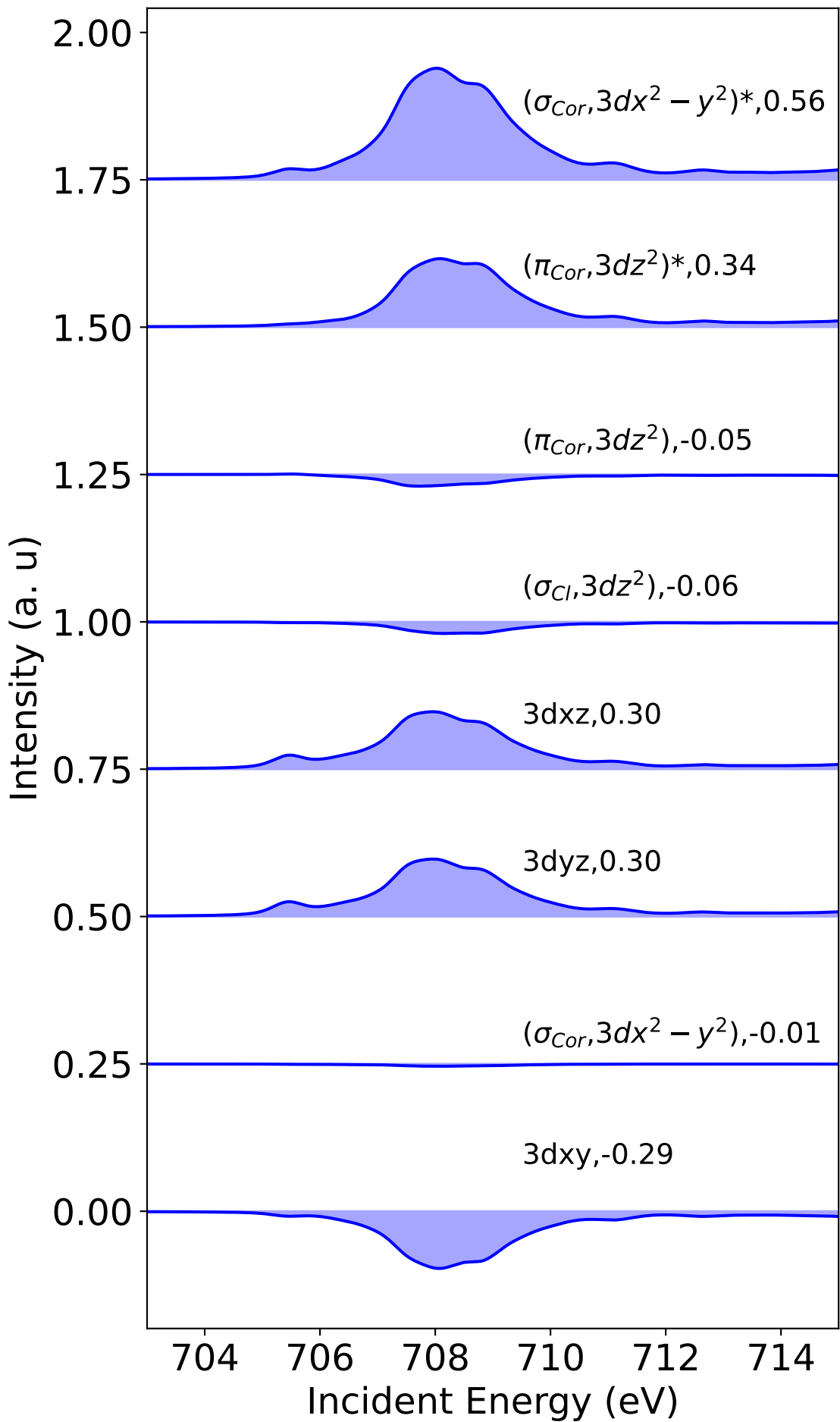

Supplement: SC-017-D6SC00669H-s001 [file SC-017-D6SC00669H-s001.zip › SI-figures/fetpc-cl-l3-orb-contribution-area.pdf]

# Quintet core-excited states

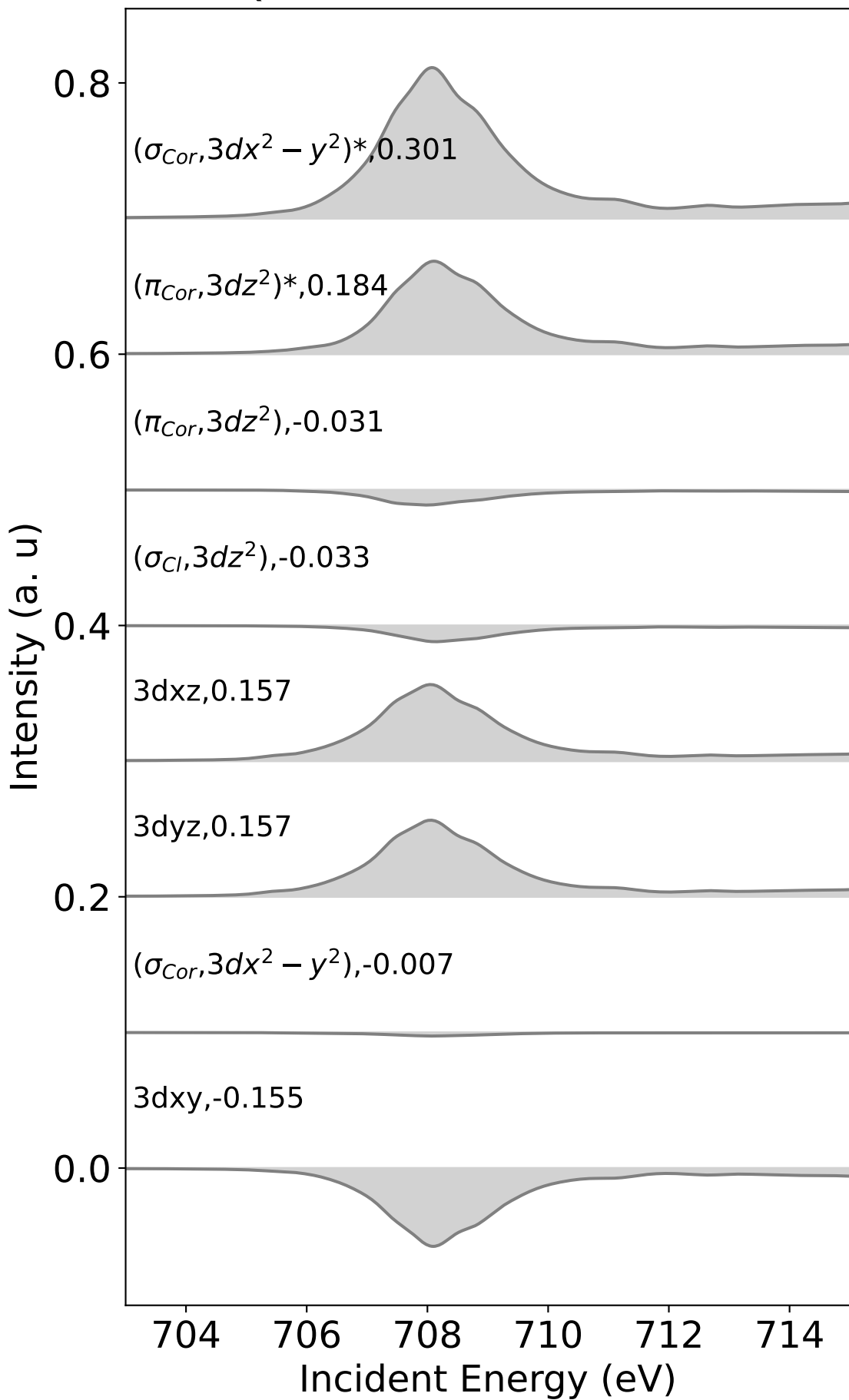

Supplement: SC-017-D6SC00669H-s001 [file SC-017-D6SC00669H-s001.zip › SI-figures/fetpc-cl-l3-orb-contribution-with-area_quintet.pdf]

# Singlet core-excited states

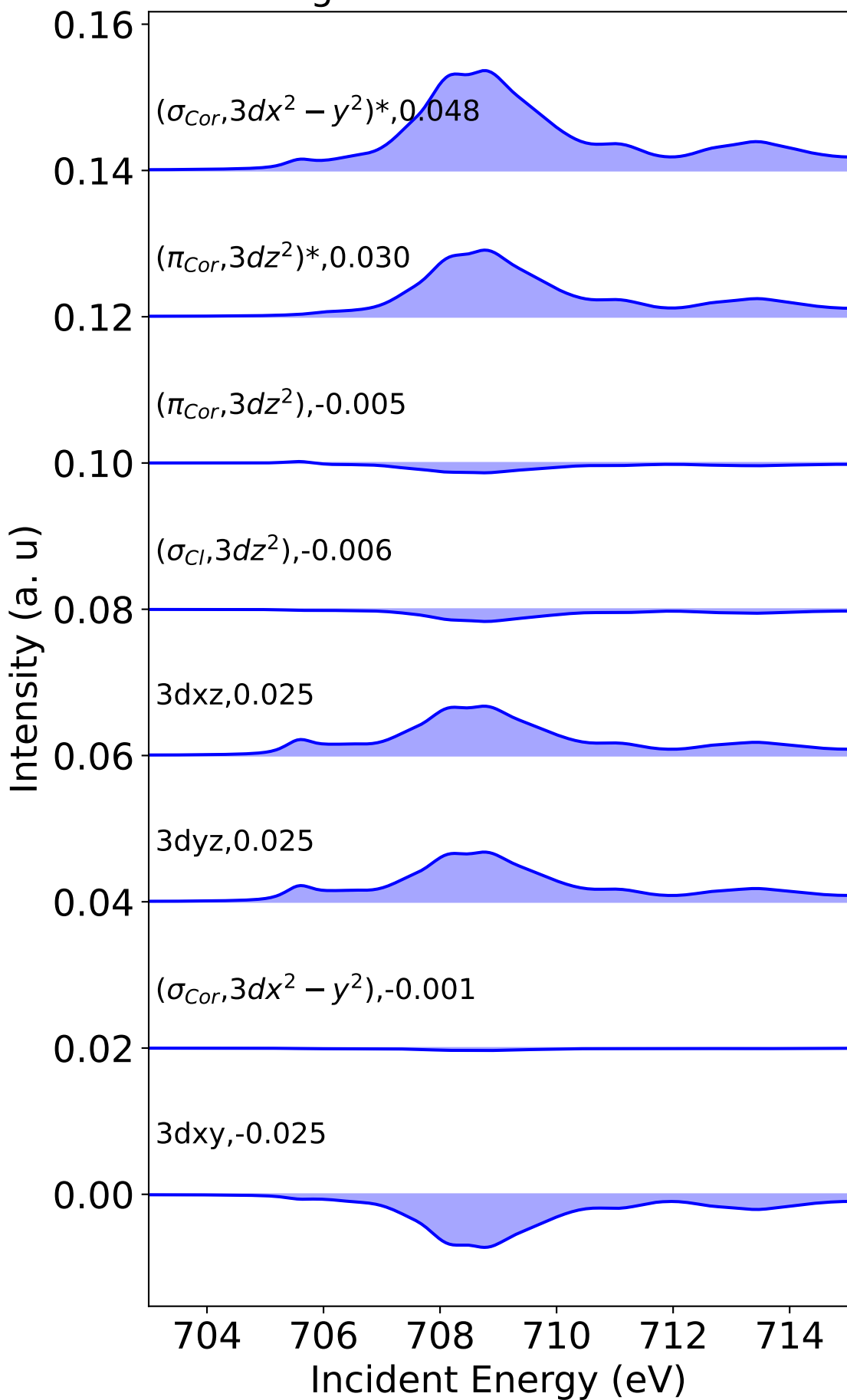

Supplement: SC-017-D6SC00669H-s001 [file SC-017-D6SC00669H-s001.zip › SI-figures/fetpc-cl-l3-orb-contribution-with-area_singlet.pdf]

# Triplet core-excited states

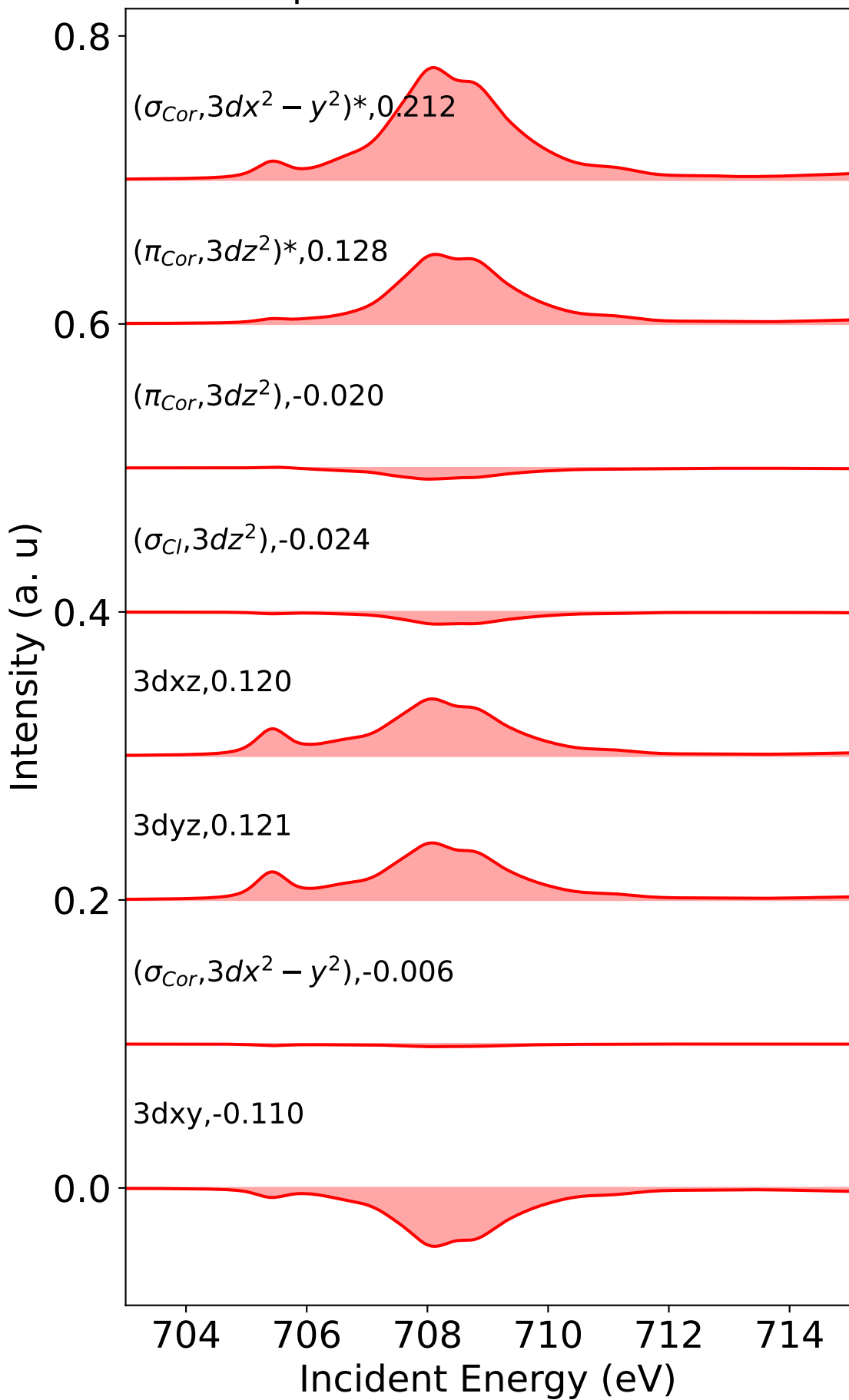

Supplement: SC-017-D6SC00669H-s001 [file SC-017-D6SC00669H-s001.zip › SI-figures/fetpc-cl-l3-orb-contribution-with-area_triplet.pdf]

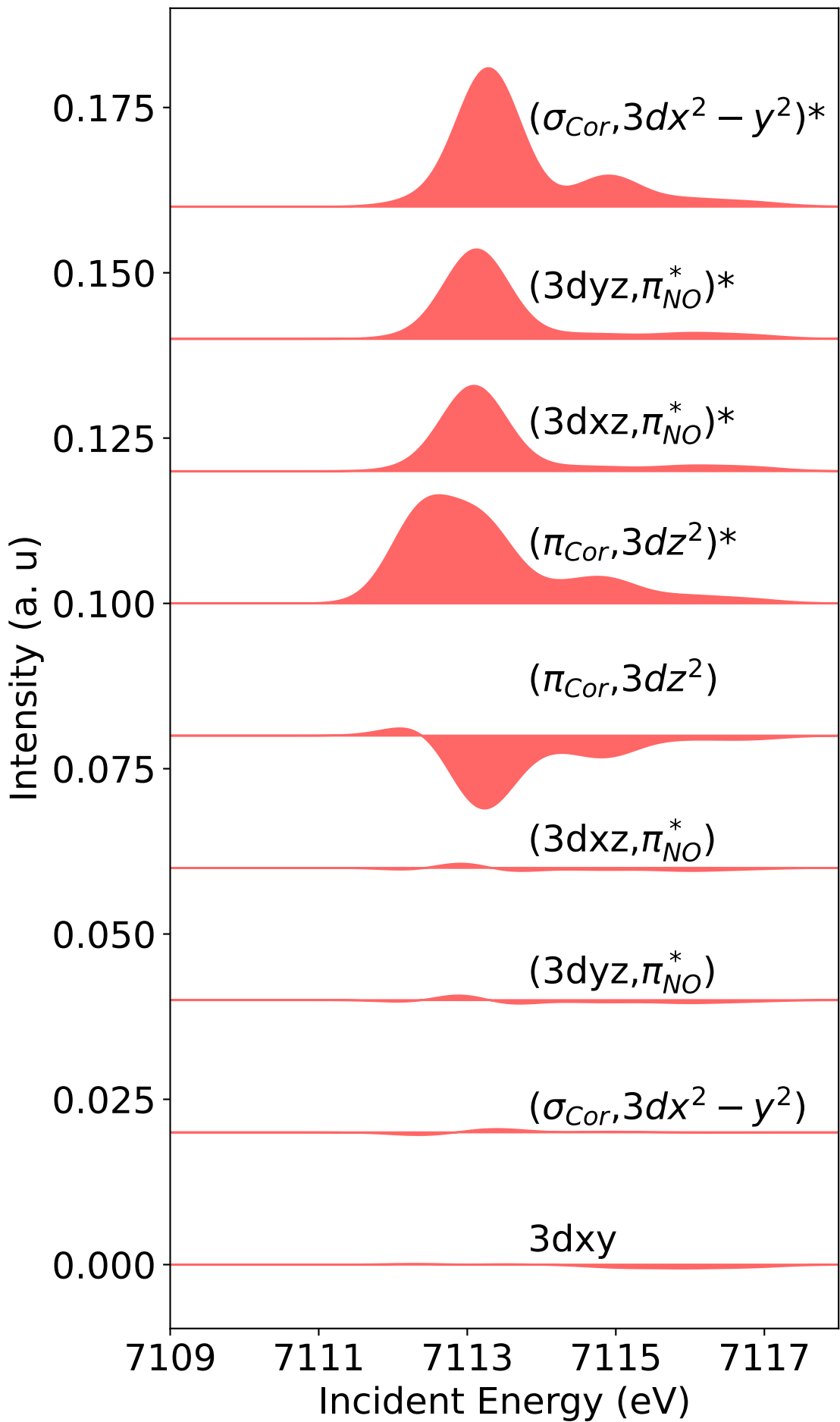

Supplement: SC-017-D6SC00669H-s001 [file SC-017-D6SC00669H-s001.zip › SI-figures/fetpc-no-k-quadrupole-only.pdf]

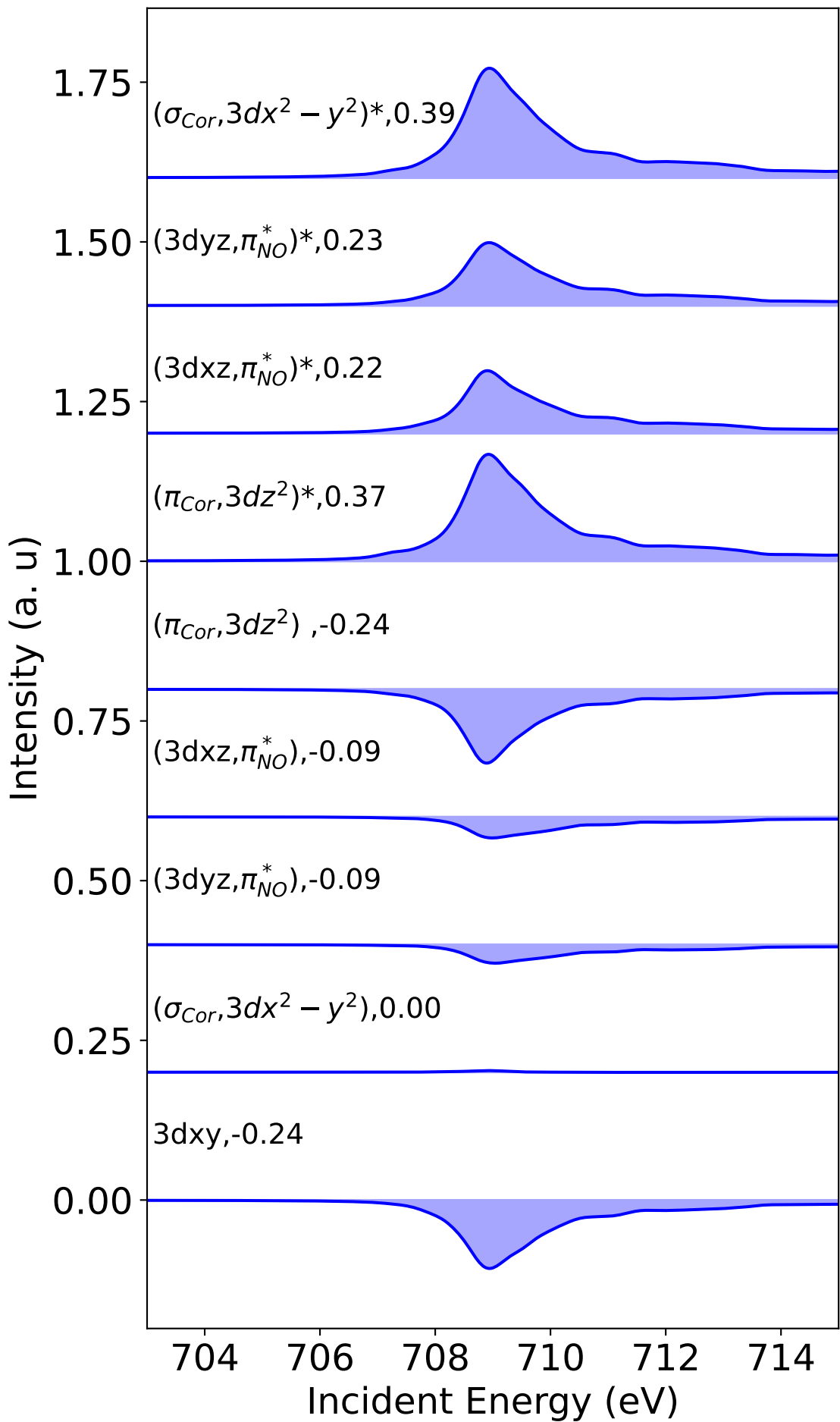

Supplement: SC-017-D6SC00669H-s001 [file SC-017-D6SC00669H-s001.zip › SI-figures/fetpc-no-l3-orb-contribution-area.pdf]

# Quintet core-excited states

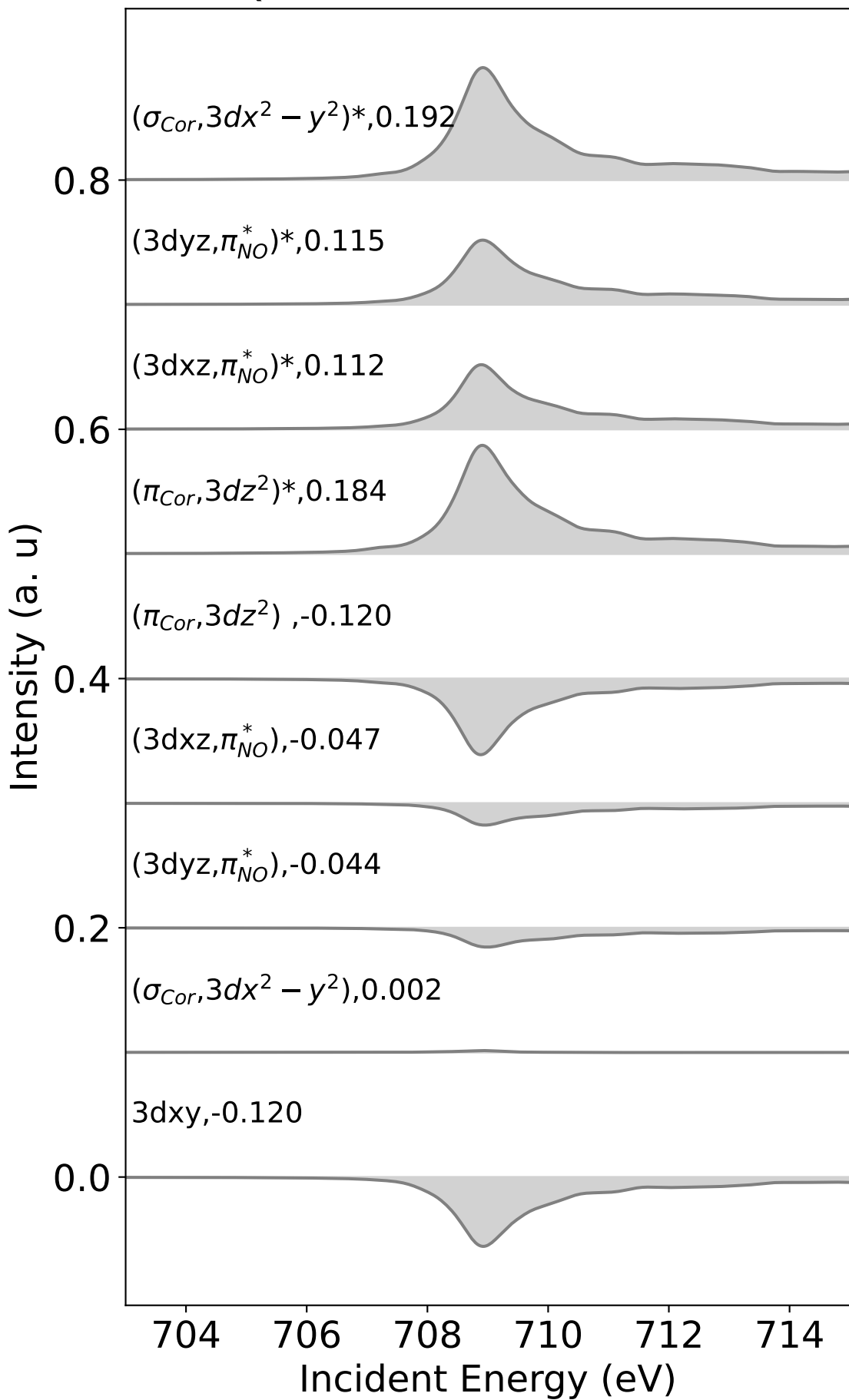

Supplement: SC-017-D6SC00669H-s001 [file SC-017-D6SC00669H-s001.zip › SI-figures/fetpc-no-l3-orb-contribution-with-area_quintet.pdf]

# Singlet core-excited states

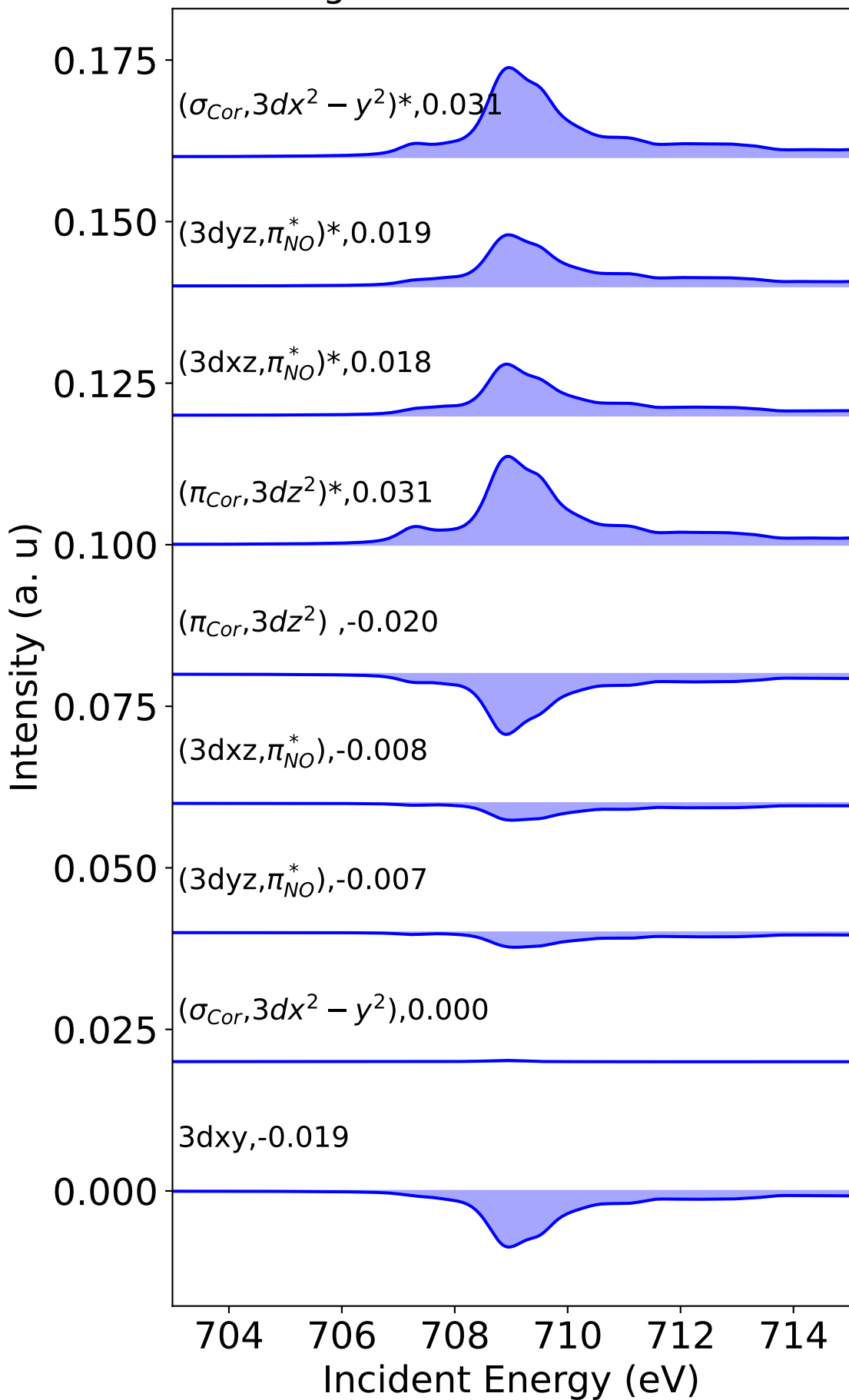

Supplement: SC-017-D6SC00669H-s001 [file SC-017-D6SC00669H-s001.zip › SI-figures/fetpc-no-l3-orb-contribution-with-area_singlet.pdf]

# Triplet core-excited states

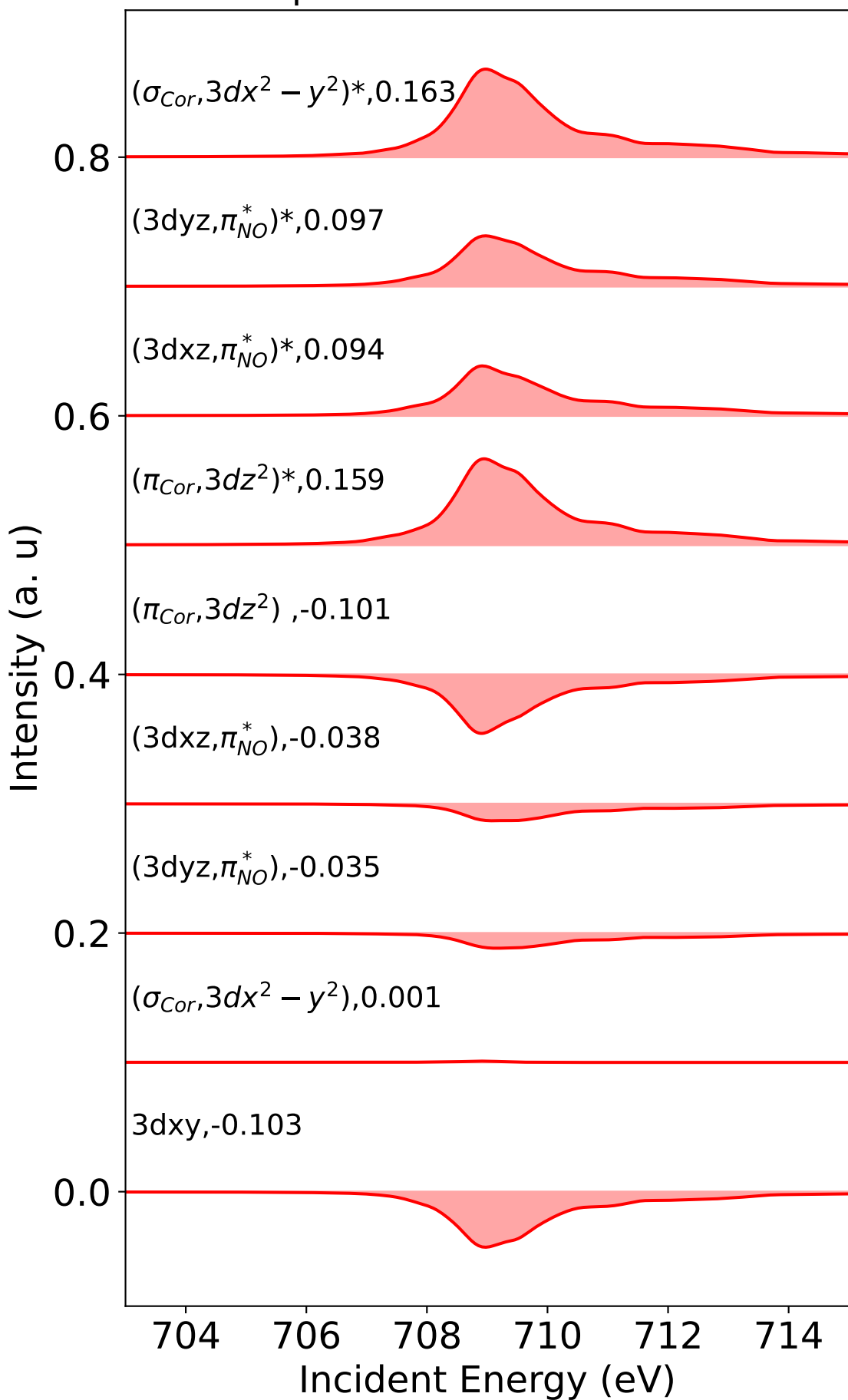

Supplement: SC-017-D6SC00669H-s001 [file SC-017-D6SC00669H-s001.zip › SI-figures/fetpc-no-l3-orb-contribution-with-area_triplet.pdf]

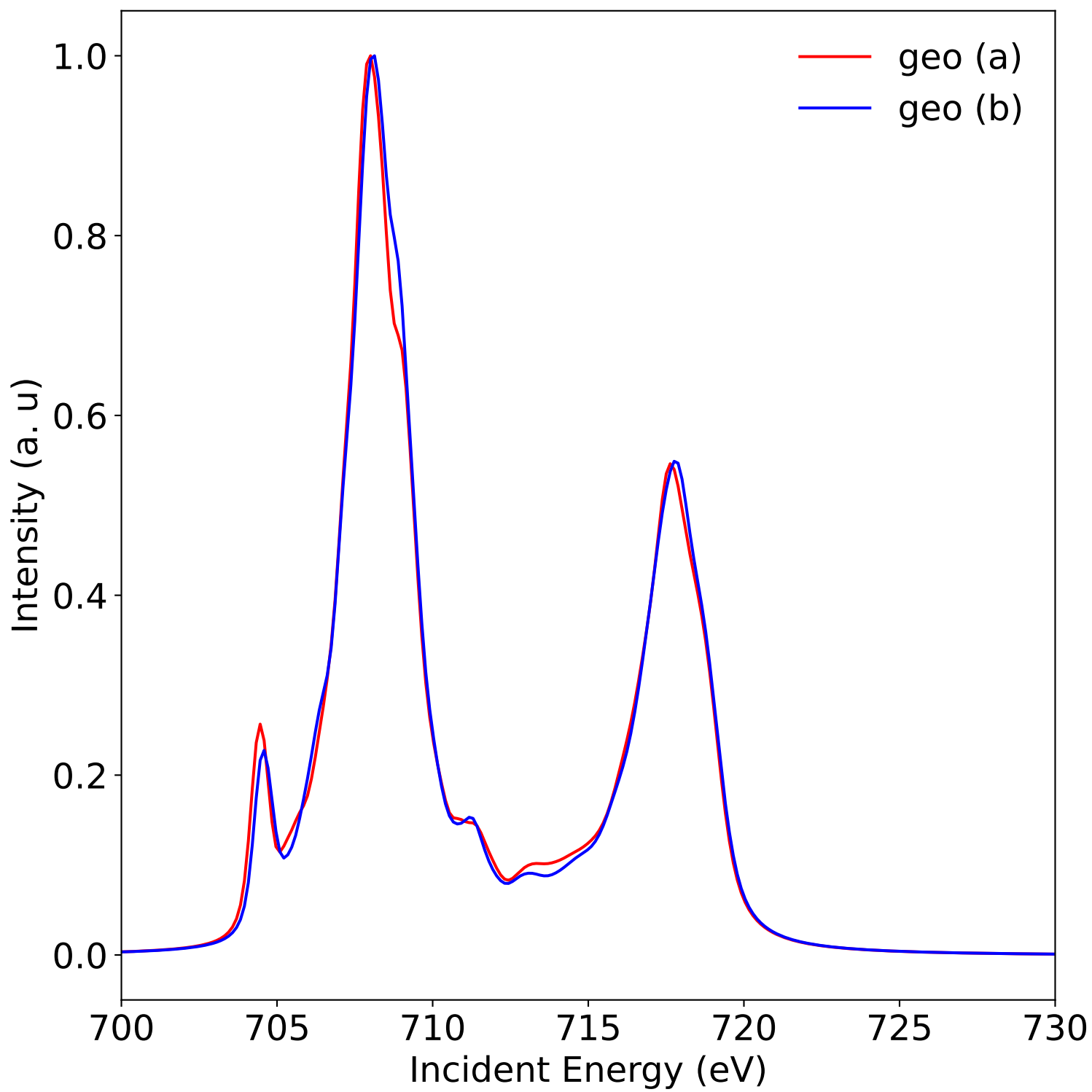

Supplement: SC-017-D6SC00669H-s001 [file SC-017-D6SC00669H-s001.zip › SI-figures/geo-L-edge-comparison-fetpc-cl.pdf]

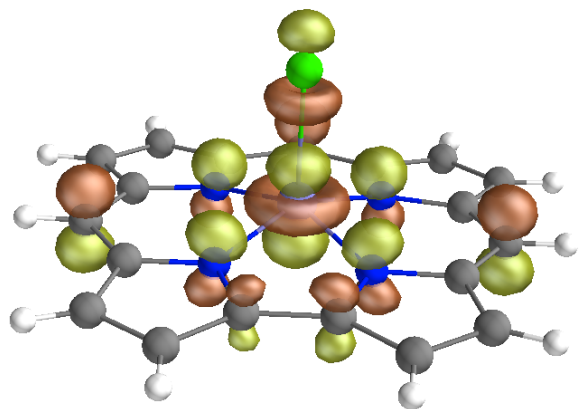

$(\pi_{\text{Cor}}, 3d_{z^2})^*$

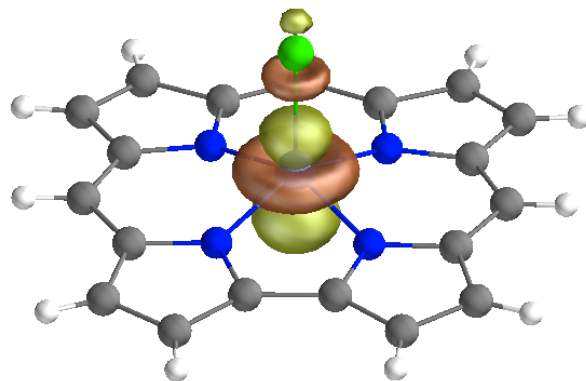

$(3d_{z^2})$

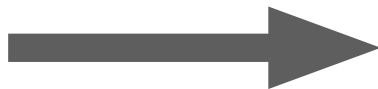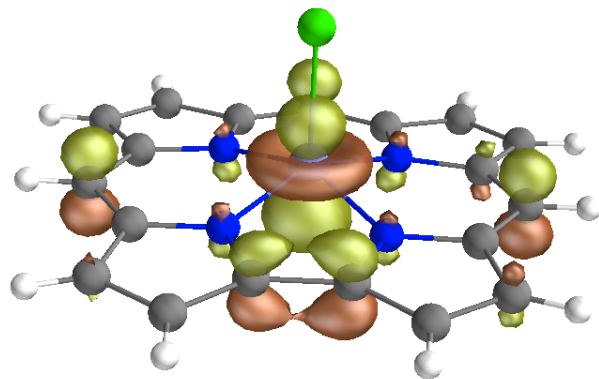

$(\pi_{\text{Cor}}, 3d_{z^2})$

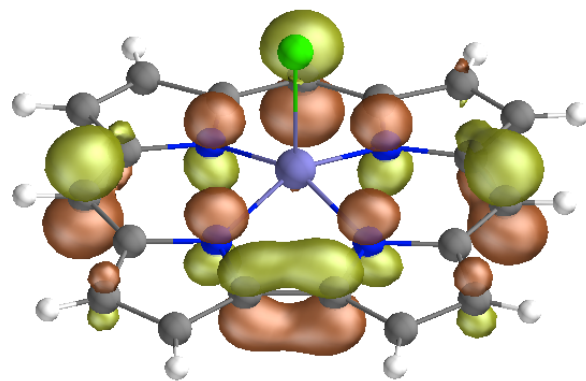

$(\pi_{\text{Cor}})$

Supplement: SC-017-D6SC00669H-s001 [file SC-017-D6SC00669H-s001.zip › SI-figures/localized-orbitals-fetpccl.pdf]

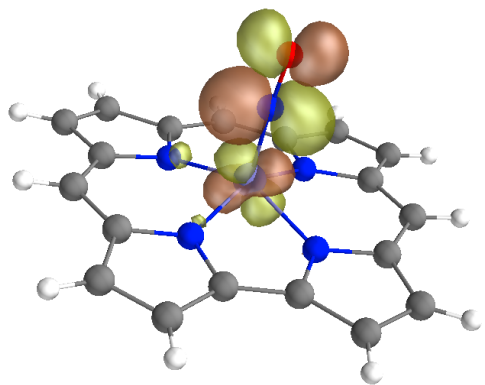

$(3dxz, \pi^*_{NO})^*$

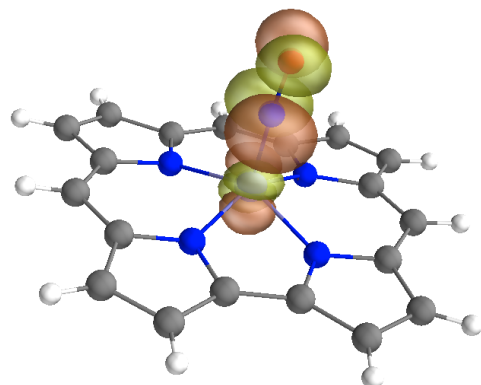

$(3dyz, \pi^*_{NO})^*$

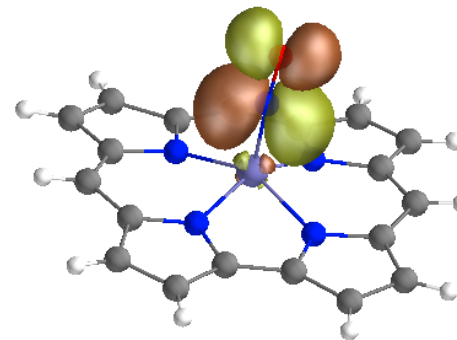

$(\pi^*_{NO})$

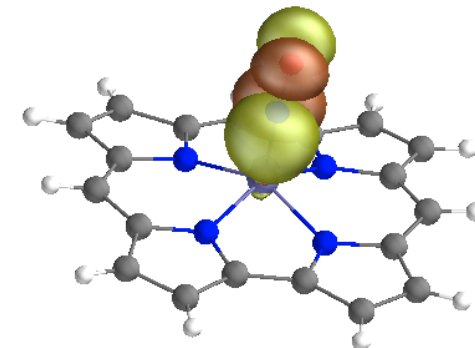

$(\pi^*_{NO})$

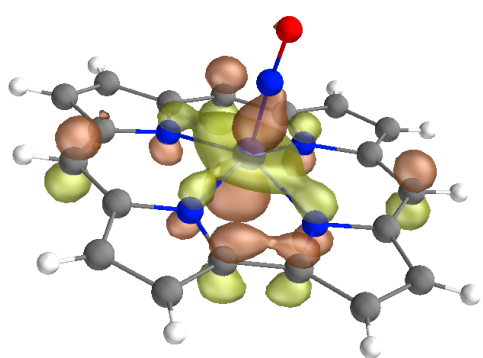

$(\pi_{Cor}, 3dz^2)$

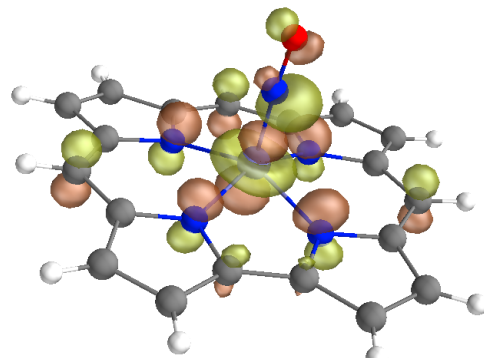

$(\pi_{Cor}, 3dz^2)^*$

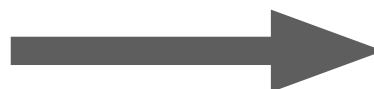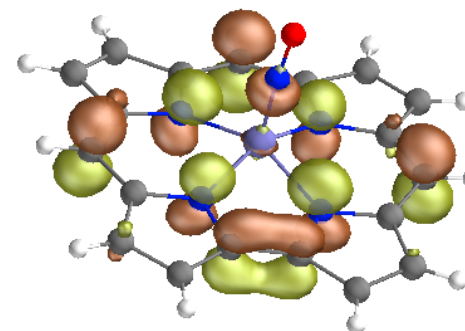

$(\pi_{Cor})$

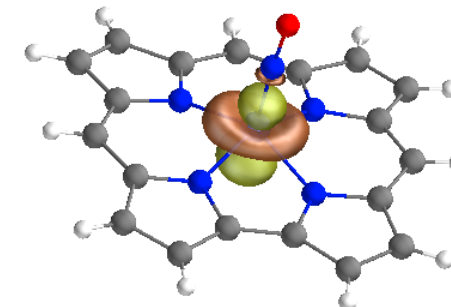

$(3dz^2)$

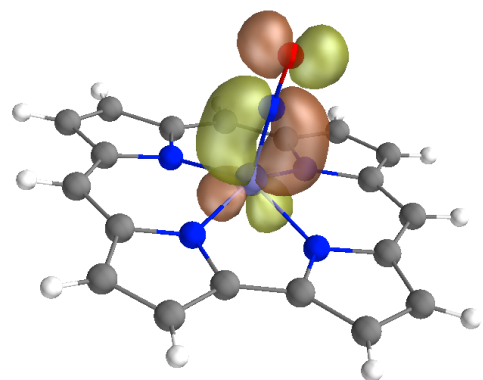

$(3dxz, \pi^*_{NO})$

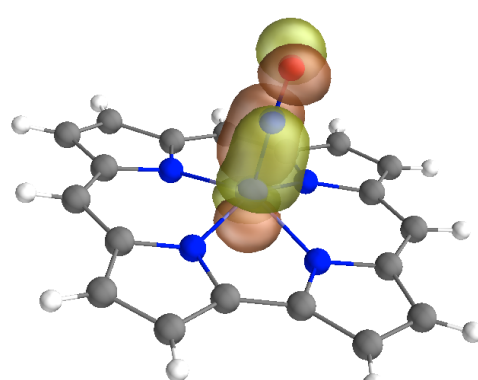

$(3dyz, \pi^*_{NO})$

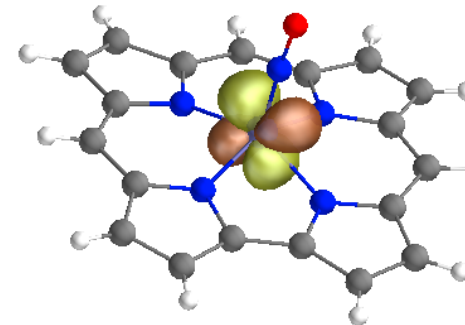

$(3dxz)$

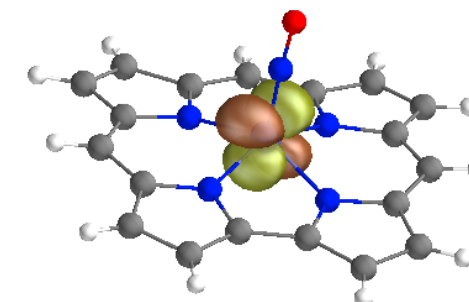

$(3dyz)$

Supplement: SC-017-D6SC00669H-s001 [file SC-017-D6SC00669H-s001.zip › SI-figures/localized-orbitals-fetpcno.pdf]

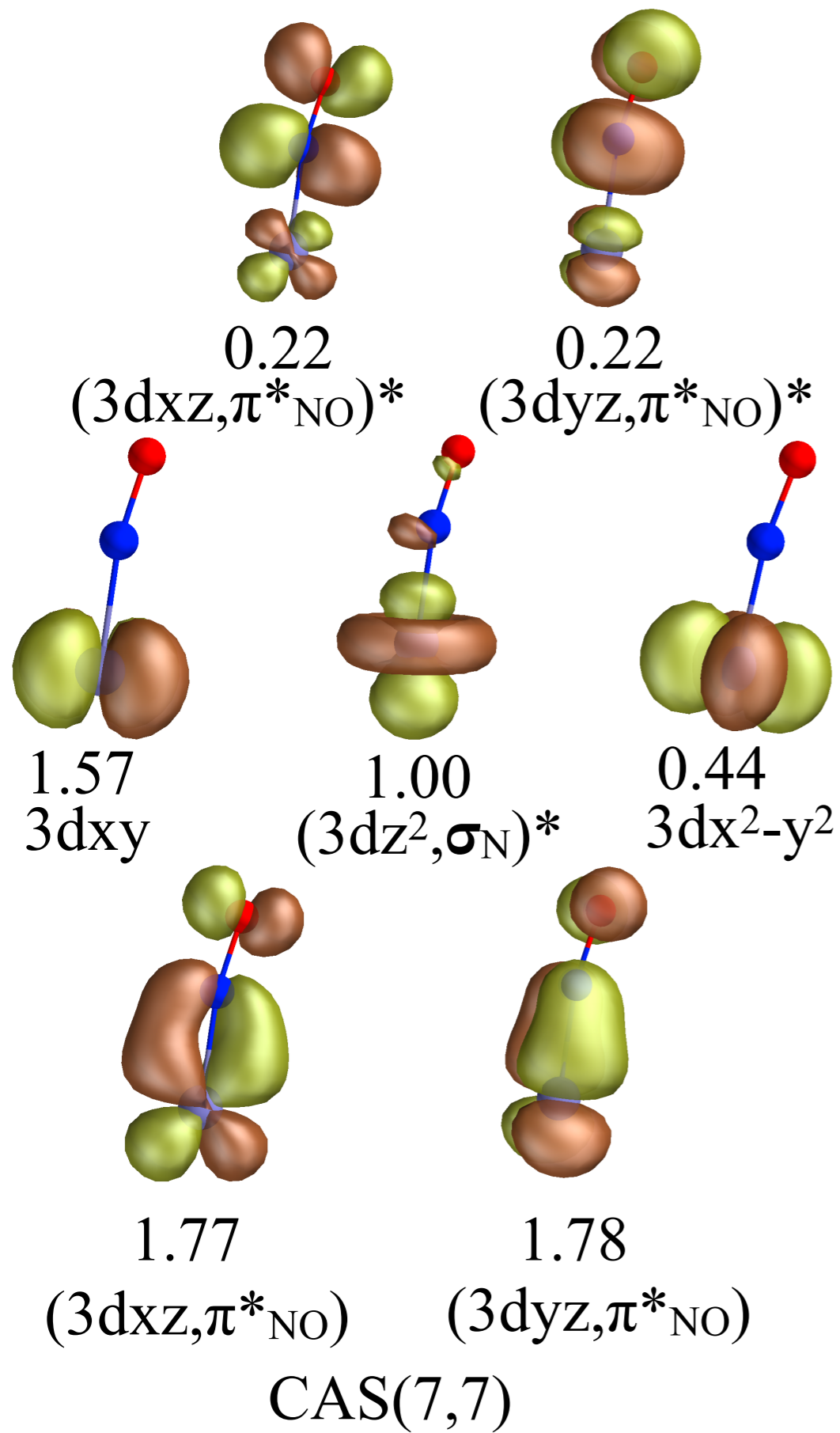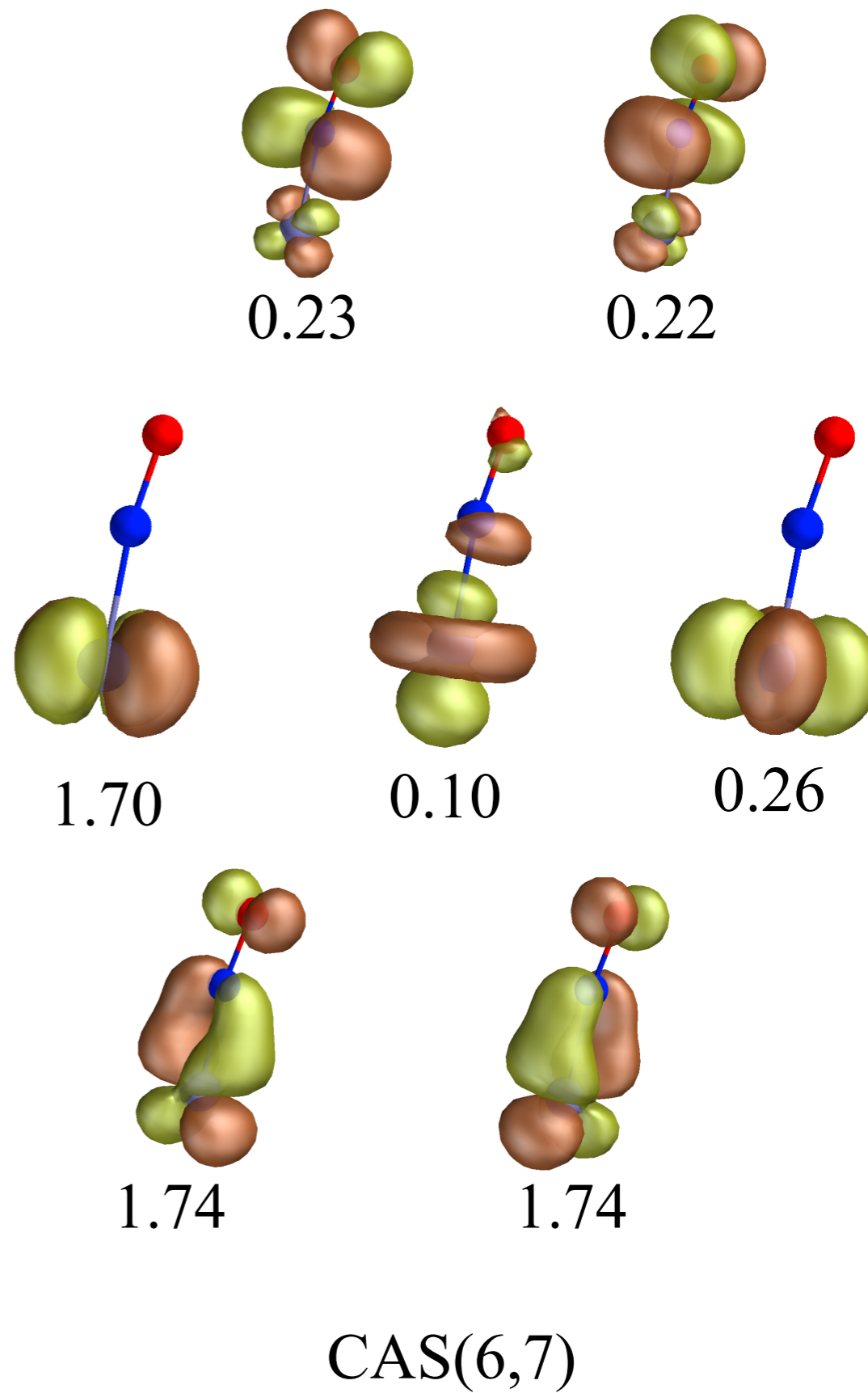

Supplement: SC-017-D6SC00669H-s001 [file SC-017-D6SC00669H-s001.zip › SI-figures/natural-orbitals.001.pdf]

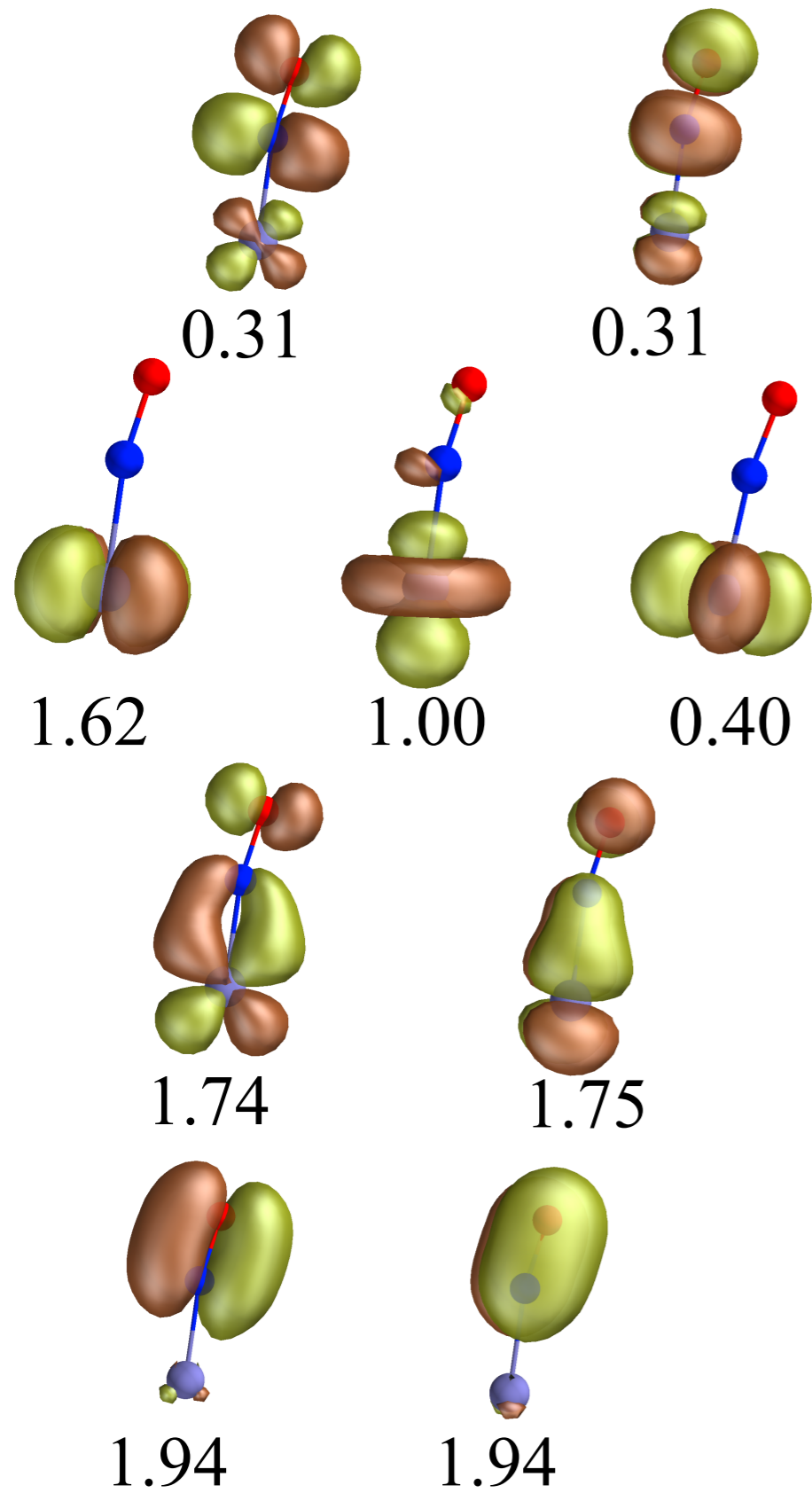

CAS(11,9)

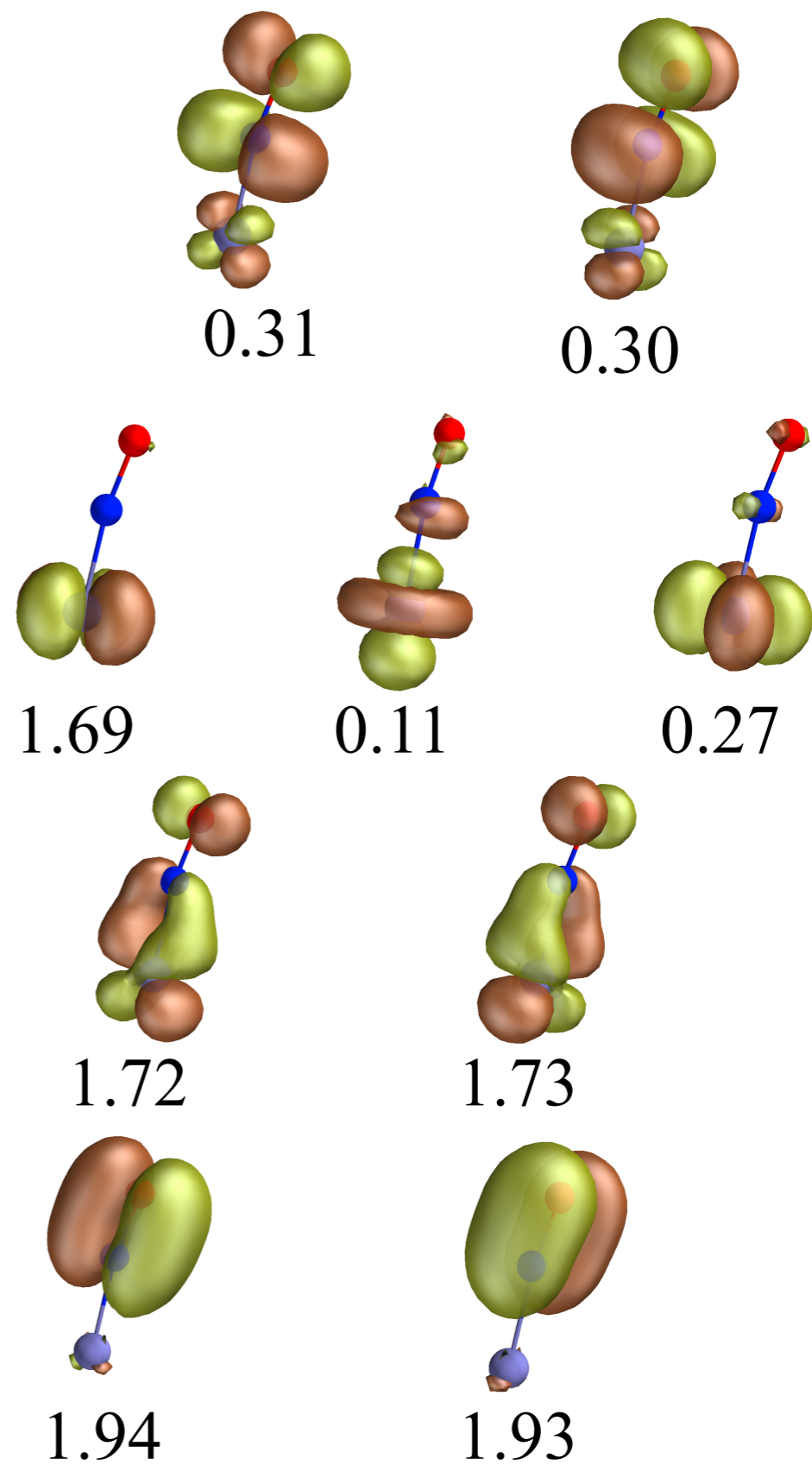

CAS(10,9)

Supplement: SC-017-D6SC00669H-s001 [file SC-017-D6SC00669H-s001.zip › SI-figures/natural-orbitals.002.pdf]

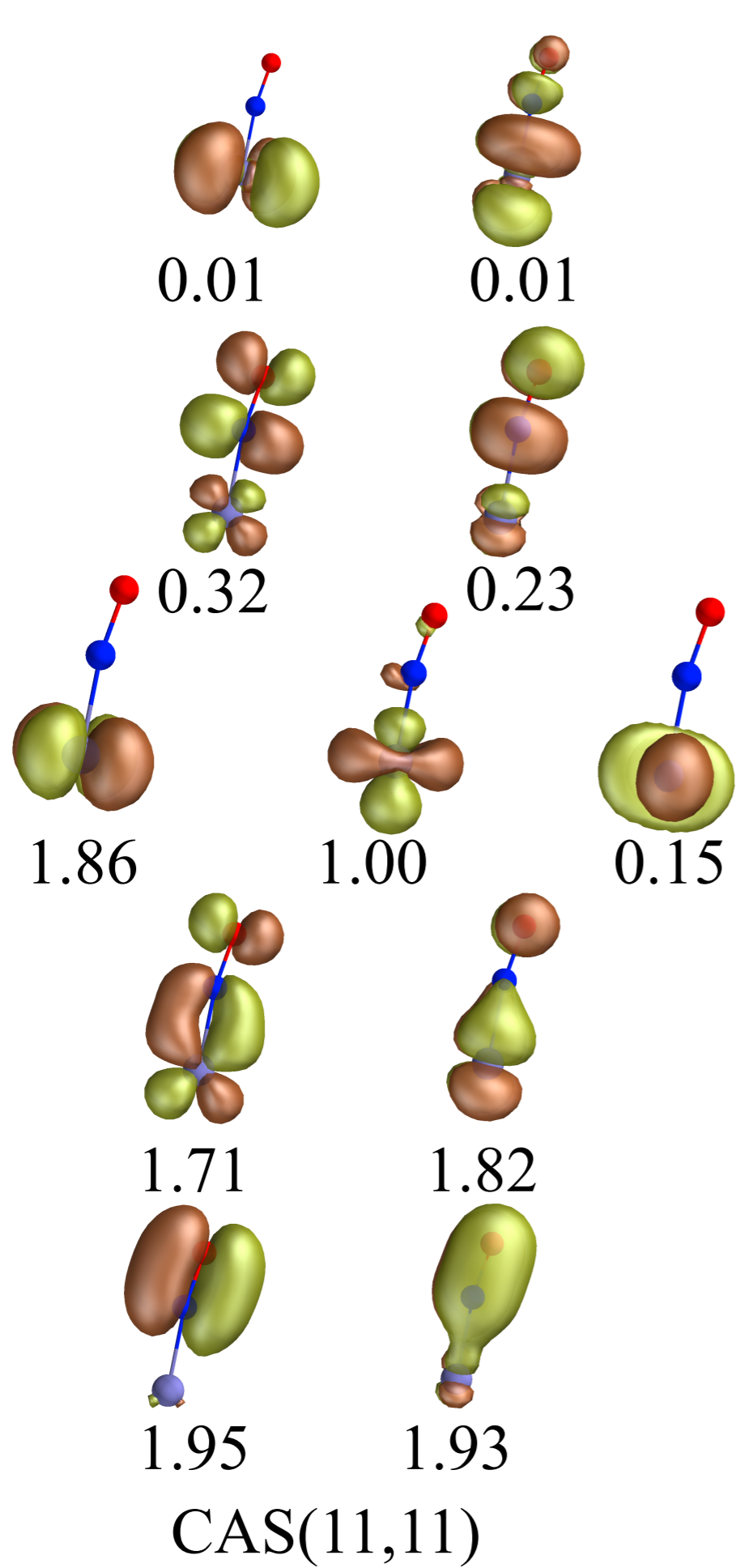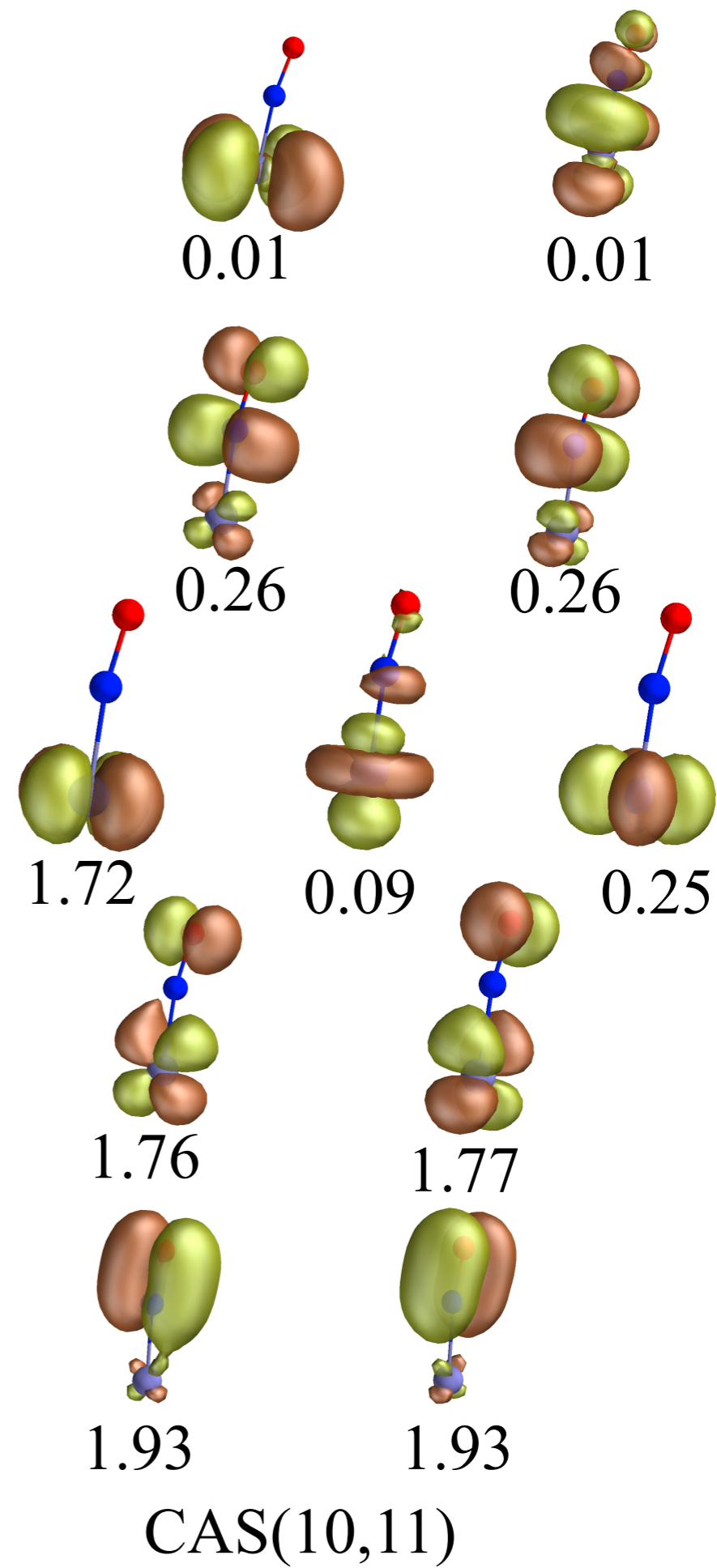

Supplement: SC-017-D6SC00669H-s001 [file SC-017-D6SC00669H-s001.zip › SI-figures/natural-orbitals.003.pdf]

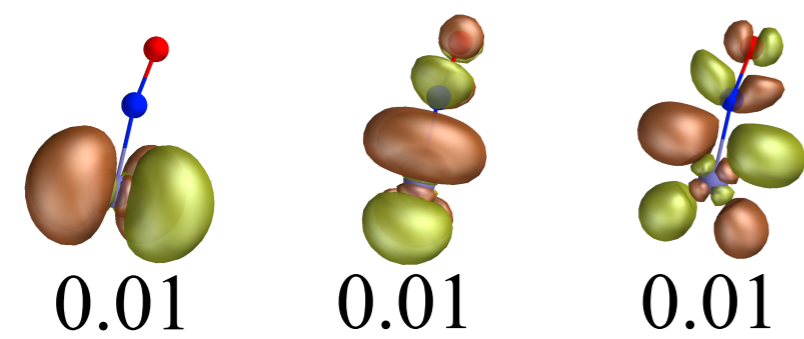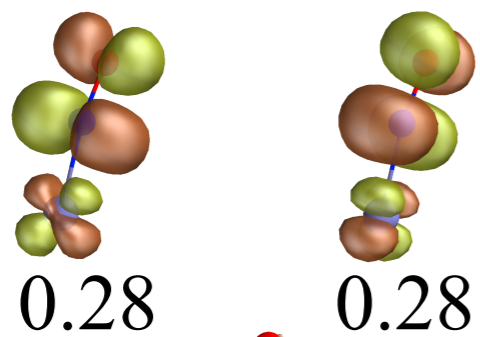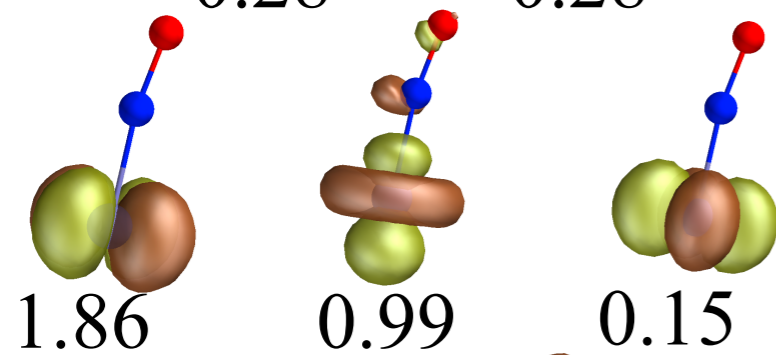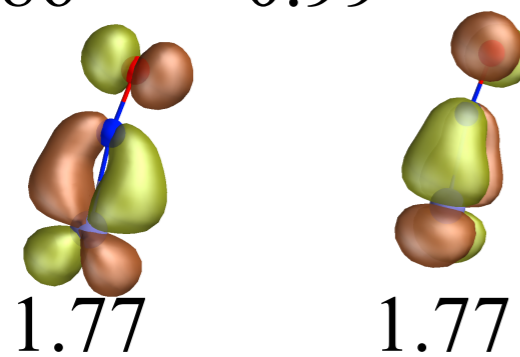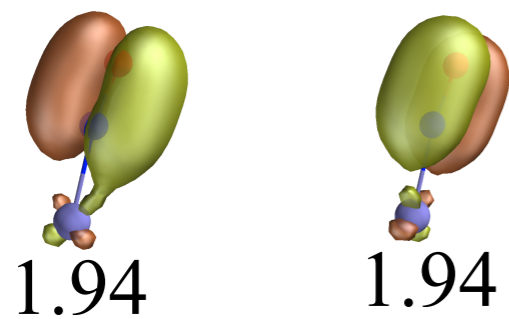

CAS(11,12)

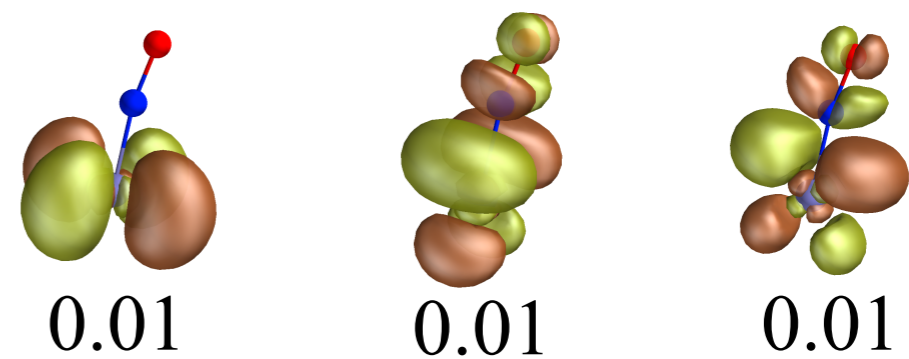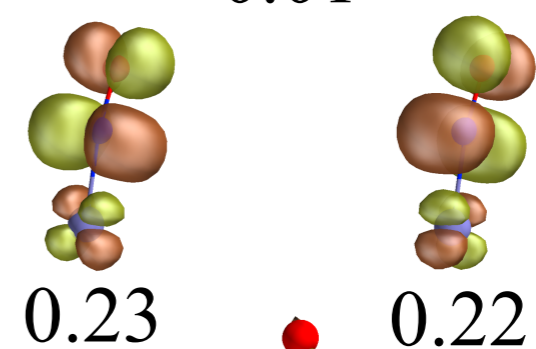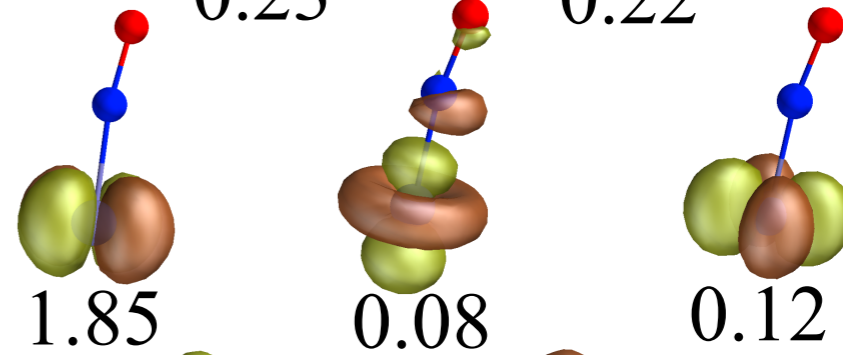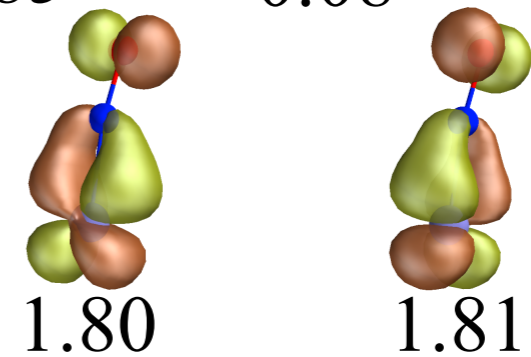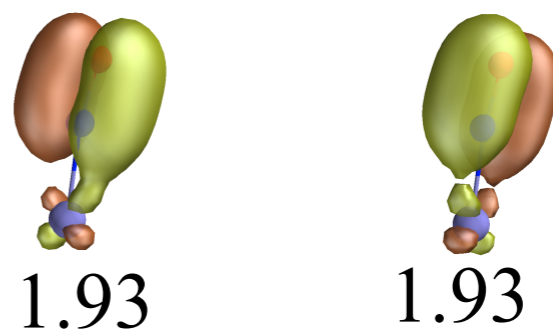

CAS(10,12)

Supplement: SC-017-D6SC00669H-s001 [file SC-017-D6SC00669H-s001.zip › SI-figures/natural-orbitals.004.pdf]

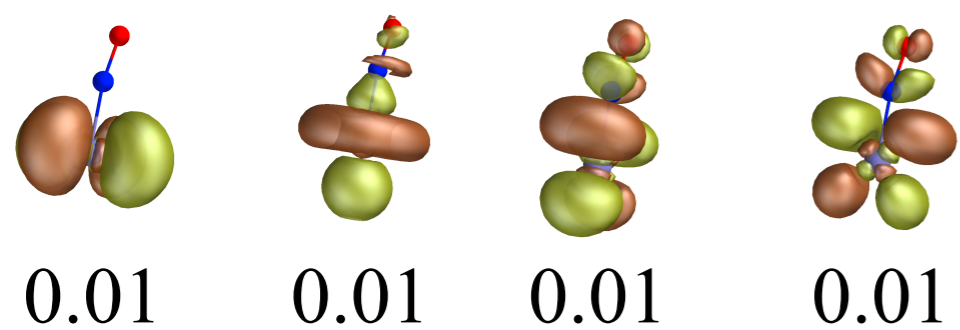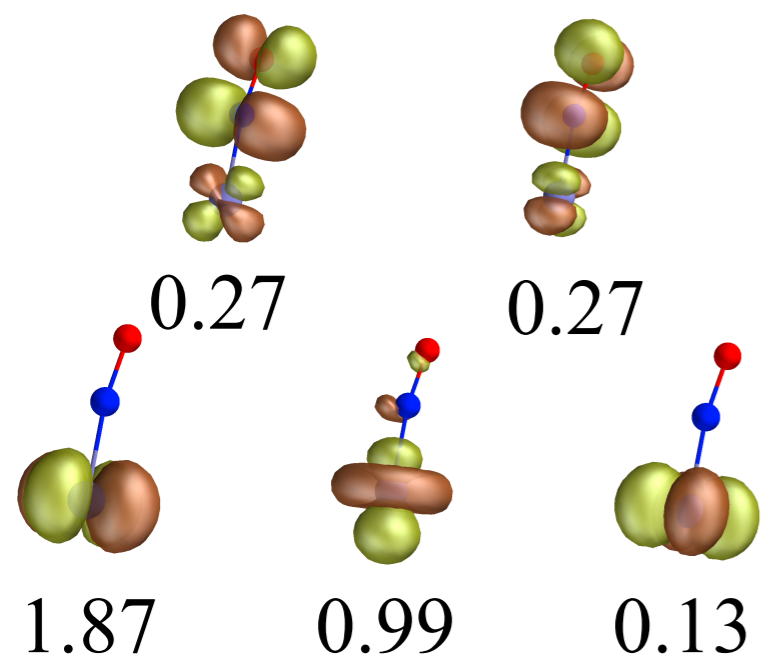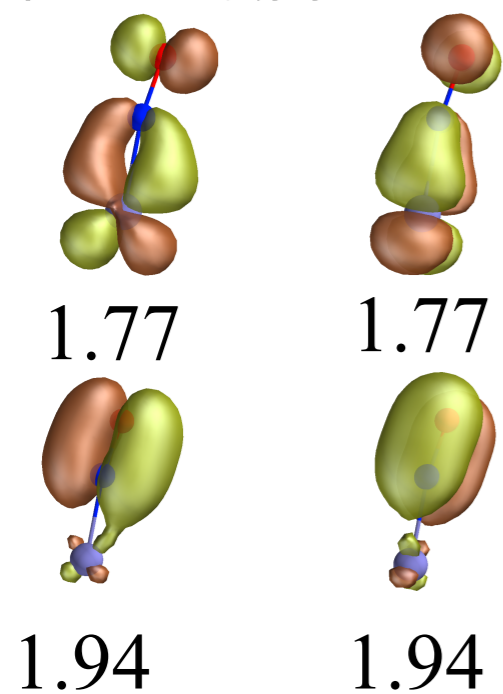

CAS(11,13)

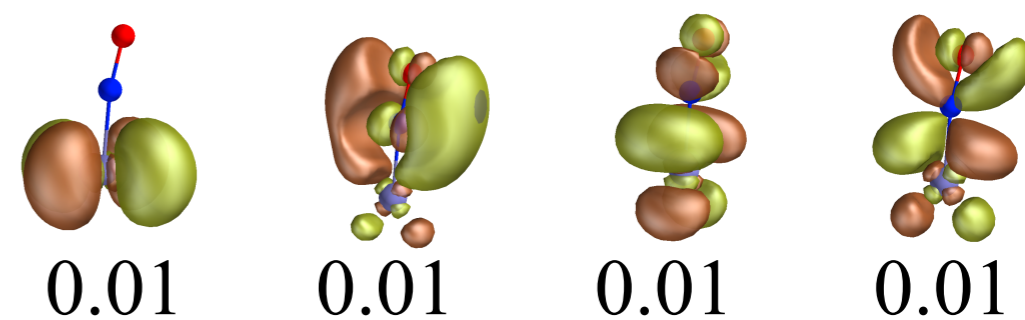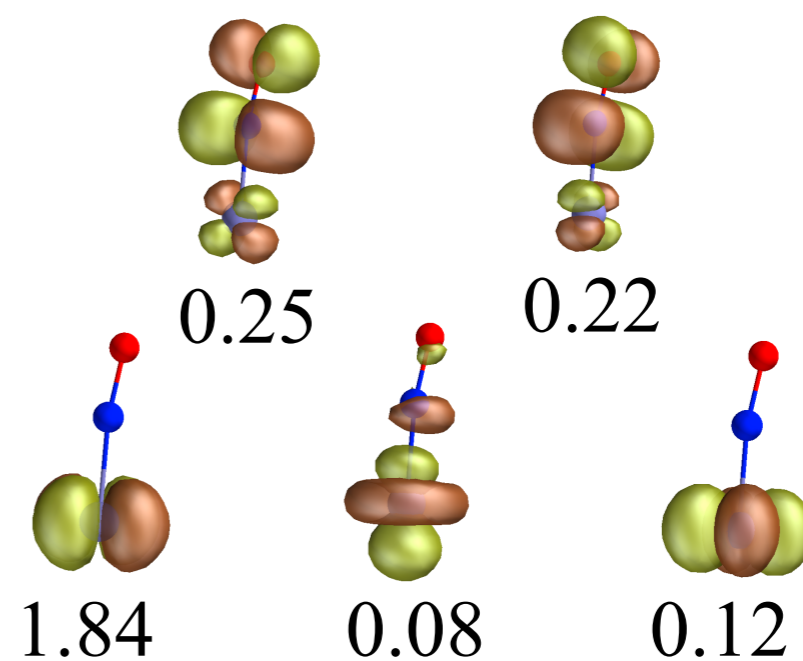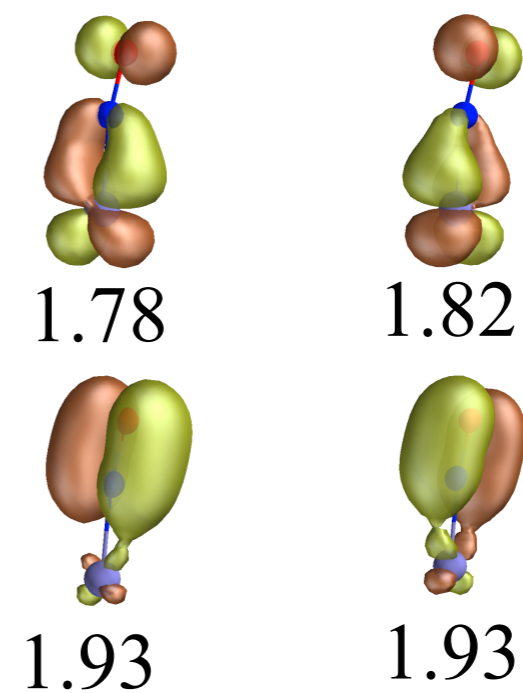

CAS(10,13)

Supplement: SC-017-D6SC00669H-s001 [file SC-017-D6SC00669H-s001.zip › SI-figures/natural-orbitals.005.pdf]

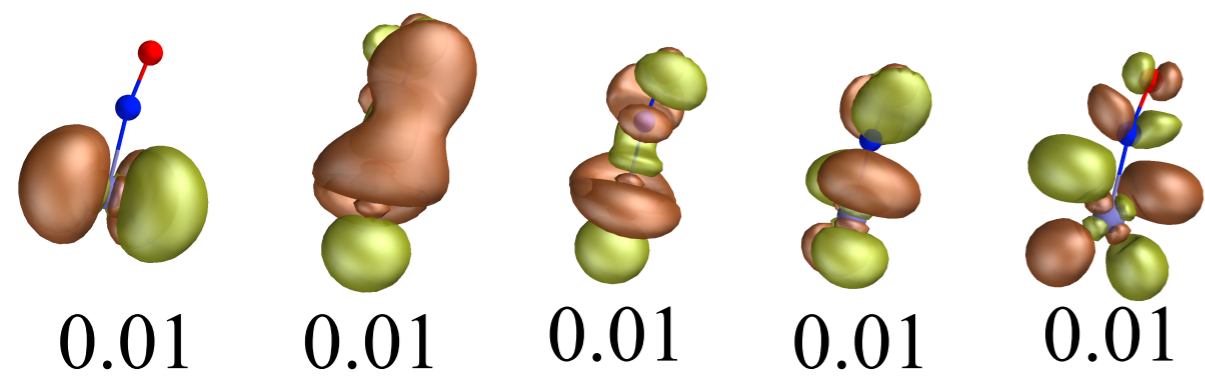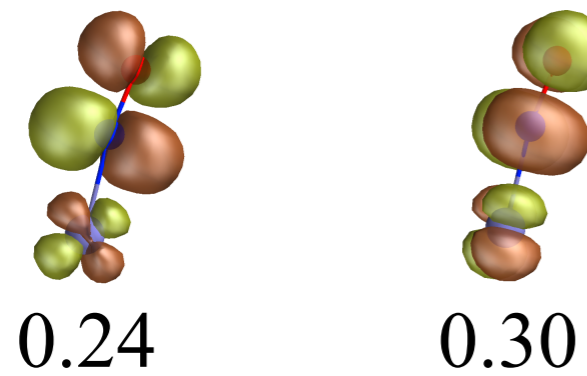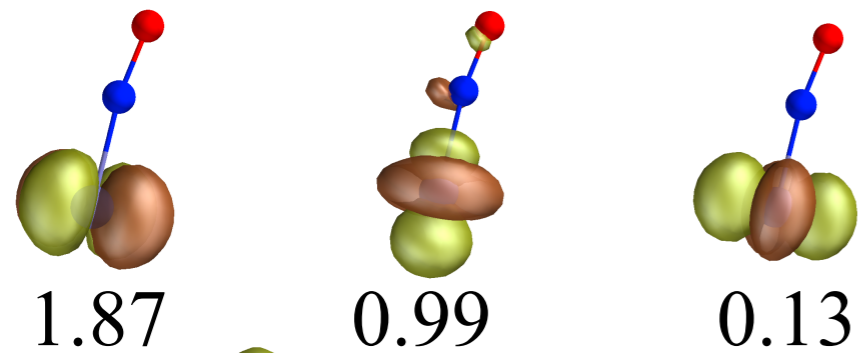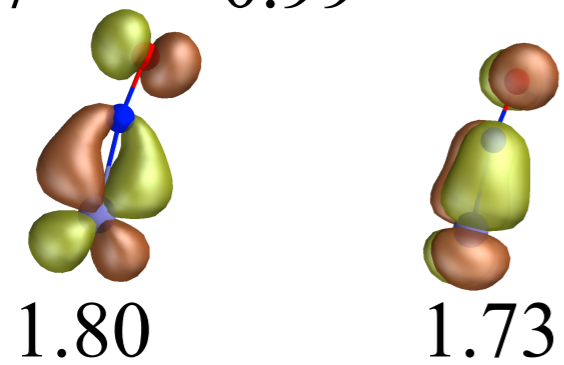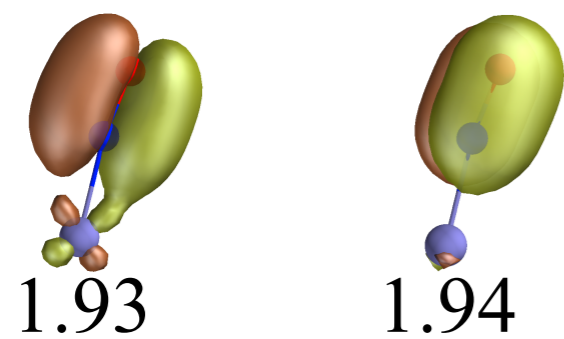

CAS(11,14)

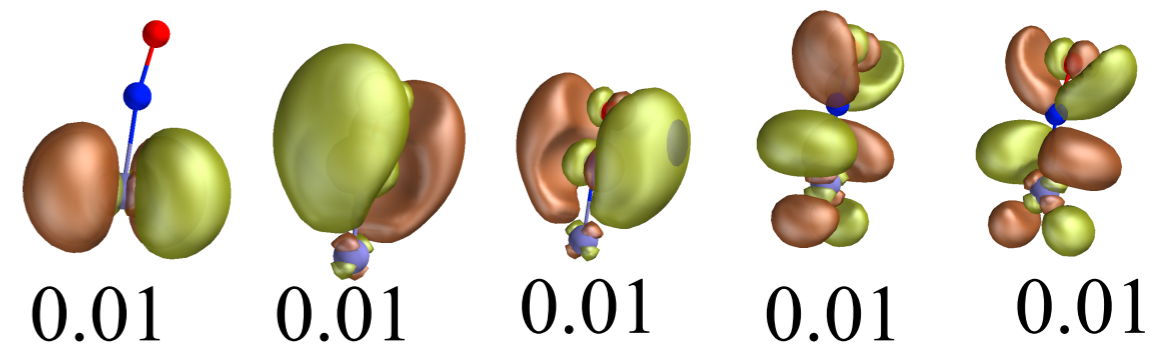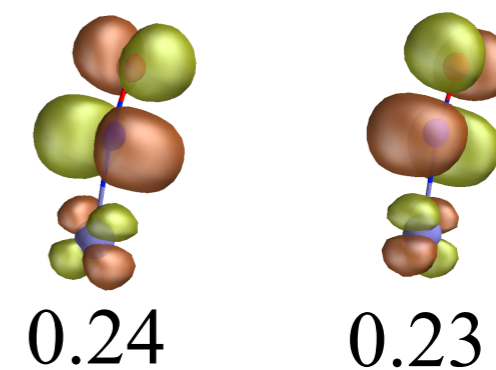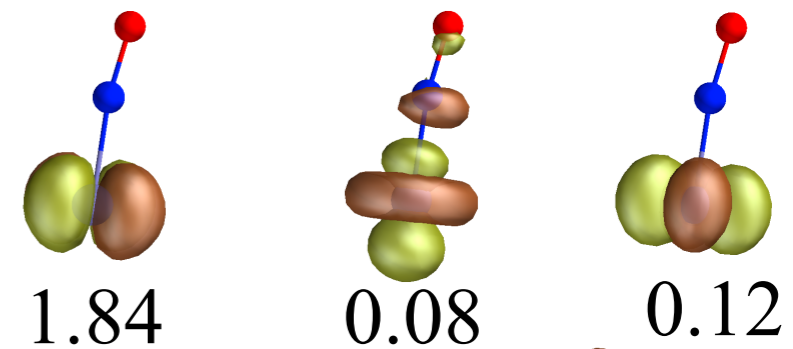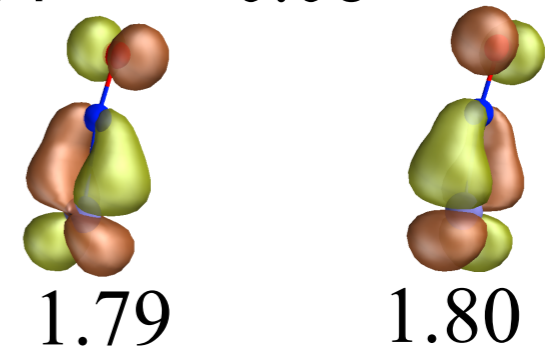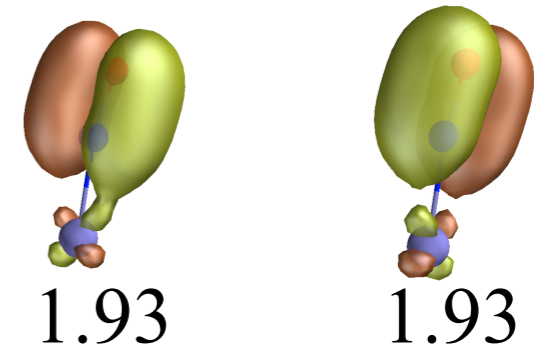

CAS(10,14)

Supplement: SC-017-D6SC00669H-s001 [file SC-017-D6SC00669H-s001.zip › SI-figures/natural-orbitals.006.pdf]

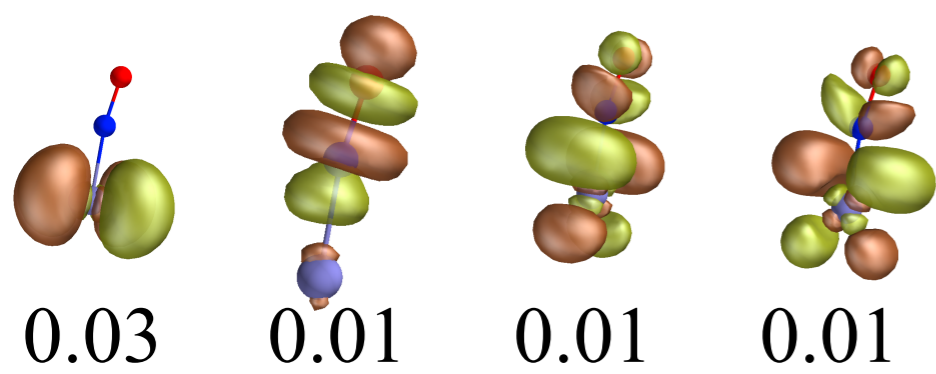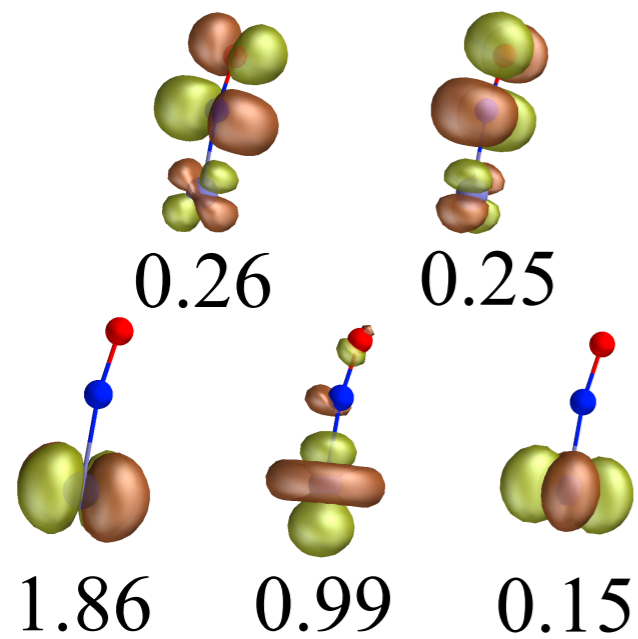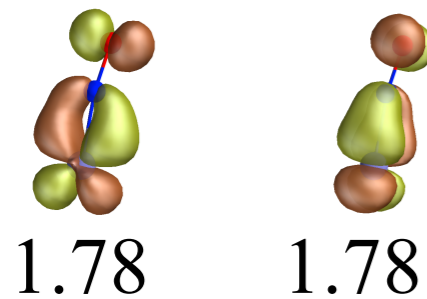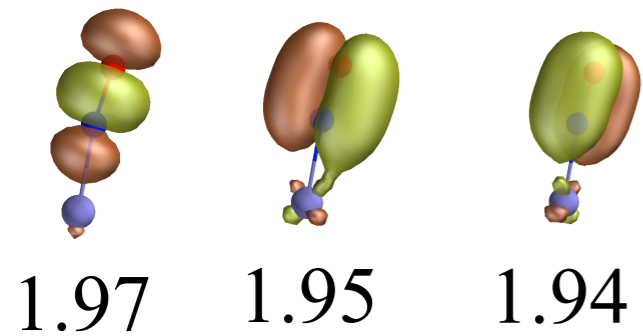

CAS(13,14)

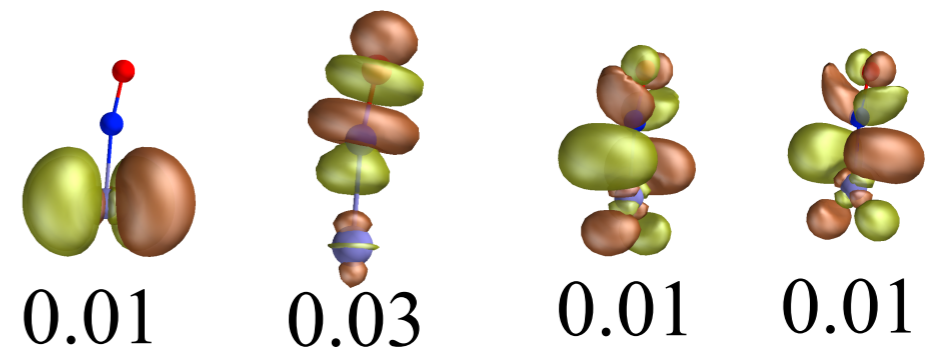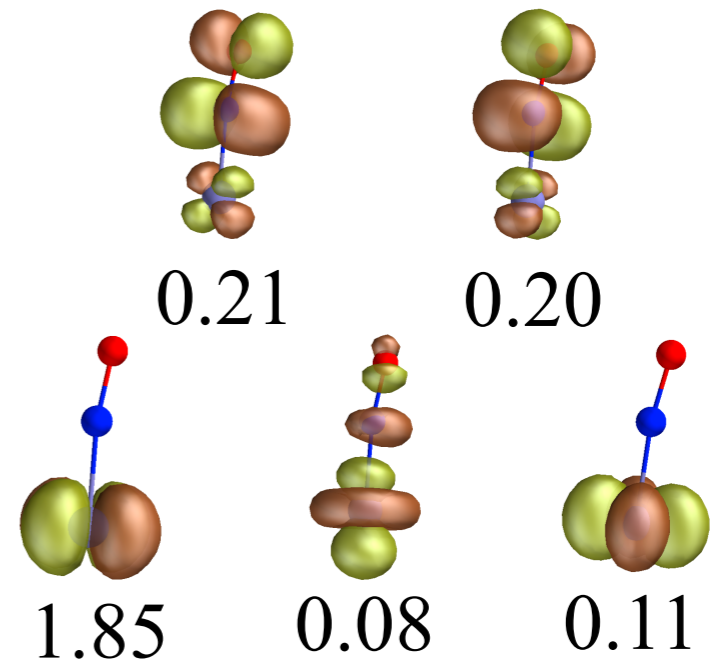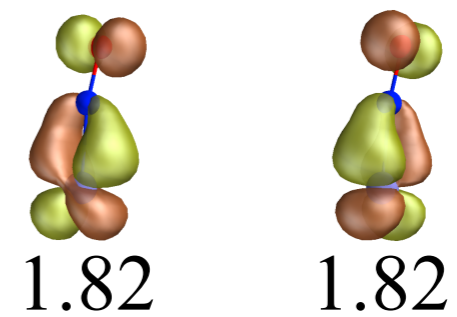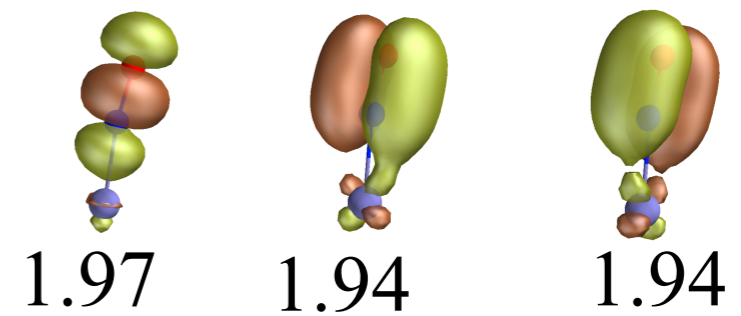

CAS(12,14)

Supplement: SC-017-D6SC00669H-s001 [file SC-017-D6SC00669H-s001.zip › SI-figures/natural-orbitals.007.pdf]

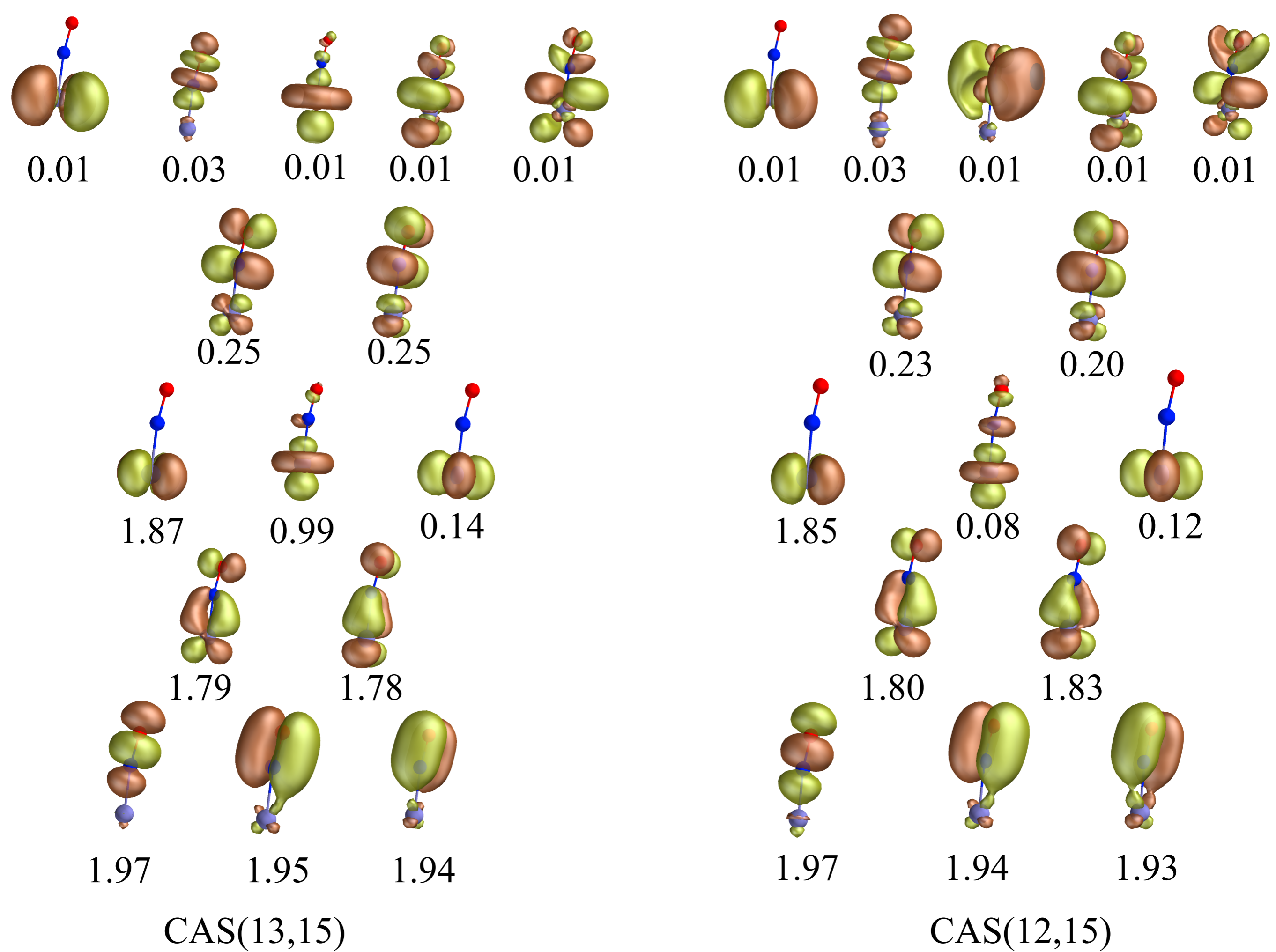

Supplement: SC-017-D6SC00669H-s001 [file SC-017-D6SC00669H-s001.zip › SI-figures/natural-orbitals.008.pdf]
