# Supplementary material for: Metal-centered X-ray absorption and emission spectroscopy of iron corroles: implications for ligand non-innocence
Source: Chem Sci. 2026 Mar 10;17(18):9230–45. doi: 10.1039/d6sc00669h (PMC13001668; doi:10.1039/d6sc00669h)
Supplement: SC-017-D6SC00669H-s002 [file SC-017-D6SC00669H-s002.pdf]

Supplementary Information (SI) for Chemical Science.
This journal is © The Royal Society of Chemistry 2026

Journal Name

ARTICLE TYPE

Cite this: DOI: 00.0000/xxxxxxxxxx

Metal-Centered X-ray Absorption and Emission Spectroscopy of Iron Corroles: Implications for Ligand Non-Innocence

Meiyuan Guo,[a†*] Abraham B. Alemayehu,[b] Augustin Braun,[c] Sang-Jun Lee,[a] Dimosthenis Sokaras,[a] Edward I. Solomon,[c*] Abhik Ghosh,[b*] and Thomas Kroll[a*]

Received Date
Accepted Date

DOI: 00.0000/xxxxxxxxxx

[a] SSRL, SLAC National Accelerator Laboratory, Menlo Park, California 94025, USA
[b] Institute of Chemistry, University of Tromsø, 9037 Tromsø, Norway
[c] Department of Chemistry, Stanford University, Stanford, California 94305, United States.
[†] Current address: Department of Chemistry - Ångström Laboratory, Uppsala University, Sweden
[*] E-mail: meiyuan.guo@kemi.uu.se, solomone@stanford.edu, abhik.ghosh@uit.no, tkroll@slac.stanford.edu

† Electronic Supplementary Information (ESI) available: [details of any supplementary information available should be included here]. See DOI: 00.0000/00000000.

List of Figures

S1 The valence active orbitals for the multiconfigurational calculations CAS(11;9) of reduced Fe[TPC](NO) for the doublet ground state. 3
S2 The valence active orbitals for the multiconfigurational calculations CAS(10;8) of Fe[TPC]Cl. 4
S3 Orbital contributions to the electric quadrupole intensity of the calculated Fe K pre-edge XAS of Fe[TPC]Cl (left) and Fe[TPC](NO) (right). 5
S4 The calculated Fe L-edge XAS of Fe[TPC]Cl with different crystal structures. 6
S5 The orbital contribution to the RAS calculated Fe L3-edge XAS of Fe[TPC]Cl. 7
S6 The orbital contribution analysis to the RAS calculated L3-edge XAS of Fe[TPC]Cl with transitions with ΔS=0 (singlet), +1 (triplet), and +2 (quintet). The integrated area is indicated. 8
S7 The orbital contribution to the RAS calculated Fe L3-edge XAS of Fe[TPC](NO). 9
S8 The orbital contribution analysis to the RAS calculated L3-edge XAS of Fe[TPC](NO) with transitions with ΔS=0 (singlet), +1 (triplet), and +2 (quintet). The integrated area is indicated. 10
S9 Representative ground-state natural and localized orbitals involved in orbital colvalent interactions for Fe[TPC](NO): $(3dxz, \pi^*_{NO})$ and $(3dxz, \pi^*_{NO})^*$, $(3dyz, \pi^*_{NO})$ and $(3dyz, \pi^*_{NO})^*$ and $(\pi_{cor}, 3dz^2)$ and $(\pi_{cor}, 3dz^2)^*$ 11
S10 Representative ground-state natural and localized orbitals involved in orbital colvalent interactions for Fe[TPC]Cl: $(\pi_{cor}, 3dz^2)$ and $(\pi_{cor}, 3dz^2)^*$ 12
S11 CASSCF natural orbitals and occupation numbers for $[FeNO]^{2+}$ (left) and $[FeNO]^{3+}$ (right) in its lowest spin state for different active spaces CAS(7;7) and CAS(6;7) considered in this work. An isosurface value of 0.05 was chosen. 13
S12 CASSCF natural orbitals and occupation numbers for $[FeNO]^{2+}$ (left) and $[FeNO]^{3+}$ (right) in its lowest spin state for different active spaces CAS(11;9) and CAS(10;9) considered in this work. An isosurface value of 0.05 was chosen. 14
S13 CASSCF natural orbitals and occupation numbers for $[FeNO]^{2+}$ (left) and $[FeNO]^{3+}$ (right) in its lowest spin state for different active spaces CAS(11;11) and CAS(10;11) considered in this work. An isosurface value of 0.05 was chosen. . . 15
S14 CASSCF natural orbitals and occupation numbers for $[FeNO]^{2+}$ (left) and $[FeNO]^{3+}$ (right) in its lowest spin state for different active spaces CAS(11;12) and CAS(10;12) considered in this work. An isosurface value of 0.05 was chosen. . . 16
S15 CASSCF natural orbitals and occupation numbers for $[FeNO]^{2+}$ (left) and $[FeNO]^{3+}$ (right) in its lowest spin state for different active spaces CAS(11;13) and CAS(10;13) considered in this work. An isosurface value of 0.05 was chosen. . . 17
S16 CASSCF natural orbitals and occupation numbers for $[FeNO]^{2+}$ (left) and $[FeNO]^{3+}$ (right) in its lowest spin state for different active spaces CAS(11;14) and CAS(10;14) considered in this work. An isosurface value of 0.05 was chosen. . . 18
S17 CASSCF natural orbitals and occupation numbers for $[FeNO]^{2+}$ (left) and $[FeNO]^{3+}$ (right) in its lowest spin state for different active spaces CAS(13;14) and CAS(12;14) considered in this work. An isosurface value of 0.05 was chosen. . . 19
S18 CASSCF natural orbitals and occupation numbers for $[FeNO]^{2+}$ (left) and $[FeNO]^{3+}$ (right) in its lowest spin state for different active spaces CAS(13;15) and CAS(12;15) considered in this work. An isosurface value of 0.05 was chosen. . . 20
S19 The comparison of RAS calculated Fe K pre-edge XAS of $[FeNO]^{3+}$ unit and molecular Fe[TPC](NO). 21

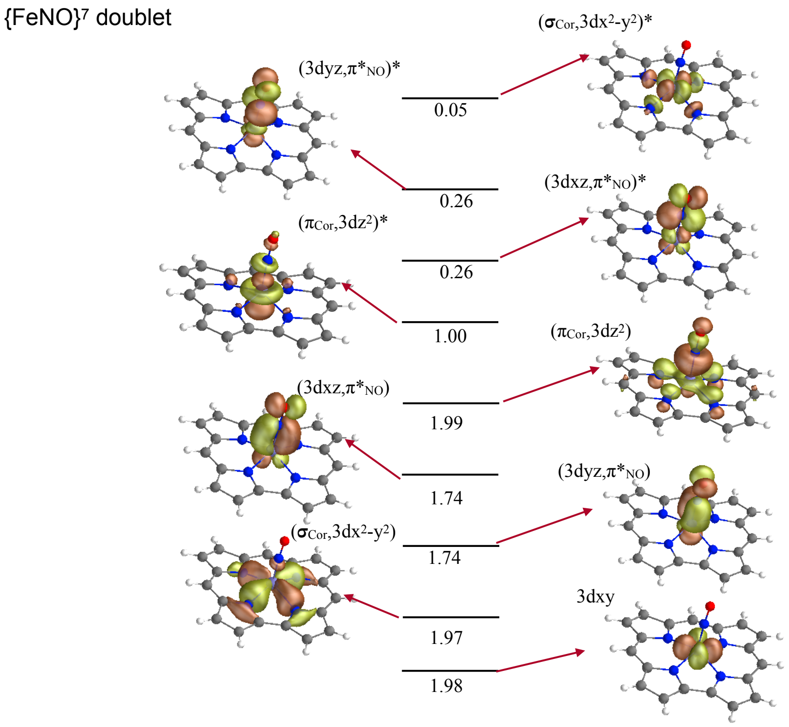

Fig. S1 The valence active orbitals for the multiconfigurational calculations CAS(11;9) of reduced Fe[TPC](NO) for the doublet ground state.

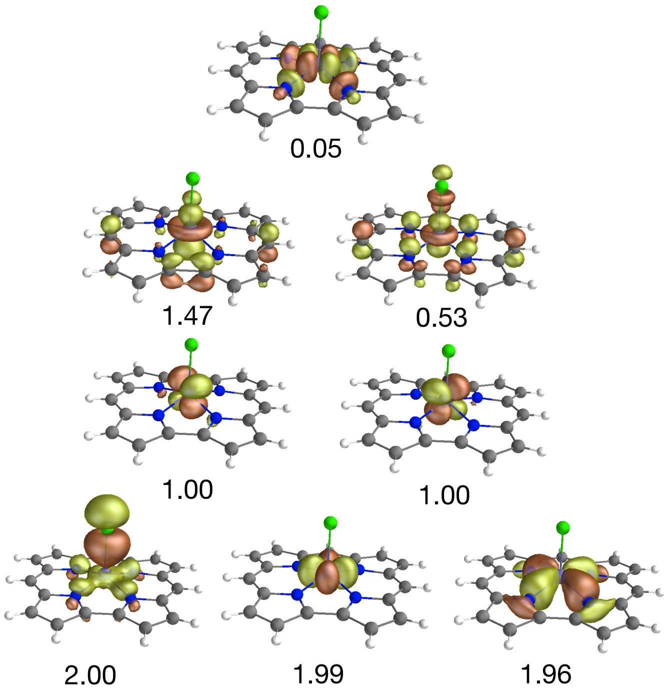

Fig. S2 The valence active orbitals for the multiconfigurational calculations CAS(10;8) of Fe[TPC]Cl.

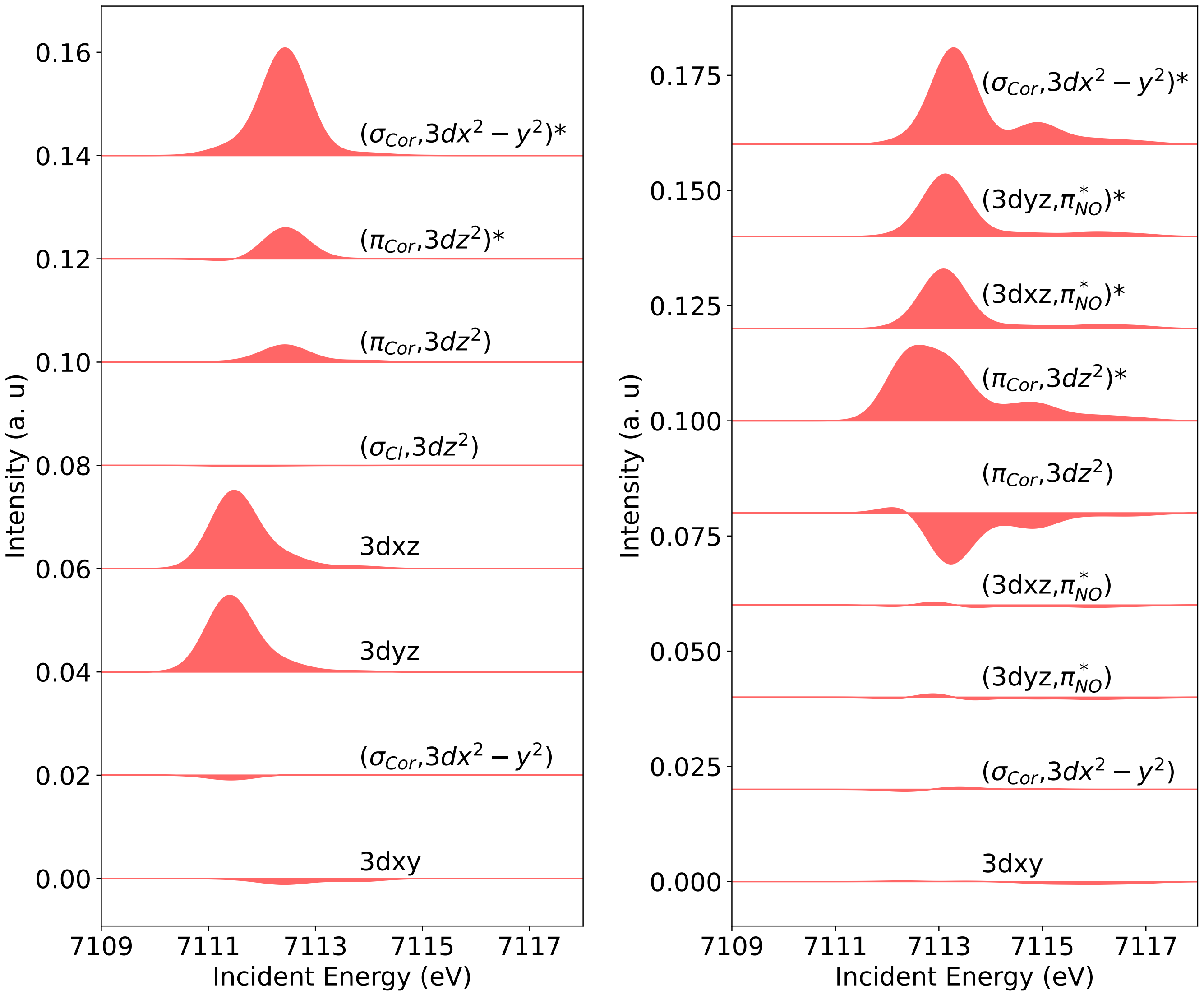

Fig. S3 Orbital contributions to the electric quadrupole intensity of the calculated Fe K pre-edge XAS of Fe[TPC]Cl (left) and Fe[TPC](NO) (right).

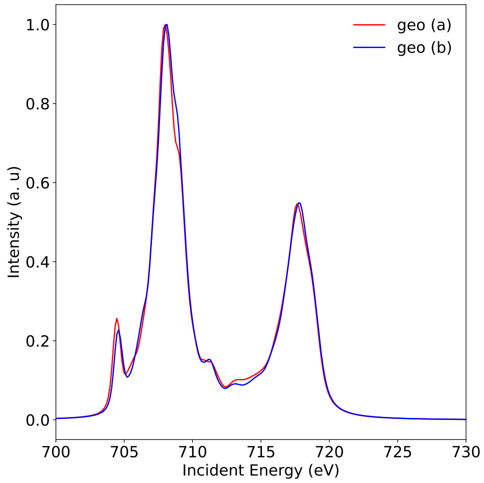

Fig. S4 The calculated Fe L-edge XAS of Fe[TPC]Cl with different crystal structures.

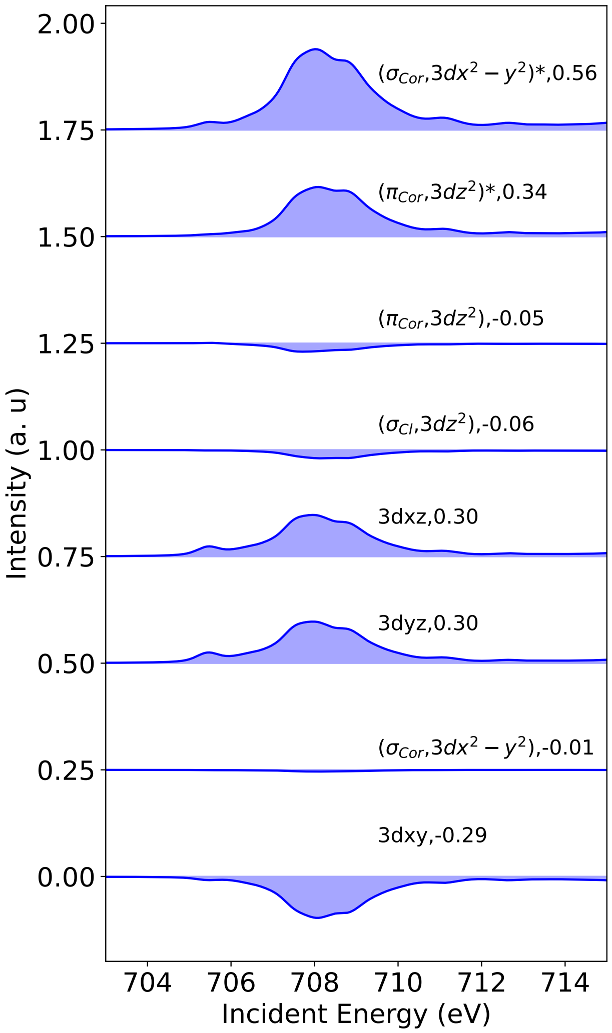

Fig. S5 The orbital contribution to the RAS calculated Fe L3-edge XAS of Fe[TPC]Cl.

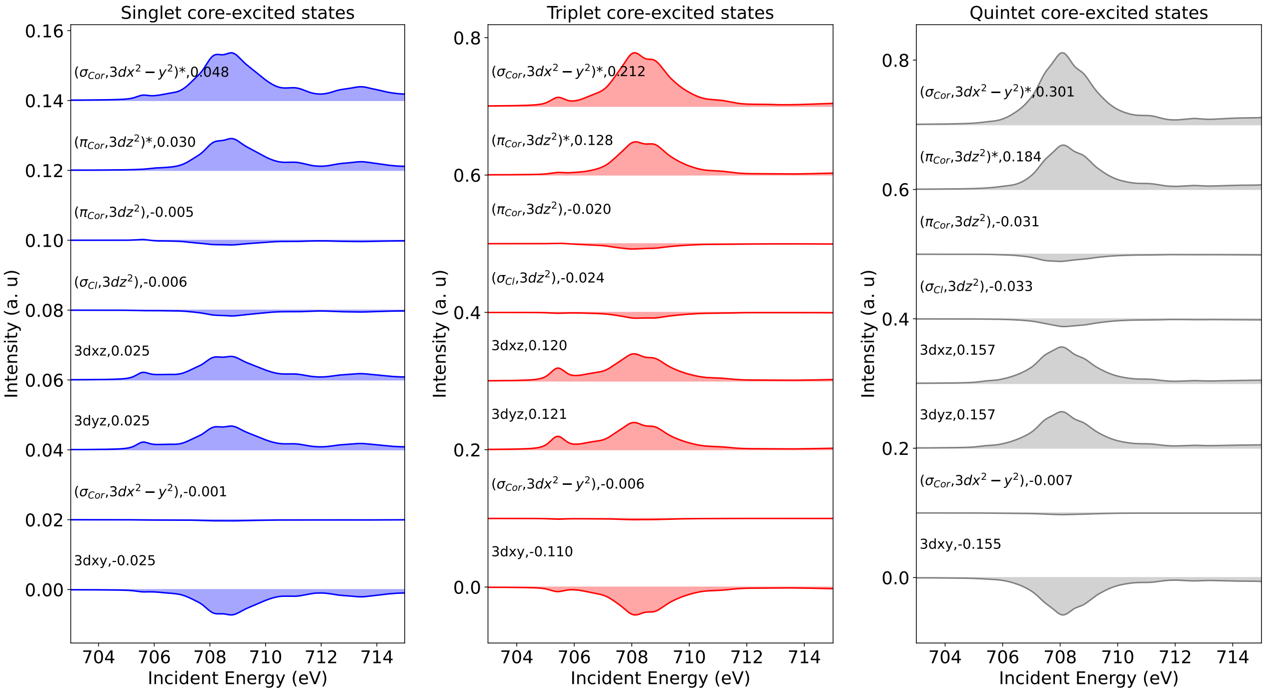

Fig. S6 The orbital contribution analysis to the RAS calculated L3-edge XAS of Fe[TPC]Cl with transitions with ΔS=0 (singlet), +1 (triplet), and +2 (quintet). The integrated area is indicated.

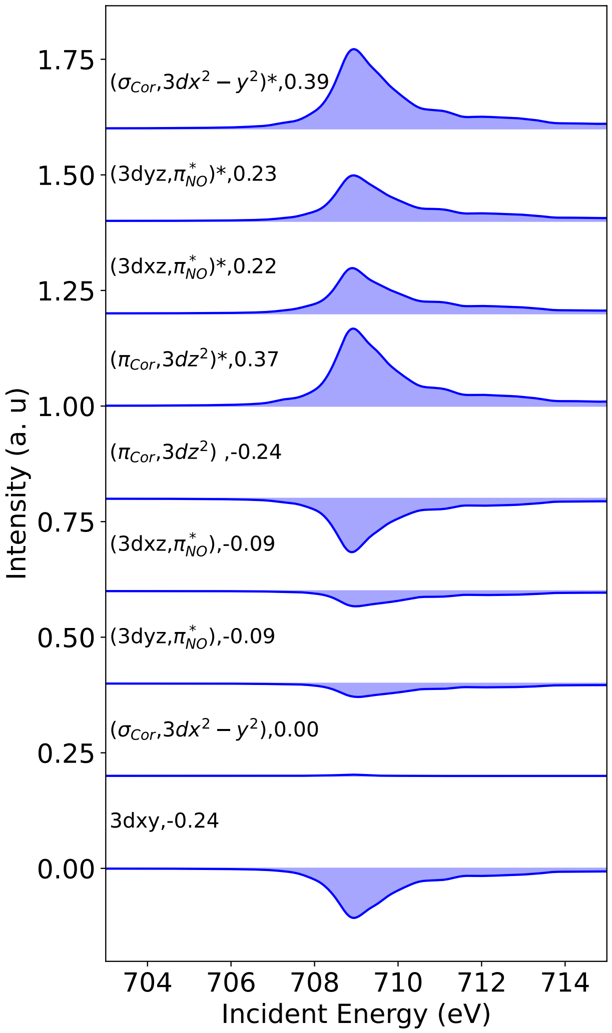

Fig. S7 The orbital contribution to the RAS calculated Fe L3-edge XAS of Fe[TPC](NO).

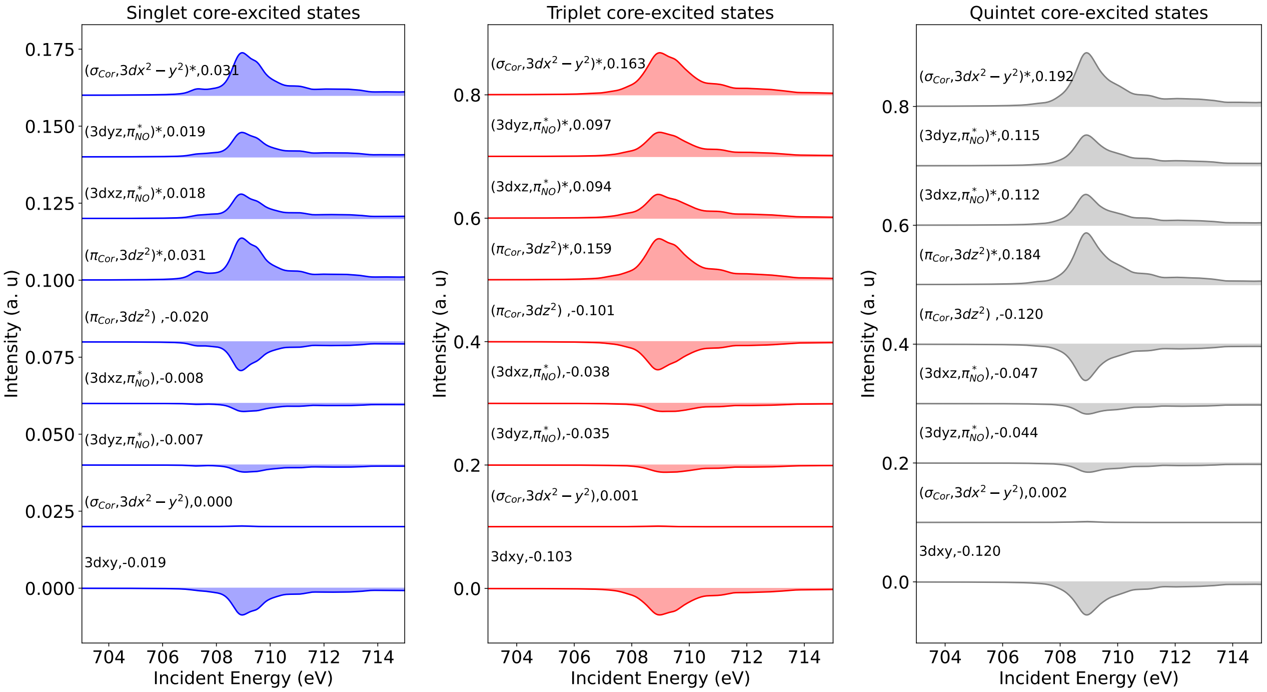

Fig. S8 The orbital contribution analysis to the RAS calculated L3-edge XAS of Fe[TPC](NO) with transitions with ΔS=0 (singlet), +1 (triplet), and +2 (quintet). The integrated area is indicated.

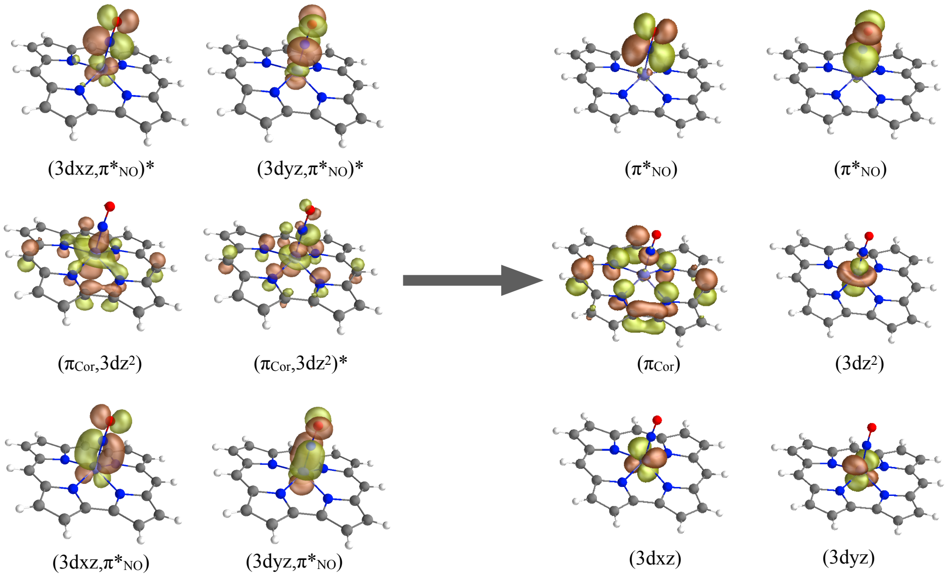

Fig. S9 Representative ground-state natural and localized orbitals involved in orbital colvalent interactions for Fe[TPC](NO): $(3dxz,\ \pi^*_{NO})$ and $(3dxz,\pi^*_{NO})^*$, $(3dyz,\ \pi^*_{NO})$ and $(3dyz,\pi^*_{NO})^*$ and $(\pi_{cor},3dz^2)$ and $(\pi_{cor},3dz^2)^*$.

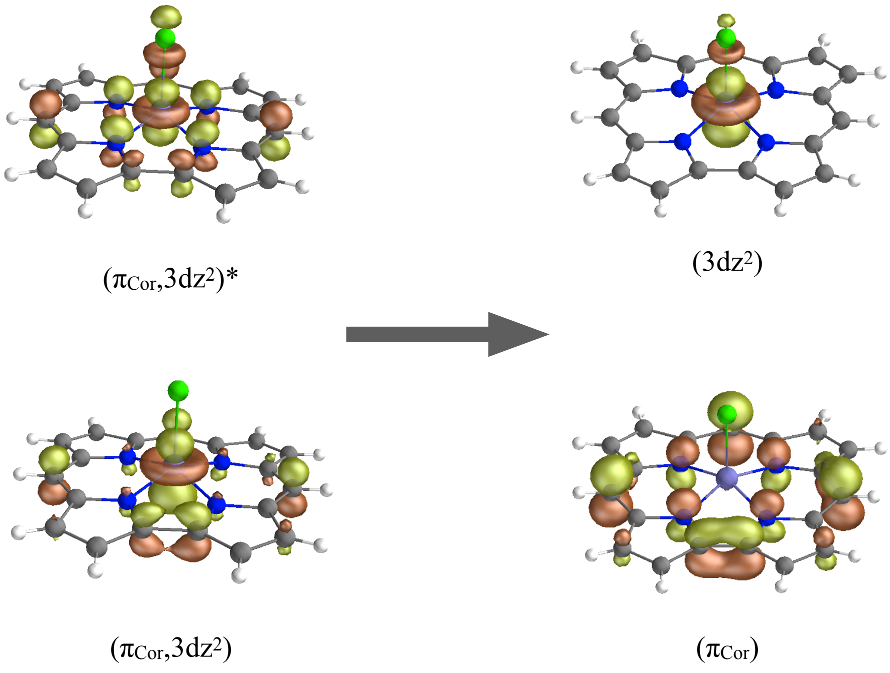

Fig. S10 Representative ground-state natural and localized orbitals involved in orbital colvalent interactions for Fe[TPC]Cl: $(\pi_{cor}, 3dz^2)$ and $(\pi_{cor}, 3dz^2)^*$
.

active space	$\{FeNO\}^{2+}$ 222a000	$\{FeNO\}^{3+}$ 2220000
CAS(7;7)/CAS(6;7)	0.59	0.63
CAS(11;9)/CAS(10;9)	0.54	0.58
CAS(11;11)/CAS(10;11)	0.66	0.63
CAS(11;12)/CAS(10;12)	0.66	0.71
CAS(11;13)/CAS(10;13)	0.67	0.70
CAS(11;14)/CAS(10;14)	0.66	0.69
CAS(13;14)/CAS(12;14)	0.67	0.71
CAS(13;15)/CAS(12;15)	0.67	0.71

Table 1 The dominant configuration contributions to the ground state wavefunction for $\{FeNO\}^{2+}$ and $\{FeNO\}^{3+}$ with different active space. Configuration with selected orbitals are presented, the orbital ordering: 3dxy, (3dyz,π^*_{NO}), (3dxz,π^*_{NO}), 3dz^2, (3dxz,π^*_{NO})*, (3dyz,π^*_{NO})*, 3dx^{2-}y^2. 2: doubly occupied orbital, a: orbital occupied by an α electron. b: orbital occupied by an β electron, 0: empty orbital.

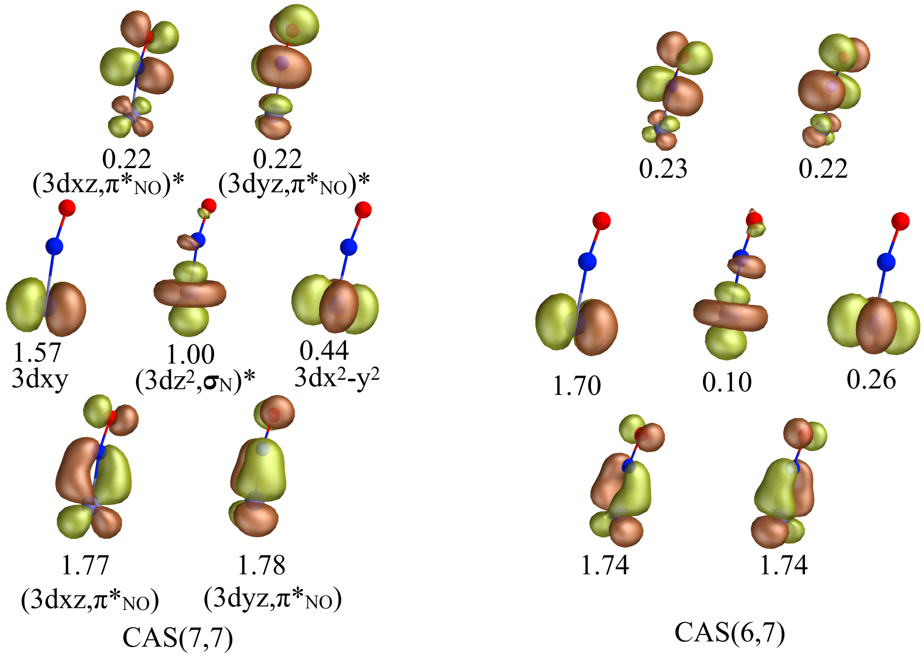

Fig. S11 CASSCF natural orbitals and occupation numbers for $[FeNO]^{2+}$ (left) and $[FeNO]^{3+}$ (right) in its lowest spin state for different active spaces CAS(7;7) and CAS(6;7) considered in this work. An isosurface value of 0.05 was chosen.

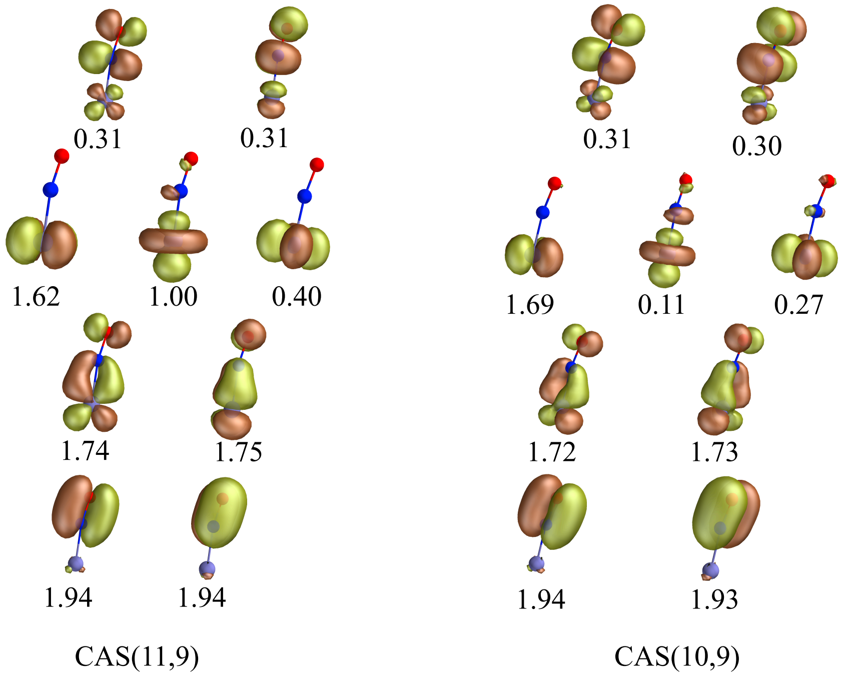

Fig. S12 CASSCF natural orbitals and occupation numbers for $[FeNO]^{2+}$ (left) and $[FeNO]^{3+}$ (right) in its lowest spin state for different active spaces CAS(11;9) and CAS(10;9) considered in this work. An isosurface value of 0.05 was chosen.

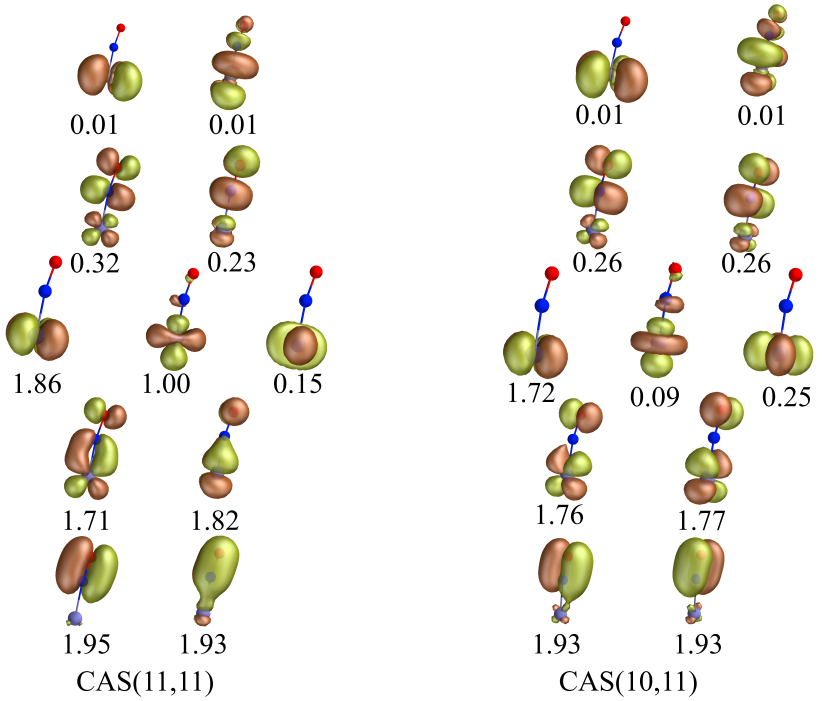

Fig. S13 CASSCF natural orbitals and occupation numbers for $[FeNO]^{2+}$ (left) and $[FeNO]^{3+}$ (right) in its lowest spin state for different active spaces CAS(11;11) and CAS(10;11) considered in this work. An isosurface value of 0.05 was chosen.

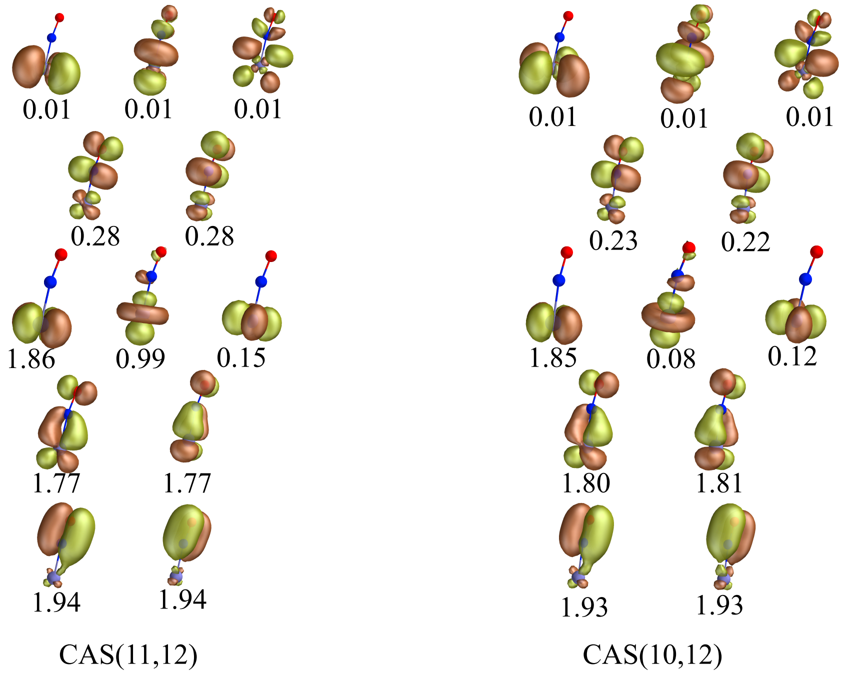

Fig. S14 CASSCF natural orbitals and occupation numbers for $[FeNO]^{2+}$ (left) and $[FeNO]^{3+}$ (right) in its lowest spin state for different active spaces CAS(11;12) and CAS(10;12) considered in this work. An isosurface value of 0.05 was chosen.

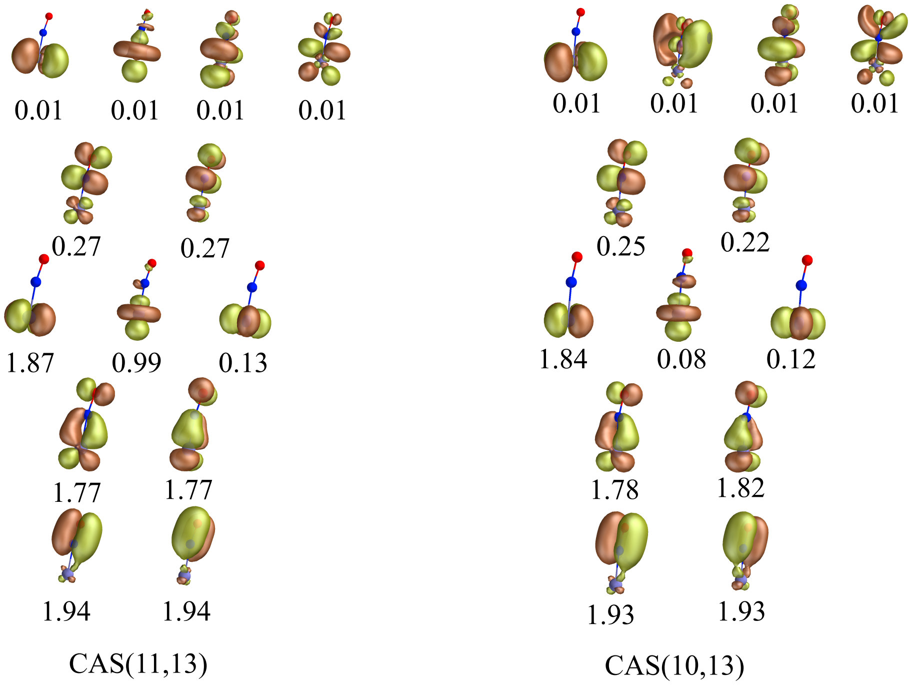

Fig. S15 CASSCF natural orbitals and occupation numbers for $[FeNO]^{2+}$ (left) and $[FeNO]^{3+}$ (right) in its lowest spin state for different active spaces CAS(11;13) and CAS(10;13) considered in this work. An isosurface value of 0.05 was chosen.

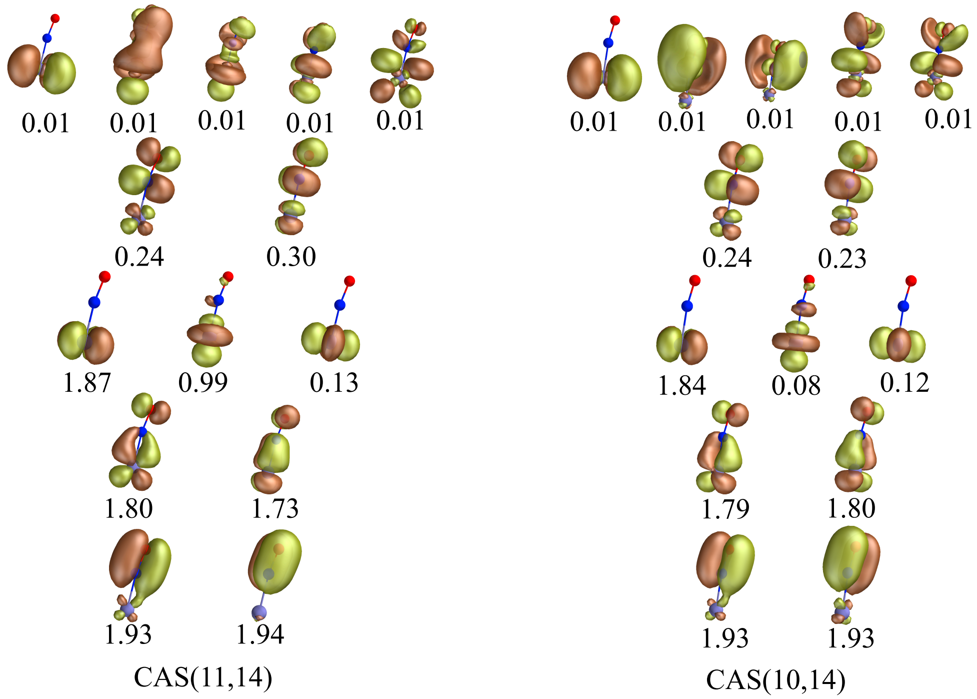

Fig. S16 CASSCF natural orbitals and occupation numbers for $[FeNO]^{2+}$ (left) and $[FeNO]^{3+}$ (right) in its lowest spin state for different active spaces CAS(11;14) and CAS(10;14) considered in this work. An isosurface value of 0.05 was chosen.

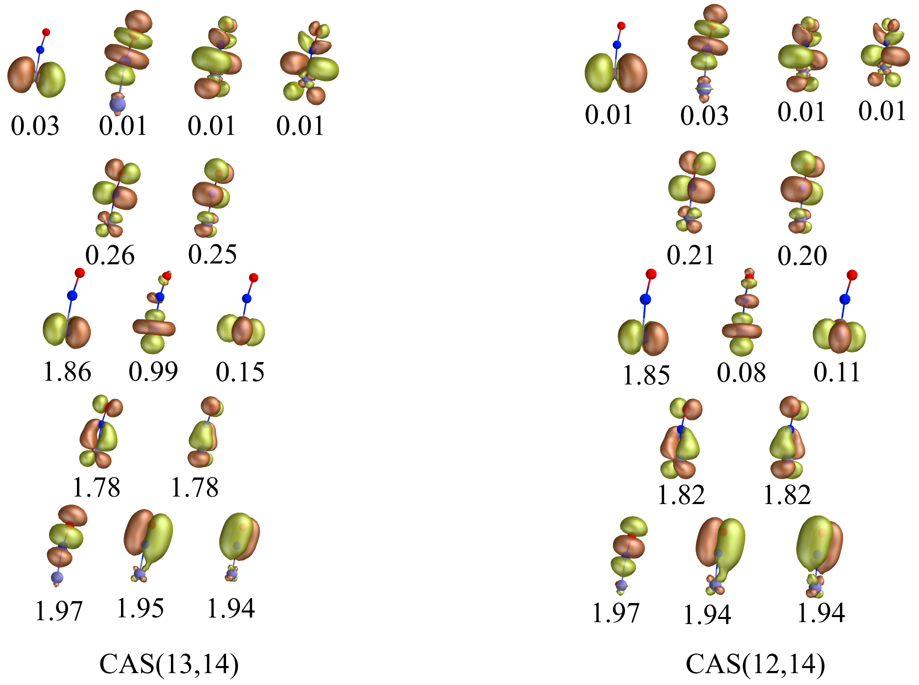

Fig. S17 CASSCF natural orbitals and occupation numbers for $[FeNO]^{2+}$ (left) and $[FeNO]^{3+}$ (right) in its lowest spin state for different active spaces CAS(13;14) and CAS(12;14) considered in this work. An isosurface value of 0.05 was chosen.

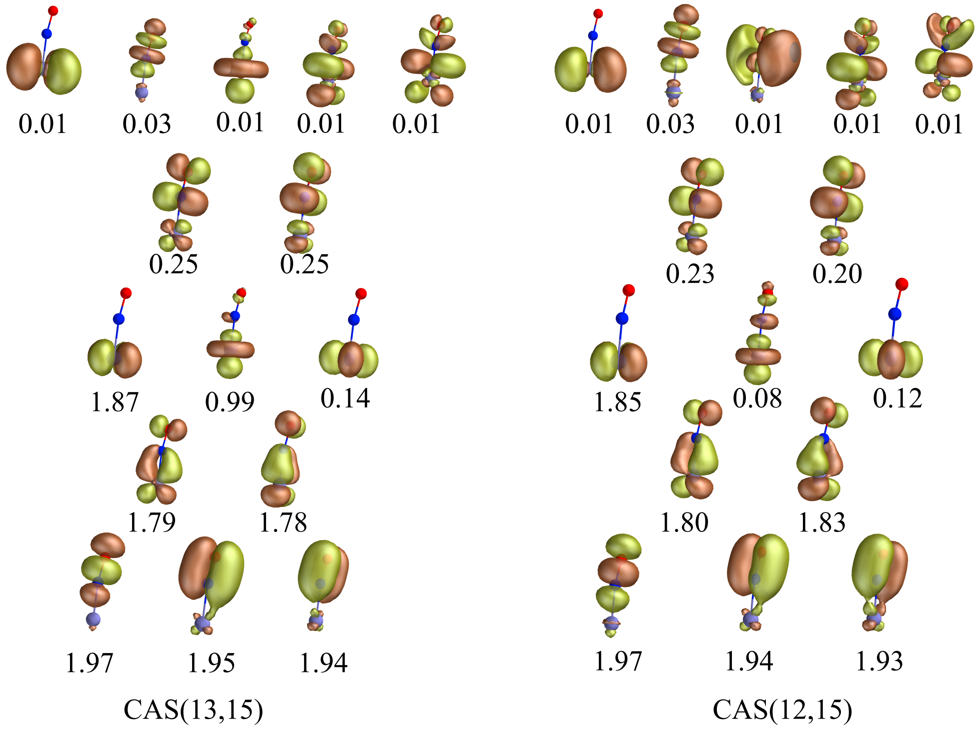

Fig. S18 CASSCF natural orbitals and occupation numbers for $[FeNO]^{2+}$ (left) and $[FeNO]^{3+}$ (right) in its lowest spin state for different active spaces CAS(13;15) and CAS(12;15) considered in this work. An isosurface value of 0.05 was chosen.

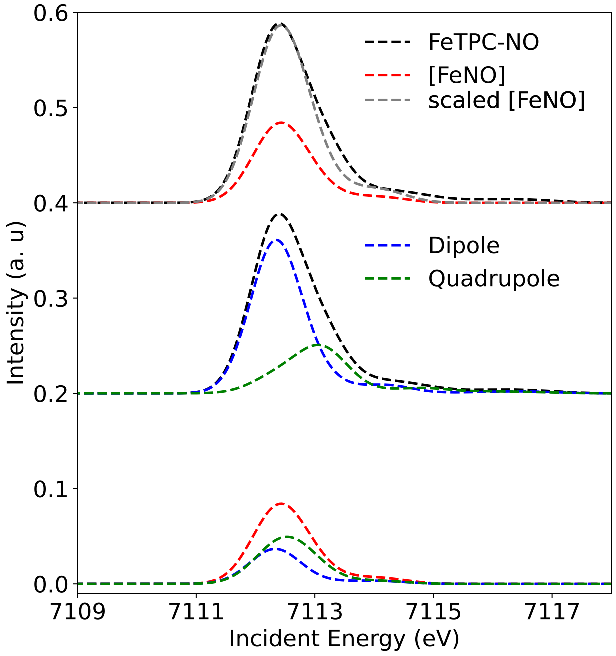

Fig. S19 The comparison of RAS calculated Fe K pre-edge XAS of $[FeNO]^{3+}$ unit and molecular Fe[TPC](NO).

Coordinates of the compounds used in the calculations

The crystal structures for Fe[TPC](NO) and Fe[TPC]Cl are available in cited references, the structures from DFT calculations are enclosed below.

DFT close-shell singlet structure of Fe[TPC](NO)

Fe	0.00506	0.00885	-0.16120
O	0.55849	-0.11870	2.63741
N	-1.39104	-1.20776	-0.68717
N	1.27837	-1.40251	-0.54777
N	1.28784	1.42332	-0.51098
N	-1.37576	1.24048	-0.71435
N	0.06770	-0.01458	1.57609
C	-2.62563	-0.69192	-0.89168
C	-3.54018	-1.76163	-1.11904
C	-2.80780	-2.93033	-1.05803
C	-1.44212	-2.57264	-0.78764
C	-0.27804	-3.33869	-0.73405
C	1.01365	-2.75303	-0.62312
C	2.26041	-3.45830	-0.60157
C	3.26334	-2.52938	-0.53532
C	2.64594	-1.23910	-0.49601
C	3.30013	0.00516	-0.46549
C	2.65438	1.25412	-0.49879
C	3.27570	2.54350	-0.52311
C	2.27441	3.47601	-0.55911
C	1.02511	2.77452	-0.54198
C	-0.26575	3.36878	-0.61903
C	-1.43041	2.60998	-0.73028
C	-2.80824	2.98035	-0.90228
C	-3.54274	1.81566	-1.00735
C	-2.62063	0.73609	-0.88263
H	-4.59323	-1.66320	-1.32598
H	-3.17399	-3.93104	-1.21287
H	2.37153	-4.52740	-0.64529
H	4.32309	-2.71514	-0.52473
H	4.33598	2.72609	-0.52622
H	2.38601	4.54495	-0.61024
H	-3.18610	3.98716	-0.95383
H	-4.60616	1.72866	-1.15918
H	-0.34260	4.39755	-0.59160
H	4.33088	0.00166	-0.41488
H	-0.35640	-4.36684	-0.77637

DFT broken-symmetry singlet structure of Fe[TPC](NO)

Fe	0.000000	0.000000	0.000000
N	-0.001453	0.066249	1.709147
O	-0.020318	0.313020	2.845265
N	-1.403596	1.209571	-0.493698
N	-1.405497	-1.223226	-0.480088
N	1.277260	1.400774	-0.435050
N	1.277374	-1.414639	-0.425763
C	3.270527	-0.008280	-0.464201
C	-2.649009	0.706122	-0.648707
C	-2.649537	-0.721568	-0.640637
C	-3.560414	1.790957	-0.824890
C	-3.562606	-1.807997	-0.804794
C	-2.817111	2.951311	-0.775793
C	-2.819755	-2.967558	-0.743486
C	-1.445607	2.578373	-0.576602
C	-1.447580	-2.592043	-0.548911
C	-0.273453	3.312801	-0.550362
C	-0.275388	-3.326101	-0.516466
C	1.013836	2.745379	-0.499121
C	1.012570	-2.758712	-0.472687
C	2.258603	3.453448	-0.571678
C	2.256662	-3.470165	-0.533938
C	3.261983	2.523808	-0.555047
C	3.261138	-2.541888	-0.527188
C	2.641622	1.235491	-0.470631
C	2.641047	-1.251885	-0.460107
H	4.352831	-0.008314	-0.497404
H	-0.334202	4.391097	-0.621456
H	-0.336423	-4.405128	-0.575808
H	-4.623035	1.706995	-0.986036
H	-4.625265	-1.725198	-0.966288
H	-3.180111	3.960350	-0.891112
H	-3.182956	-3.977754	-0.847576
H	2.354374	4.525407	-0.645808
H	2.350987	-4.543163	-0.593770
H	4.325101	2.697429	-0.608767
H	4.324222	-2.717291	-0.575525
